# LACTB induces cancer cell death through the activation of the intrinsic caspase-independent pathway in breast cancer

**DOI:** 10.1007/s10495-022-01775-4

**Published:** 2022-10-25

**Authors:** Juan M. Gonzalez-Morena, Sara Escudeiro-Lopes, Jessica Mariane Ferreira-Mendes, Pavel Jakoube, Valentina Cutano, Judith Vinaixa-Forner, Petra Kralova Viziova, Andrea Hartmanova, Radislav Sedlacek, Susana Machado, Beata Malcekova, Zuzana Keckesova

**Affiliations:** 1 Institute of Organic Chemistry and Biochemistry, Czech Academy of Sciences, Prague, Czech Republic; 2 Department of Cell Biology, Faculty of Science, Charles University, Prague, Czech Republic; 3 The Czech Center for Phenogenomics, Institute of Molecular Genetics of the Czech Academy of Sciences, Vestec, Czech Republic

**Keywords:** LACTB, Apoptosis, Cell death, Caspases, Mitochondria, Breast cancer, Cell cycle arrest

## Abstract

**Background:**

LACTB was recently identified as a mitochondrial tumour suppressor that negatively affects cancer cell proliferation by inducing cell death and/or differentiation, depending on the cell type and tissue. However, the detailed mechanism underlying the LACTB-induced cancer cell death is largely unknown.

**Methods:**

We used cell-based, either in 2D or 3D conditions, and in vivo experiments to understand the LACTB mechanisms. In this regard, protein array followed by an enrichment analysis, cell proliferation assays using different compounds, western blot analysis, flow cytometry and immunofluorescence were performed. Differences between quantitative variables following normal distribution were valuated using Student t test for paired or no-paired samples according to the experiment. For in vivo experiments differences in tumour growth were analyzed by 2-way ANOVA.

**Results:**

We show, that LACTB expression leads to cell cycle arrest in G1 phase and increase of DNA oxidation that leads to activation of intrinsic caspase-independent cell death pathway. This is achieved by an increase of mitochondrial reactive oxygen species since early time points of LACTB induction.

**Conclusion:**

Our work provides a deeper mechanistic insight into LACTB-mediated cancer-cell death and shows the dynamics of the cellular responses a particular tumor suppressive stimulus might evoke under different genetic landscapes.

**Supplementary Information:**

The online version contains supplementary material available at 10.1007/s10495-022-01775-4.

## Introduction

Cancer initiation and progression requires the deactivation of tumor suppressive genes and proteins and/or activation of proto-oncogenes [1]. One of the latest tumour suppressors, which was identified while researching tissues where cancer rarely occurs, is the serine beta-lactamase-like (LACTB) protein [2]. LACTB is localized in the mitochondrial intermembrane space and is a homologue of bacterial beta-lactamases with the addition of a mitochondrial localization sequence and a central region only present in eukaryotes. It was shown that this protein is able to form filamentous structures inside mitochondria [3]. LACTB was found to inhibit cancer cell proliferation in numerous tissues through modulation of cancer cell death or cancer cell differentiation/EMT, both processes being cell-type-specific [4–10]. In breast cancer, the differentiation-inducing mechanism was shown to be triggered by LACTB-induced changes in lipid metabolism and the resulting changes in the levels of mitochondrial phosphatidylethanolamines and lysophosphatidyltheanolamines [2]. However, the mechanistic insight into LACTB induced breast cancer cell-death and the signaling pathways involved in this process are currently unknown.

Apoptosis is a programmed cell-death event, which is regulated by numerous extracellular or intracellular signals, leading to activation of extrinsic or intrinsic apoptotic pathway, respectively [11]. The extrinsic pathway involves cell-death receptors such as tumor necrosis factor receptor (TNFR) family and Fas receptor. Binding of ligands to these receptors recruits adaptors which activate the caspases cascade [12]. Alternatively, apoptosis can be activated through intrinsic stimuli. One of the critical events in this type of apoptosis is the mitochondrial outer membrane permeabilization (MOMP) and cytochrome c release from the mitochondrial intermembrane space to the cytoplasm which triggers the assembly of apoptosome and caspases activation [13]. This pathway can also be activated in a caspase independent way, where the apoptosis-inducing factor (AIF) translocates to the nucleus to promote DNA fragmentation and chromatin condensation and, eventually, cell death [14]. Many proteins, especially tumour suppressors, are involved in the regulation of these complex cellular processes. LACTB was recently shown to play an important role in gastric cancer where it was described to induce apoptosis through the regulation of the autophagy-mediated mitochondrial pathway in oxaliplatin-resistant gastric cancer cells [4]. The involvement of apoptosis was also shown in melanoma cells and tissues, where LACTB-containing nanoparticles delivered to cells promoted the expression of apoptotic and cell cycle arrest proteins, and ROS production [5].

Our work, which studies the detailed mechanisms involved in LACTB-mediated tumour suppression in breast cancer, confirmed and further extended these studies. We show, through in vitro, in vivo and 3D culturing studies, that LACTB induction activates the caspase-independent intrinsic cell death pathway characterized by the upregulation of Puma, Bim, Bad and Bax. This occurs through LACTB-mediated ROS-dependent enhancement of DNA damage. In parallel, we show that LACTB has a negative effect on the activation of cellular survival pathways. Results from this study uncovered more detailed mechanistic functioning of LACTB tumour suppressive function, the knowledge of which can be beneficial for the design of more tuned cancer treatments.

## Materials and methods

### Cell culture

MCF-7 were purchased from Lonza and modified by introduction of H-RAS V12 oncogene to form estrogen-independent MCF7ras cells. HCC1806, HS578t and HEK293T were purchased from ATCC. MCF-7 and HEK293T cells were maintained in DMEM (Biosera) supplemented with 10% fetal bovine serum (FBS, Sigma-Aldrich) with 1% penicillin/streptomycin (Gibco) at 37ºC with 5% CO_2_. HCC1806 and HS578t were cultured in RPMI (Sigma-Aldrich) supplemented with 10% FBS and 1% penicillin/streptomycin with the same conditions. HMEC were purchased from Lonza and were immortalized by introduction of H-TERT oncogene. They were maintained in a 1:1 mixture of Dulbecco′s Modified Eagle′s Medium/Nutrient Mixture F-12 Ham (Sigma-Aldrich) and MEBM (Lonza) plus MEGM™ Mammary Epithelial Cell Growth Medium BulletKit™ (Lonza).

### 3D cell culture

Mammosphere cell culture was performed as described previously [15]. The mammospheres structures were counted in an optical microscope. Single cells were obtained by trypsinization and they were used for flow cytometry experiments and western blot analysis.

### Plasmids

Lentiviral vectors were purchased from VectorBuilder. AKT transcription responsive elements (TREs; 5’ CCATATTAGG 3’) repeated in tandems of 5; the NF-κB TREs (5’ GGGACTTTCC 3’) repeated in tandem of 5; and the Wnt/β-catenin TREs (5’ CCTTTGAA 3’) repeated in tandems of 4. A minimal promoter (miniCMV, VectorBuilder) was inserted between the TREs and the sequence of Firefly Luciferase. Doxycycline inducible system was set up with two genes: one containing the reverse tetracycline-controlled transactivator (rtTA) and the other with the Tet responsive element followed by the gene of interest.

### Synthetic lentiviral transductions

HEK293T cells 40% confluent were transfected with 1 μg of pMDG, 1 μg of PR8.2 and 1.5 g of the marker-encoding transfer vector in DMEM 10% FBS without antibiotics using X-treme gene HP DNA transfection reagent (Roche). Synthetic viral particles were collected and filtered with a 0,45 μm filter.

Cells at 40% confluency were transduced with lentivirus and 1:2000 Polybrene transfection reagent (Merck) in 5 ml of complete media for 16 h. After transduction, cells were selected with the corresponding antibiotic for each plasmid for at least one week.

### Cell proliferation assay

Cell were seeded in 96-well plates, 1000 per well in 90 µl. Once the cells were attached, cells were treated with 5 µM Z-VAD-FMK (MedChemExpress Europe) and/or with doxycycline (DOX, Sigma) at 1 g/ml and proliferation was measured using AlamarBlue (Invitrogen). After the reagent was added (10 µl), plates were left 4 h at 37ºC, 5% CO_2_ in the dark. The absorbance was measured using a Tecan plate reader (Schoeller) at 570 nm using 600 nm as a reference wavelength. After the measurement, the wells were washed using complete media and the treatment was continued.

For the experiments with N-acetyl-L-cysteine (NAC, Sigma), cells-inducing LACTB were treated with 300 µM of NAC for 6 days and cells were counted with EVE Automatic cell counter (NanoEntek).

### Western blot analysis

Cells were treated with doxycycline at 1 g/ml, with 15 nM Actinomycin D (ActD, Invitrogen) for the indicated times in each case. Total protein from MCF7ras, HCC1806 or HS578t were obtained using Pierce RIPA buffer reagent (ThermoFisher), including a cocktail of proteases inhibitors and phosphatases inhibitors (Roche). 20 µg of protein were loaded into SDS-PAGE gels with a gradient from 4 to 15% (Bio-Rad). Proteins were transblotted in a wet-transfer system (Bio-Rad) to PVDF membranes (Immobilon-P, Millipore). Next, membranes were blocked with 5% non-fat milk in TBS and 0,01% Tween-20 (v/v). Membranes were blotted with antibodies against Bim, Bax, Bcl2, Bad, Bid, Puma, PARP, cleaved PARP, caspase 7, cleaved caspase 7, caspase 9, cleaved caspase 9, EGFR, HER2, HER3, AKT, pAKT 473, p42 p44 MAPK, pMEK1/2, LRP6, P-LRP6, Wnt5 a/b, Axin, NF-κB, pNF-κB, IKKα, IKKβ, IκBα, GAPDH (1:1000 from Cell Signaling) and LACTB and Caspase 8 (1:1000, ProteinTech). Signal was detected with enhanced chemiluminescence (ECL) using Azure c600 Western blot Imaging system.

### In vivo mouse experiments

All manipulations with cells and mice were performed in Class II Biological Safety Cabinets to achieve aseptic working environment during the whole study. Cells were cultivated in media (RPMI or DMEM) + 10% FBS + 1% Penicillin-streptomycin and suspended in 50% Matrigel prior to injections. Adult female mice strain NSG (NOD.Cg-Prkdcscid Il2rgtm1Wjl/SzJ) were obtained from The Jackson Laboratory. The injected number of cells was 3 × 10^5^ for MCF7ras cells and 1,5 × 10^5^ for HCC1806 cells prepared in 15 µL for each dose applied into mammary fat pad bilaterally. Body condition score and tumor size were measured twice weekly. The DOX treatment was administered through drinking water containing 2 mg of DOX, 5 mg of glucose and ½ of tablet of Steviol (19 mg of erythritol, 10,5 mg of steviol) in 100 ml of water when tumors reached approximately 4–5 mm in diameter. The mice were euthanized when the tumors reached cumulative size of 1,5 cm or earlier. Sample sizes were chosen to reach statistical significance, and tumour measurements and data analysis were performed in a blinded fashion. The animal experiment was approved by the Animal Research Ethics Committee of Czech Academy of Science approval ID: AVCR 5282/2021 SOV II.

### Protein array

Cell lysates were obtained from MCF7ras cells after overexpressing LACTB for the indicated time points. Protein microarray was performed by RayBiotech company for human L1000 array. Proteins were considered upregulated or downregulated when they showed more than a 2-fold change. Results were analyzed considering the pathways and the biological process in which they are involved using an enrichment analysis with EnrichR and Appyiters. Results were plotted using GraphPad Prism 8.0 software.

### Reporters assay

Cells containing the reporters described above were treated with doxycycline at different time points. After the treatment, 600.000 cells were collected for all the conditions. To detect the levels of luciferase in the cells, the kit Luciferase Assay System (Promega) was used. Cells were lysate with 90 ul of Luciferase Lysis buffer 1X. Luminescence was measured for 10 s in white flat 96-well plates (ThermoFisher) using a Tecan plate reader.

### Flow cytometry analysis for apoptosis and senescence

Cells were harvested at different time points after LACTB induction with doxycycline. For analysis of apoptosis, 1 × 10^6^ cells were stained with Annexin V-Alexa Fluor 488 and propidium iodide (PI) for 15 min in dark, using the Apoptotic cell assay kit (ThermoFisher) following the instructions provided. The signal of 20,000 events was analyzed. Determination of apoptosis was measured using an LSR Fortessa cell analyzer (BD Biosciences) at the IOCB flow cytometry facility, and data was analyzed using FlowJo 10.7.1 software. Data was plotted using GraphPad 8.0 software.

Senescence was analyzed using the CellEvent™ Senescence Green Flow Cytometry Assay Kit (ThermoFisher). β-galactosidase activity was measured by flow cytometry and analyzed as described above.

### Flow cytometry analysis for ROS

Cells overexpressing or not LACTB were collected and treated with 2.5 mmol/L DCFDA (Abcam) for 30 min at 37ºC. General presence of ROS was then analyzed by flow cytometry and data was analyzed using FlowJo 10.7.1 software.

Presence of superoxide radical, was tested using MitoSOX (ThermoFisher). Briefly, cells were incubated with 5 µM of MitoSOX in media without FBS for 30 min at 37ºC in dark. Cells were collected and analyzed by flow cytometry.

In order to analyze DNA damage produced by ROS presence, cells were collected and fixed with 4% PFA for 10 min and then permeabilized with 0,1% Triton X-100 for 30 min at room temperature. Cells were incubated with 8-OHdG hydroxyguanosine antibody for 1 h (1:250, ThermoFisher) followed by incubation with Alexa Fluor 647 for 1 h (1:1000, ThermoFisher). DNA damage by presence of ROS was analyzed by flow cytometry as described above.

### EdU staining

LACTB was overexpressed for different time points and cells were collected. Briefly, cells were treated with EdU 20 µM for 90 min at the end of LACTB induction. 600.000 cells were collected for all the conditions. EdU labeling was performed for 30 min using 2 mM CuSO_4_, 8 µM CY-5 (Sigma) and ascorbic acid 20 mg/ml. Cells were also stained with DAPI at 500 ng/ml for 15 min to stain the DNA. Signal was detected by flow cytometry and data analyzed using FlowJo 10.7.1 software.

### Immunofluorescence in cells and tissues

Cell lines were seeded on cover slips after the LACTB induction. Mitochondria were stained using Mitotracker Deep Red (Invitrogen) at 200 nM in media without FBS for 45 min at 37ºC in dark. Cells were fixed with paraformaldehyde 4% for 30 min, they were permeabilized with Triton X-100 0,1% in PBS for 10 min and blocked with BSA 1% in PBS for 20 min. Cells were incubated with γ-H2AX (1:400, Cell Signaling), ki-67 (1:250, BD Pharmingen), LACTB (1:250, ProteinTech), AIF (1:100, ProteinTech) or BAX 6A7 (1:50, eBioscience) followed by the secondary antibodies coupled with Alexa Fluor indicated for each experiment and each protein (1:400, ThermoFisher) and DAPI at 500 ng/ml for 15 min to stain nucleus. Cells were observed in a Zeiss LSM 780 confocal microscope with a 63X objective. Images correspond to one channel and one section in the z axis in the middle of the cell. Images were analyzed with ZEN 3.2 viewer software and with Image J.

Tumours were embedded in paraffin for follow-up analysis. Sections of 2 μm were deparaffined by heating at 60ºC for 10 min followed by xylene incubation and antigen retrieval was achieved by boiling the samples in citrate buffer for 20 min. Samples were then permeabilized with 0,2% Triton X-100 for 15 min and blocked with 30% horse serum for 30 min. Sections were incubated with primary antibodies overnight at 4ºC and with secondary antibodies (AlexaFluor, ThermoFisher, 1:400) for 1 h at room temperature. Tissues were stained with DAPI at 500 ng/ml for 15 min and mounted on glass microslides in Prolong Gold antifade reagent.

### Statistical analysis

Data is shown as the average ± standard deviation (SD). Comparative studies between quantitative variables following normal distribution were valuated using Student t test for paired or no-paired samples according to the experiment. For in vivo experiments differences in tumour growth were analyzed by 2-way ANOVA. Differences were considered significant when p value ≤ 0,05. Statistical analysis was performed using GraphPad Prism 8 software.

## Results

### LACTB induces cell growth arrest in breast cancer cells

**Fig. 1:**
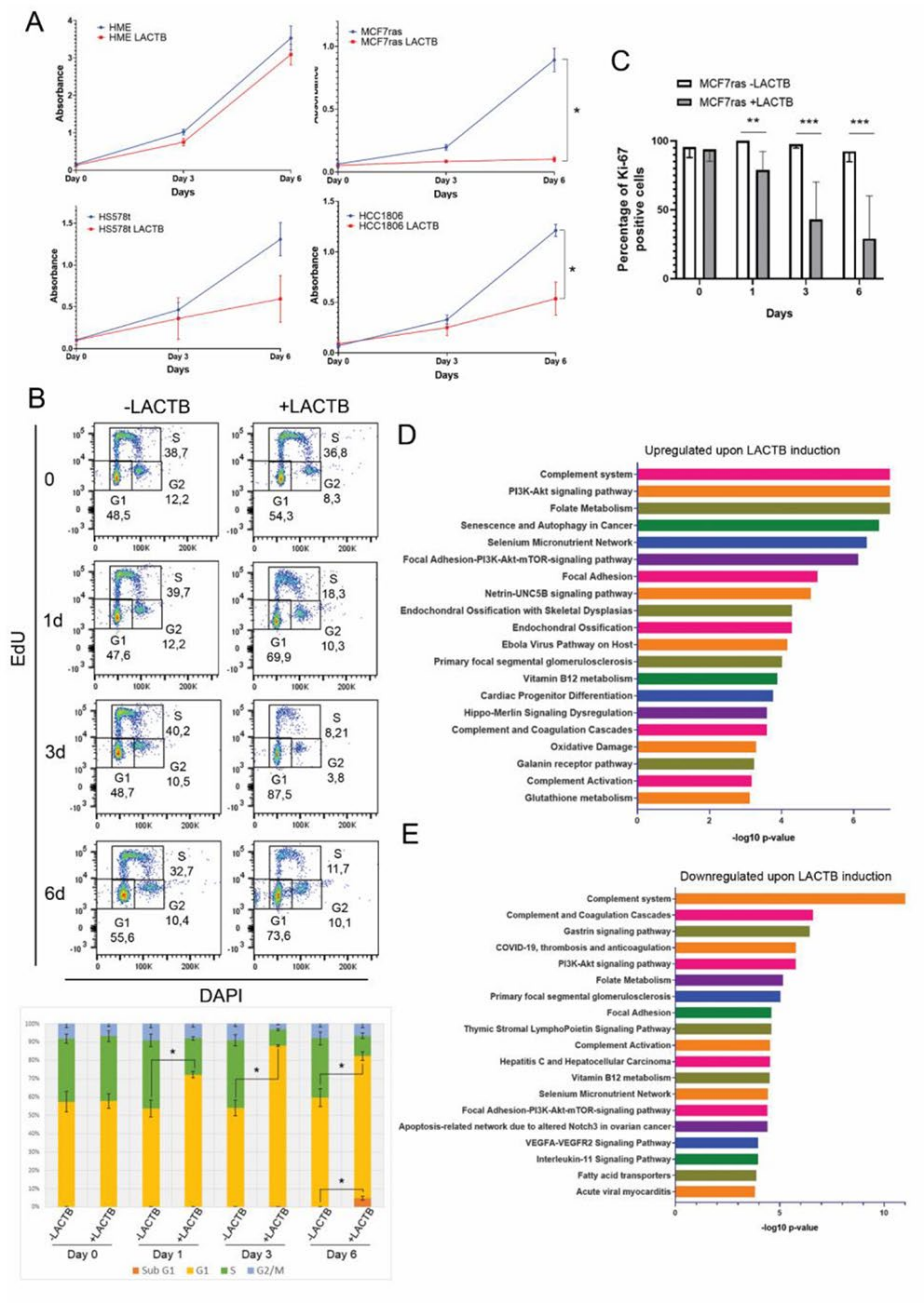
***LACTB expression inhibits cancer cell growth.*** (A) Cell proliferation assay in HME, MCF7ras, HCC1806 and HS578t upon LACTB induction. * P < 0.05 vs. control group. (B) FACS-based cell cycle analysis of EdU/DAPI in MCF7ras cells. In the upper part, dot plots from one representative experiment are shown. In the lower part, averages from each phase of the cycle of three independent experiments are shown. * P < 0.05 vs. control group (cells not overexpressing LACTB for each time point). (C) Statistical analysis of the immunofluorescence signal for Ki-67 in MCF7ras cells. Positive cells for Ki-67 were counted and average ± SD is represented. **P < 0,01; ***P < 0,001. (D and E) Enrichment analysis performed in EnrichR and Appyiters from the protein array. Proteins 2-fold upregulated (upper graph, D) or 2-fold downregulated (lower graph, E) upon LACTB induction were considered. The 20 most significative pathways are plotted

In order to uncover the mechanism of LACTB’s tumor suppressive effects we induced doxycycline inducible LACTB in a panel of normal and breast cancer cell lines and monitored their growth for 6 days. The growth rate of all breast cancer cell lines tested was negatively affected by the induction of LACTB expression while no significant changes were observed in the control non-tumorigenic HME cell line after LACTB induction (Fig. 1 A). We confirmed these observations by FACS analysis examining the cell cycle progression of breast cancer cells with and without LACTB expression at different time points. The FACS analysis of MCF7ras cells showed a significant increase of cells in G1 phase and decrease in S phase upon LACTB induction (Fig. 1B). Modest increase was also observed in the Sub-G1 population in later time points of LACTB induction (day 6), which indicates cell death. G1 cell cycle arrest was also observed in two of the other breast cancer cell lines tested (HCC1806 and HS578t, Suppl. Figure 1 A, B). These experiments were further validated by fluorescence confocal microscopy, which showed a significant decrease of the proliferation marker Ki-67 in cells where LACTB was induced, starting from day 1 of LACTB induction (Fig. 1 C and Suppl Fig. 1 C). Since G1 cell cycle arrest can drive the cells to different fates, we tested whether LACTB was inducing senescence in MCF7ras cells. However, no differences were observed in the appearance of the β-galactosidase senescent marker in cells with and without LACTB induction (Suppl. Figure 1D). We then employed the antibody array screen, which profiles the expression levels and activation of numerous signaling pathway proteins, to identify factors that play part in the cell growth arrest induced by LACTB expression. In this assay, we examined the protein cell lysate from MCF7ras breast cancer cells with different time points of LACTB induction (6 h, 1 day, 3 days, 6 days). We focused our attention on proteins whose expression levels changed at least 2-fold upon LACTB expression. Pathways and cell processes that are connected with cell death and apoptosis, such as autophagy, oxidative damage and AKT signaling pathway were upregulated (Fig. 1D) whereas processes related with cell survival such as focal adhesion were downregulated (Fig. 1E). We therefore decided to research in more detail the role of apoptosis, oxidative damage and cell survival in the mechanism of LACTB-mediated tumor suppression.

### LACTB induction leads to activation of the intrinsic apoptotic pathway

**Fig. 2:**
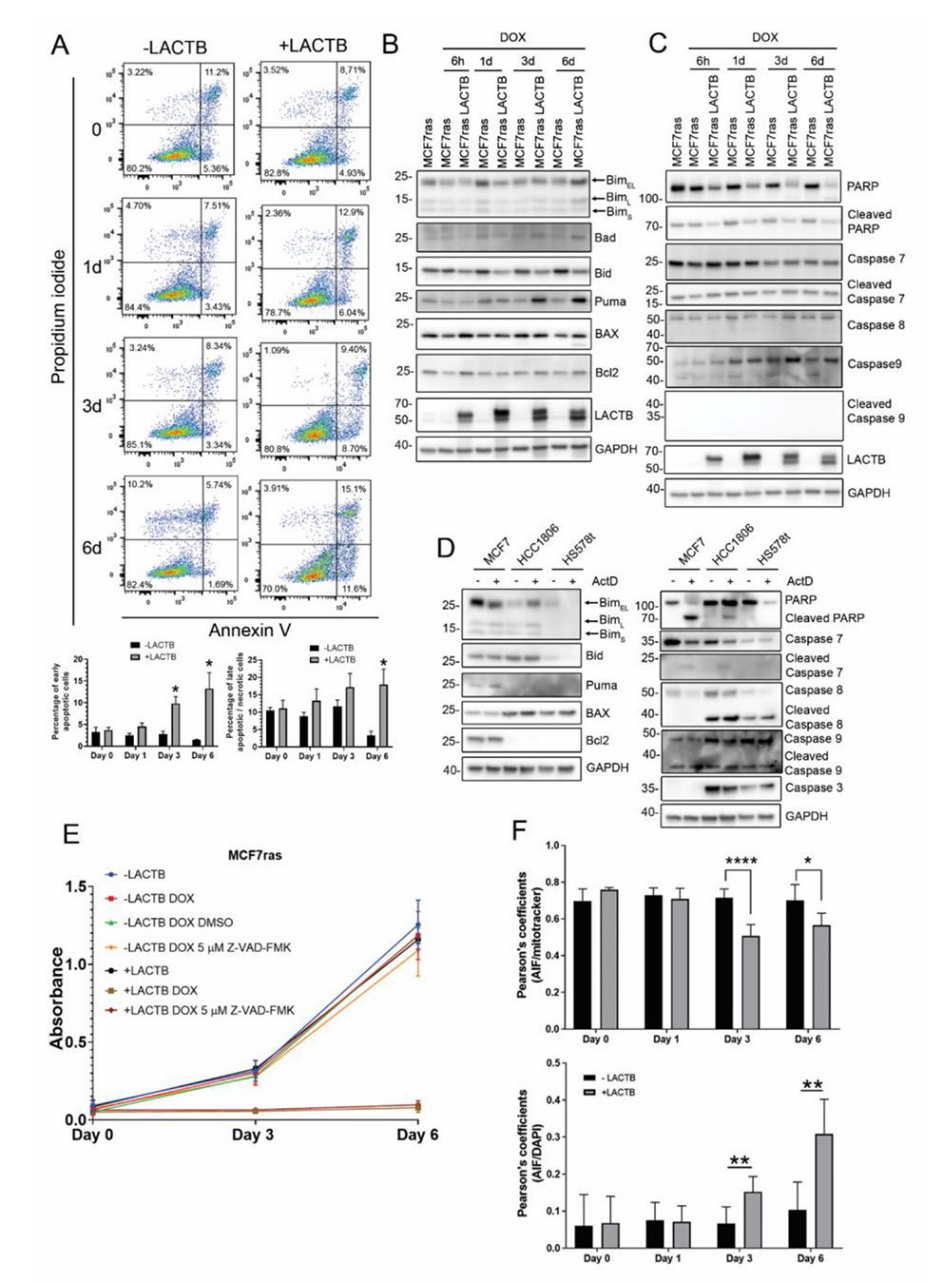
***LACTB induced the intrinsic pathway of apoptosis in breast cancer cell lines.*** (A) Annexin V/PI analysis by flow cytometry of MCF7ras cells. LACTB was induced for the indicated time points and percentage of early apoptotic cells and late apoptotic/necrotic cells were measured by flow cytometry. Values of apoptotic ratios for each condition are presented as the mean ± standard deviation. * P < 0.05 vs. control group (-LACTB). (B) Western blot analysis of the apoptotic intrinsic pathway and the caspases levels (C) in MCF7ras overexpressing LACTB for the time points that are indicated. (D) MCF7ras, HCC1806 and HS578t cells were treated with 15 nM of actinomycin D for 24 h and protein levels were analyzed by western blot. (E) Cell proliferation assay in MCF7ras and MCF7ras where LACTB was induced. A pan-caspase inhibitor was used (5 µM Z-VAD-FMK) and proliferation was measured with AlamarBlue. (F) Pearson’s coefficient between AIF and Mitotracker (upper graph) and between AIF and DAPI (lower graph). Pearson’s coefficient was calculated with JACoP plugin in Image J. Values are represented as the mean ± standard deviation. *P < 0,05; **P < 0,01; ****P < 0,0001

In order to confirm the involvement of apoptosis in LACTB mechanism we examined the expression of Annexin V apoptotic marker and propidium iodide DNA marker by FACS analysis. An increase in Annexin V staining was observed over time in MCF7ras, HCC1806 and HS578t cells, indicating the onset of apoptotic processes in these cell lines (Fig. 2 A and Suppl. Figure 2 A and 2B). In order to validate the results from the protein array and to find out which proteins play an active role in the LACTB-mediated apoptotic cell death, we analyzed the expression of these proteins by western blot. Analysis of different apoptotic proteins from the intrinsic and the extrinsic pathways showed that in MCF7ras cells LACTB predominantly induced the upregulation of Puma and Bad (Fig. 2B). Interestingly, downregulation of Bid, which is associated with the enhancement of the intrinsic pathway of apoptosis, was also present [16]. Poly (ADP-ribose) polymerase (PARP), a protein involved in DNA repair, was also decreased upon LACTB induction. However, this downregulation was not due to protein cleavage (Fig. 2 C). Levels of caspases showed no change after LACTB overexpression, indicating that LACTB-mediated apoptosis in this cellular model could be a caspase-independent process. This might be explained by the fact that MCF7ras cells lack the caspase 3 gene [17–19]. These results were confirmed using an Actinomycin D, an established apoptosis inducer, as a positive control [20]. In contrast to LACTB induction, the treatment of a panel of breast cancer cell lines (MCF7ras, HCC1806, Hs578t) with Actinomycin D resulted in PARP degradation and caspase cleavage in all or some of our tested cell lines (caspase 7, 8 9) confirming the onset of caspase-dependent apoptosis (Fig. 2D). As expected, the levels of Annexin V were also strongly increased upon Actinomycin D treatment (Suppl. Figure 2 C). A pan-caspase inhibitor (Z-VAD-FMK) was used to further examine the role of the caspases in LACTB mechanism. No rescue of the MCF7ras cells was observed after LACTB induction in the presence of the inhibitor confirming the activation of the caspase-independent cell death pathway (Fig. 2E). Next, we were interested to know whether similar pattern is also observed in other breast cancer cell lines. Indeed, HS578t cell line presented a similar phenotype compared with MCF7ras, Bim overexpression, Bid downregulation and lack of caspases cleavage (Suppl. Figure 2D). Interestingly, HS578t cells were also reported to have very low levels of caspase 3, compared with other breast cancer cell lines, what might explain the preferential activation of the caspase-independent processes upon LACTB induction [21]. In HCC1806 LACTB is inducing Bim and BAX expression in early time points, both involved in the pore formation in the outer membrane of the mitochondria [22, 23], supporting the result from the Annexin V/PI staining at day 1 of LACTB induction (Suppl. Figure 2E). As with previous cell lines, no changes in caspase levels were observed in HCC1806 cells, even though this cell line has no defect in any studied caspases (Suppl. Figure 2E). This suggests that in breast cancer LACTB predominantly acts through caspase-independent cell death mechanisms. To confirm that LACTB was inducing the intrinsic pathway in MCF7ras cells we used confocal microscopy to observe the release of the apoptosis inducible factor (AIF) from the mitochondria (Suppl. Figure 3 A). Results showed a decrease and a more diffuse signal of AIF in mitochondria at day 6 of LACTB expression. Furthermore, mitochondria are positioned closer to the nucleus and in some cells, AIF is localized inside the nucleus. It is interesting to point out that in some cases we also observed colocalization of LACTB and AIF in the nucleus (Suppl. Figure 3B). The Pearson’s coefficient between Mitotracker and AIF signal was significantly reduced from day three, indicating that AIF is released from the mitochondria (Fig. 2 F). AIF release requires the permeabilization of the mitochondria. Considering that one of the plausible mechanisms for the permeabilization of the mitochondria is the BAX pore formation, we investigated, by immunofluorescence analysis, whether there is an increase of active BAX in the mitochondria upon LACTB induction. However, we did not detect any increase in the levels of active BAX upon LACTB induction (for 1 day and 6 days) suggesting that the AIF release is mediated by BAX-independent mechanisms and might be dependent on the mitochondrial permeability transition pore (Suppl. Figure 3 C). Taken together, these results showed that LACTB induction leads to activation of the caspase-independent intrinsic cell death pathway in several tested cellular models. However, the specific factors that play a role in this process differ based on the genetic background of the tested cell lines.

### LACTB induces ROS and double strand DNA breaks

**Fig. 3:**
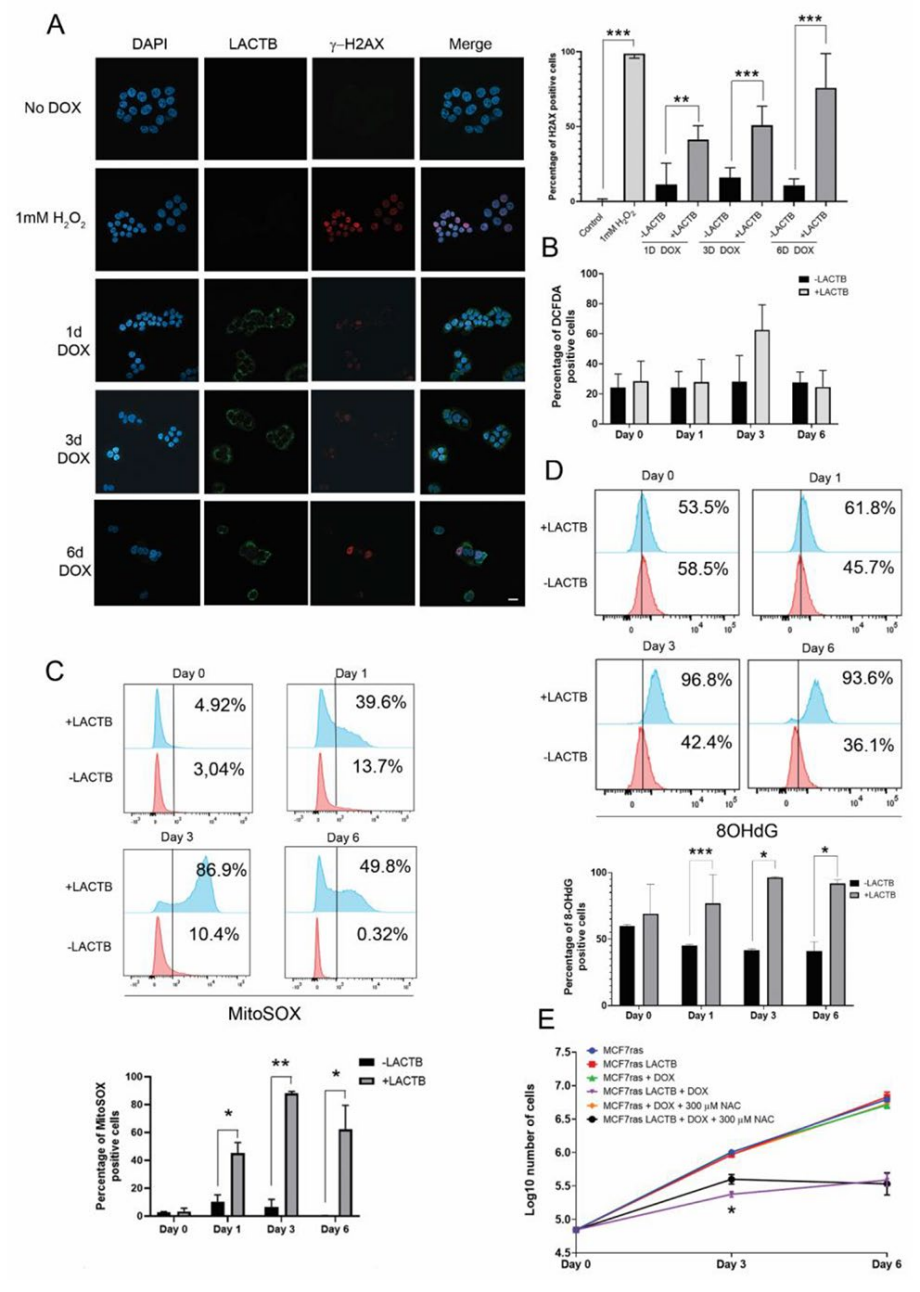
**Analysis of ROS and DNA damage upon LACTB induction.** (A) Immunofluorescence for γ-H2AX in MCF7ras. Images were taken with a Zeiss LSM 780 confocal microscope from a middle section of the cells. γ-H2AX-positive cells were counted, and averages ± SD from different experiments are shown on the right graph. (B) Flow cytometry analysis of general ROS production after LACTB induction. (C) Flow cytometry analysis of mitochondrial ROS superoxide by MitoSOX. (D) Flow cytometry analysis of ROS-mediated DNA damage by 8-OHdG detection. (E) Cell proliferation assay with the antioxidant NAC and LACTB overexpression. Values are represented as the average ± SD of three independent experiments. *P < 0,05; **P < 0,01; ***P < 0,001. Scale bar: 10 μm

DNA damage is known to function as a signal for cell cycle arrest and cell death. Since PARP is downregulated upon LACTB expression in MCF7ras and HS578t, we decided to investigate whether LACTB-expressing cells have signs of DNA damage. Using confocal microscopy, we examined the levels of DNA damage using the yH2AX marker, which binds double strand DNA breaks. Results showed a progressive increase in the DNA damage up to 6 days of LACTB overexpression in MCF7ras cells. Treatment of cells with H_2_O_2_ was used as a positive control for the presence of DNA damage (Fig. 3 A). Since the presence of ROS within cells is one of the main causative agents of the appearance of DNA damage we examined if cancer cells had an increase in ROS production upon LACTB induction. ROS production was tested by flow cytometry using DCFDA (for general ROS presence in the cell), MitoSOX (for superoxide production, mainly mitochondrial), and 8-OHdG antibody (for specific ROS-mediated DNA damage). Results showed no statistical difference regarding the general presence of ROS, (Fig. 3B), and an increase of mitochondrial superoxide radical from early time points of LACTB induction (Fig. 3 C). The increase in mitochondrial ROS production upon LACTB induction are in agreements with the reports by Yang et al. 2021 and by Liu & Wu, 2021, which showed the effect of LACTB on ROS in gastric cancer and melanoma, respectively. Furthermore, we observed, using 8-OHdG antibody that recognize oxidation of guanidine nucleotides by superoxide radical, that the ROS-mediated DNA damage was observable from day 3 of LACTB induction (Fig. 3D). In order to find out whether induction of ROS in our panel of breast cancer cell lines is the major mode of LACTB induced cell death we used antioxidants to prevent the DNA damage. N-acetyl-L-cysteine (NAC) is a commonly used antioxidant to avoid ROS-dependent negative effects in cells. MCF7ras cells were treated with doxycycline to induce LACTB for 6 days with or without the addition of 300 µM NAC. Indeed, antioxidant treatment partially rescued the cells at early time points (day 3) but not in later time points (day 6) of LACTB induction (Fig. 3E). This result indicates that LACTB induced cancer cell death is partially dependent on ROS formation/DNA damage in early time points of its expression while the remaining cell cycle arrest is ROS-independent and may act through AIF re-localization in the nucleus.

### LACTB negatively affects pathways involved in cell survival

**Fig. 4:**
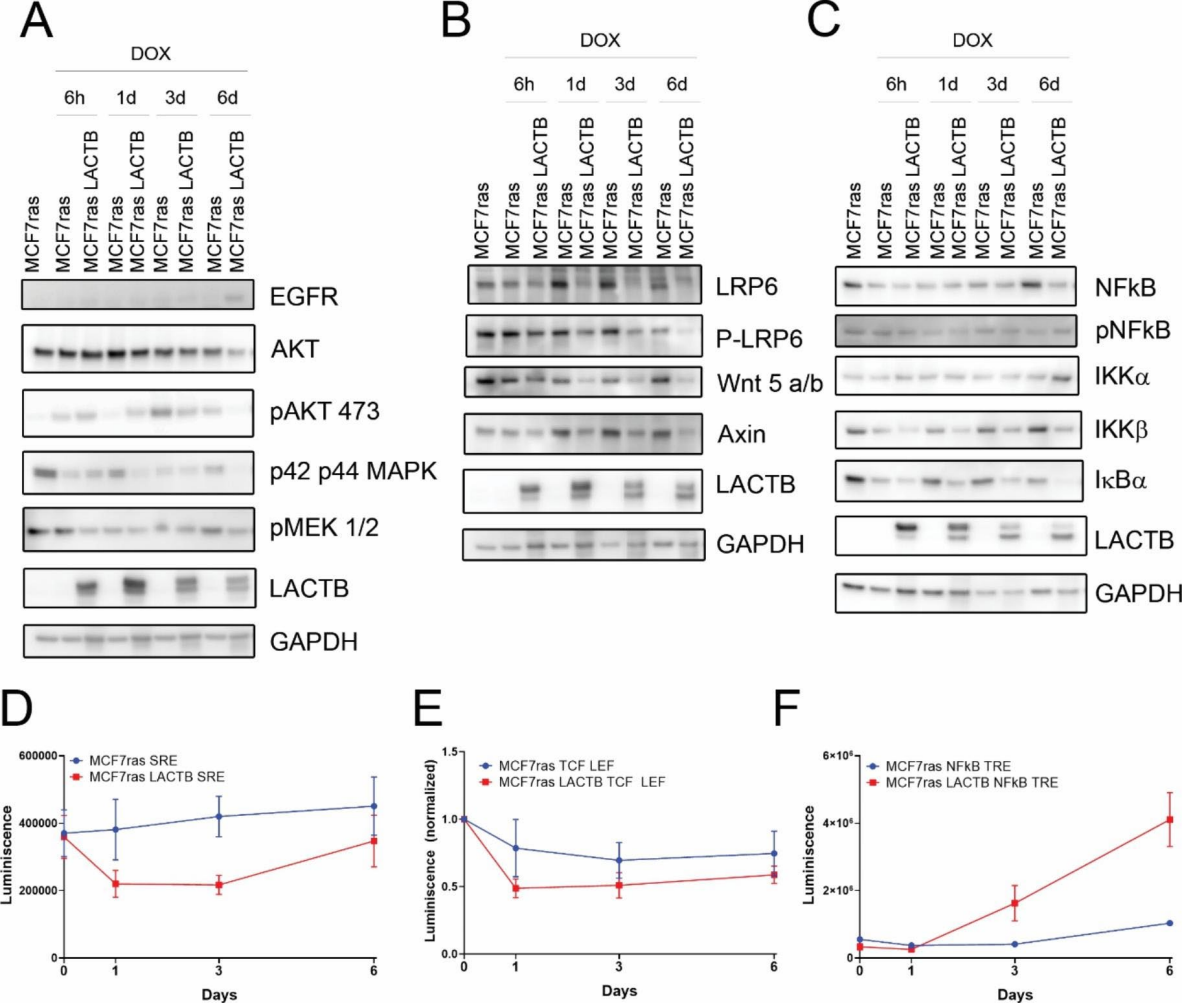
**Pro-survival pathways are modulated upon LACTB induction.** Analysis of different signaling pathways of MCF7ras cells overexpressing LACTB for the indicated time points. EGFR-PI3K-MAPK (A), Wnt/β-catenin (B) and NF-κB (C) signaling pathways were analyzed. (D) Reporter assay for AKT pathway, (E) for the Wnt/β-catenin signaling pathway (F) and for NF-κB pathway. Experiments were repeated 3 times and average ± SD is plotted in the graphs

Many signaling pathways, such as PI3K/AKT/mTOR, Wnt/β-catenin and NF-κB, have been described to promote cell survival, proliferation and tumorigenesis in cancer cells [24–28]. We were interested to examine whether LACTB induction modulates the activity of any of these pathways. The western blot analysis of MCF7ras cells where LACTB was induced for 6 days showed that LACTB induction led to downregulation of AKT pathway, as shown by a decrease in phosphorylation of MEK 1/2, AKT, p42 and p44 (Fig. 4 A). Furthermore, LACTB induction also decreased the activation of Wnt/β-catenin signaling pathway, with a decrease in the receptor LRP6 and its phosphorylated form and the ligand Wnt5 a/b (Fig. 4B). We next employed luciferase reporter experiments, to test directly the effect of LACTB expression on the activity of these pathways. The results of these experiments were in agreement with our western blot analysis and antibody array results showing a significant downregulation of the AKT pathway (Fig. 4D) and modest downregulation of Wnt/β-catenin pathway (Fig. 4E). NF-κB is phosphorylated in later time points after LACTB overexpression. Moreover, the inhibitor of the pathway, IκBα, is downregulated. This correlates with an increase in the luciferase activity in the reporter assay (Fig. 4 C and Fig. 4 F). This might be explained by the fact, that caspase independent cell death is able to induce NF-κB signaling pathway and release cytokines and chemokines to the extracellular media to induce an inflammatory response [29]. Similar results were also observed in other tested breast cancer cell lines, HCC1806 and Hs578t. The levels of phosphorylation in NF-κB were high at 6 days of LACTB induction in HCC1806, and at day 3 in HS578t. Downregulation of Wnt/β-catenin was observed at day 6 of LACTB induction in both cell lines as a decrease in the receptor LRP6 and ligand Wnt 5 a/b (Suppl. Figure 4 A and B). These results confirmed that LACTB induction leads to inhibition of pro-survival cellular pathways in various cell line models.

### LACTB expression induces cell death under 3D and conditions

**Fig. 5:**
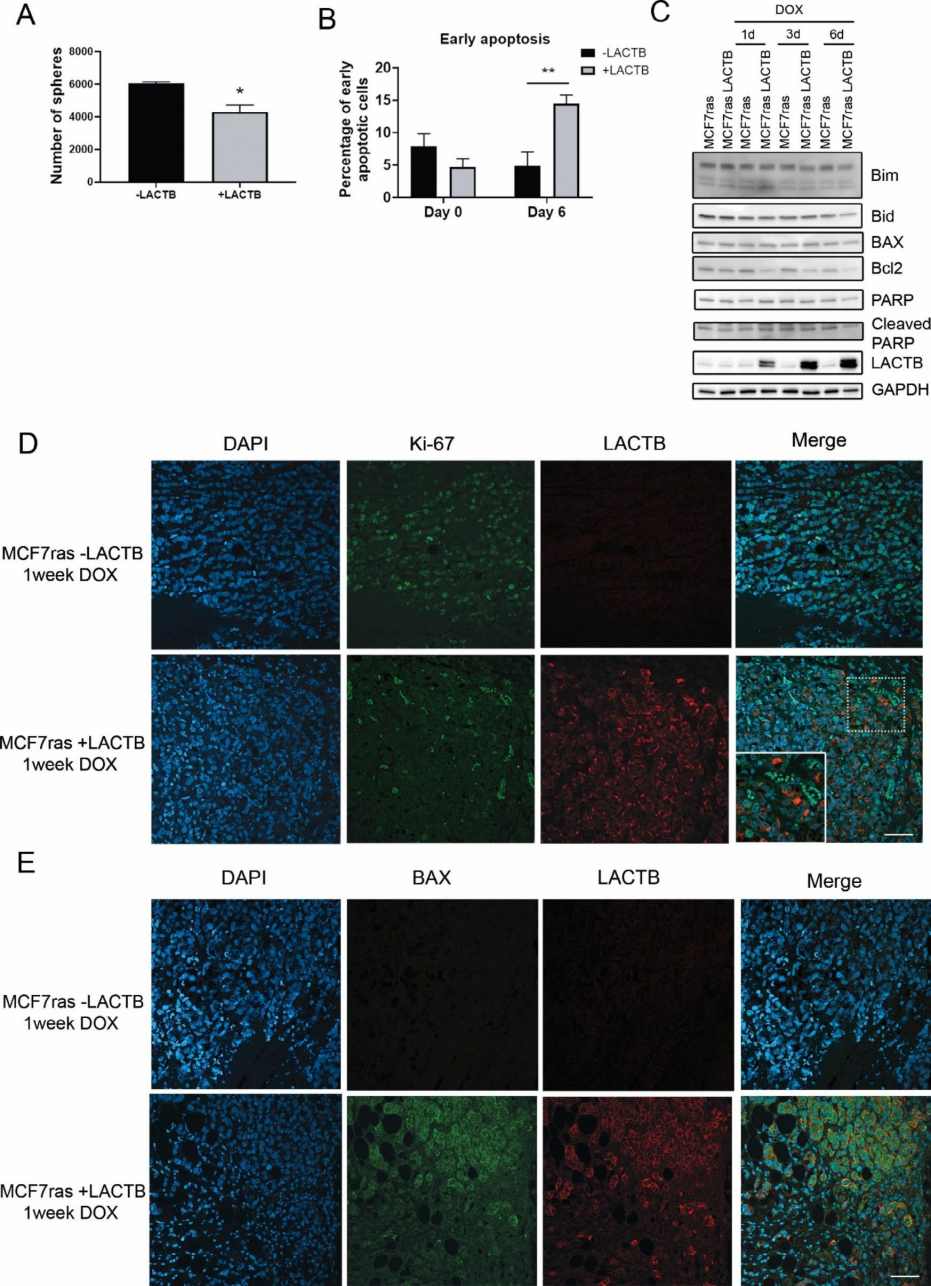
***The effect of LACTB induction under 3D and in vivo conditions.*** (A) Number of spheres in control MCF7ras cells and MCF7ras where LACTB was induced for 6 days. Average ± SD of three experiments are plotted in the graph. (B) FACS analysis of Annexin V staining in spheres with and without LACTB induction. Early apoptosis was considered. (C) Western blot analysis of apoptotic pathways in MCF7ras spheres after LACTB induction for the indicated time points. (D and E) Immunofluorescence analysis of tissue sections of control (MCF7ras -LACTB) and cells overexpressing LACTB (MCF7ras + LACTB) after one week of treatment with DOX Samples were stained with Ki-67 and BAX (E) to examine the proliferation and cell death, respectively. Scale bar: 50 μm. *P < 0.05; **P < 0,01 vs. cells not expressing LACTB.

In order to have a better perspective of LACTB-mediated apoptosis in breast cancer, we examined whether LACTB induction under a more physiological 3D culturing conditions leads to changes in cellular signaling similar to that of 2D cultivation. Therefore, MCF7ras cells were cultivated for different time points of LACTB induction under 3D culturing condition. As expected, LACTB induction in MCF7ras cells significantly decreased the number of spheres (Fig. 5 A and Suppl. Figure 5 A). At the termination of the experiment, spheres were collected, dissociated and single cells were analyzed by flow cytometry using Annexin V assay. Similarly to 2D conditions, cultivation under 3D conditions also led to an increase of apoptotic cells after LACTB induction (Fig. 5B). Additionally, as shown in 2D conditions, LACTB overexpression under 3D conditions downregulates Bid, Bcl2, Bim and PARP at protein levels (Fig. 5 C). Furthermore, we also observed downregulation in p42 p44 MAPK levels from day 3 of LACTB induction, slight increase of phosphorylation of NF-κB at day 6 and decrease of IκBα from day 3 (Suppl. Figure 5B). Next, we determined if the expression of LACTB in formed tumors was also enhancing cell death. MCF7ras cells transduced with doxycycline inducible LACTB were injected in mice and the size of the tumors was analyzed over time after induction of LACTB with doxycycline. A significant decrease of the tumour growth was observed after 3 weeks of DOX treatment (Suppl. Figure 5 C). Immunofluorescence in tumor tissues showed a negative correlation of Ki-67 and LACTB, indicating that LACTB is decreasing cell proliferation under in vivo conditions (Fig. 5D). Moreover, a positive correlation of the pro-apoptotic protein BAX and LACTB was found in these tissues (Fig. 5E). These results were also confirmed in tissues from HCC1806 tumors where LACTB was expressed for 2 weeks (Suppl. Figure 5D and E) further confirming the ability of LACTB to induce cancer cell death through the enhancement of the intrinsic apoptotic pathway also under 3D and in vivo conditions.

## Discussion

Tumour suppression is a complex process in which various proteins modulate different signaling pathways to promote cell cycle arrest, differentiation, senescence, or apoptosis. The specific outcome of these types of tumor suppressive mechanisms is highly dependent on particular cell lines and tissues, which display different phenotypes based on the cell heterogeneity and genetic backgrounds. Such an example can be found in a wide variety of tumour suppressors, one example being LACTB protein. In this regard, LACTB was shown to mediate tumour suppression through PI3K/AKT/mTOR in colorectal cancer [7], inhibiting Hippo pathway in melanoma [8], modulating lipid metabolism in breast cancer and hepatocellular carcinoma [2, 6] and regulating autophagy in gastric cancer [4].

**Fig. 6:**
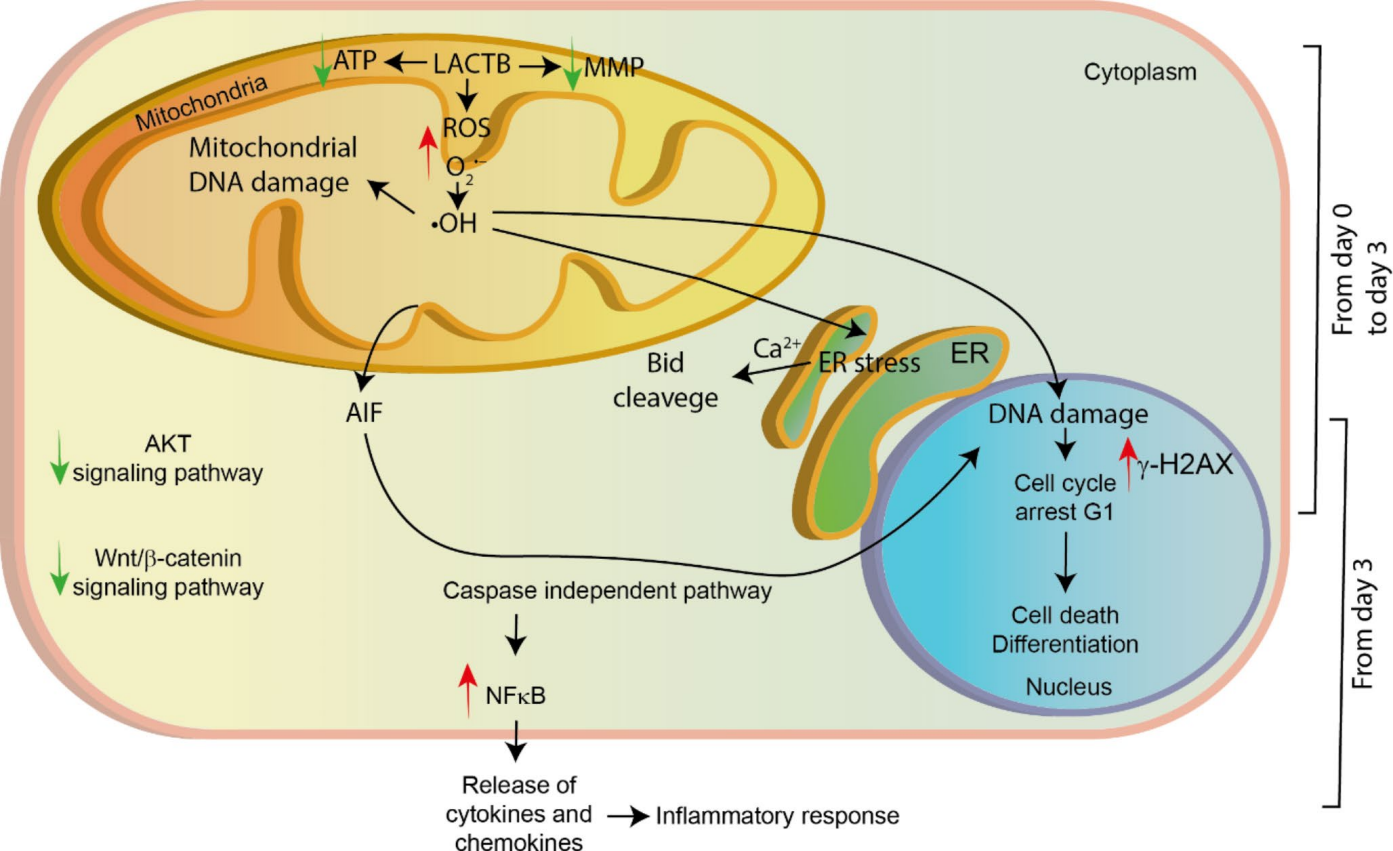
***Schematic depiction of the general LACTB mechanism in breast cancer.*** LACTB induces the production of mitochondrial ROS and a decrease of ATP levels and the mitochondrial membrane potential (MMP) as was previously shown [2]. ROS can lead to DNA damage, mitochondrial dysfunction and/or ER stress. Further cell fate is dependent on the levels of ROS in the cells, the amount of DNA damage and the existing antioxidants mechanisms. Most of the cells will go under cell cycle arrest in G1. Some cells will enter the apoptosis process by the release of AIF from the mitochondria. Cells that are able to survive and re-start their growth develop a more differentiated phenotype, as was previously shown [2]

Mitochondria control many important cellular processes, such as oxidative phosphorylation, lipid metabolism, redox balance, cell survival and apoptosis. Several mitochondrial tumour suppressors were recently described modulating these processes to oppose tumorigenesis and some therapies have been developed based on this knowledge [30]. Herein, we described the more detailed mechanism by which LACTB suppresses tumorigenesis and induces cancer cell death in breast cancer (Fig. 6). This process is divided into two general mechanisms: First, occurring at early time points in which ROS and cell cycle arrest are involved, and second, occurring at later time points in which the caspase-independent intrinsic pathway of the cell death process takes place. ROS production is described to occur in cancer cells under continued stress [31–33]. High levels of these agents can reach the DNA and produce breaks that will initiate the repair machinery [34, 35]. However, we show, that upon LACTB induction, this repair machinery is downregulated, inducing cell cycle arrest in G1 due to high DNA damage. This process can be partially reverted through using treatment with antioxidants suggesting that cell cycle arrest is partly governed by other stimuli than ROS production. These results are in accordance with the results in other tissues types, that also showed the importance of ROS production in LACTB mechanism [4, 5]. Additionally, ROS is able to induce ER stress and consequently the release of Ca^2+^ [36–38]. Increase of levels of Ca^2+^ promotes Bid cleavage by calpains [39]. Activation of Bid leads BAX oligomerization [40], however, LACTB-mediated cell death is independent of BAX pore formation. This shows that the AIF release upon LACTB induction is mediated by BAX-independent mechanisms and might be dependent on the mitochondrial permeabilization by the transition pore, which can be mediated by lipids, calcium or ROS [41, 42].

Cell fate is highly dependent on the levels of DNA damage and whether the cells are able to repair it [43]. In this regard, since LACTB is inducing ROS-mediated DNA damage, portion of the cells within the population undergo cell death while the rest might stay blocked in G1 phase. This process is controlled by the release of AIF from the mitochondria to the nucleus to enhance the DNA degradation. Interestingly, we have noticed that in some cells LACTB colocalizes with AIF in the nucleus, which can be indicative LACTB’s role in the DNA degradation complex. By using a Bio-ID approach, it was previously shown that LACTB can interacts with AIF in the mitochondria [44, 45]. Future experiments might shed light on whether LACTB has a role in the cleavage of AIF in the mitochondria or in promoting the DNA degradation in the nucleus. However, the complexity of cellular answers to LACTB is manifested by the fact that other cellular models within breast cancer category are reacting differently to LACTB. This can be due to the different DNA repair mechanisms and antioxidant protein levels to avoid the cell death. Moreover, DNA damage, ROS production and cell cycle arrest are able to induce cellular terminal differentiation under certain conditions [46–49]. Combination of different cell phenotypes and genotypes can change the cell fate upon LACTB induction between cell death, cell cycle arrest and/or differentiation. Interestingly, whatever the specific selected route of action is, the outcome stays the same: the negative effect on the life of cancer cell. Several compounds that are now being tested and drugs that are used in the clinic to treat breast cancer are able to mimic LACTB tumour suppressive mechanism. Natural compounds were shown to induce ROS, cell cycle arrest in G1, activate the intrinsic pathway of apoptosis and downregulate AKT signaling pathway [50–53]. Moreover, synthetic compounds have also been tested and showed a similar mechanism in breast cancer. As an example, a triazole precursor induced generation of ROS, cell cycle arrest and upregulation of pro-apoptotic proteins in MCF7ras cells [54].

Cancer cell growth and cell survival is characterized by the activation of many signaling pathways. Among them, AKT and Wnt/β-catenin signaling pathways are characterized to promote cell growth. Interestingly, LACTB is capable of downregulating these pathways, which correlates with a decrease in the cell proliferation. This is in agreement with a study performed in colorectal cancer which showed the ability of LACTB to inhibit AKT signaling pathway [7]. Interestingly, NF-κB signaling pathway, which can be involved in cell survival or cell death, is upregulated in later time points of LACTB induction. This upregulation was already described in a caspase-independent pathway for apoptosis and leads to a NF-κB-mediated pro-inflammatory response [29]. These extracellular signals recruit the immune system to attack the tumour. Further research in this area can open new perspectives on how LACTB and the extracellular components might interact in order to inhibit tumour progression.

## Electronic supplementary material

Below is the link to the electronic supplementary material.

Supplementary Material 1

Supplementary Material 2

Supplementary Material 3

Supplementary Material 4

Supplementary Material 5

Supplementary Material 6

Supplementary Material 7

Supplementary Material 8

Supplementary Material 9

Supplementary Material 10

Supplementary Material 11

Supplementary Material 12

Supplementary Material 13

Supplementary Material 14

Supplementary Material 15

Supplementary Material 16

Supplementary Material 17

Supplementary Material 18

Supplementary Material 19

## Data Availability

The datasets used and/or analyzed during the current study are available from the corresponding author on reasonable request. All data generated or analyzed during this study are included in this published article (and its supplementary information files).

## List of abbreviations

AIF: Apoptosis-inducing factor
DOX: Doxycycline
ECL: Enhanced chemiluminescence
LACTB: Serine beta-lactamase-like protein
MOMP: Mitochondrial outer membrane permeabilization
NAC: N-acetyl-L-cystein
NF-κB: Nuclear factor κB
PI: Propidium iodide
ROS: Reactive oxygen species
rtTA: Reverse tetracycline-controlled transactivator
SD: Standard deviation
TNFR: Tumor necrosis factor receptor
TRE: Transcription responsive elements
