# Supplementary figures and images for: LACTB induces cancer cell death through the activation of the intrinsic caspase-independent pathway in breast cancer
Source: Apoptosis. 2022 Oct 25;28(1-2):186–98. doi: 10.1007/s10495-022-01775-4 (PMC9950249; doi:10.1007/s10495-022-01775-4)

**Figure 2B**

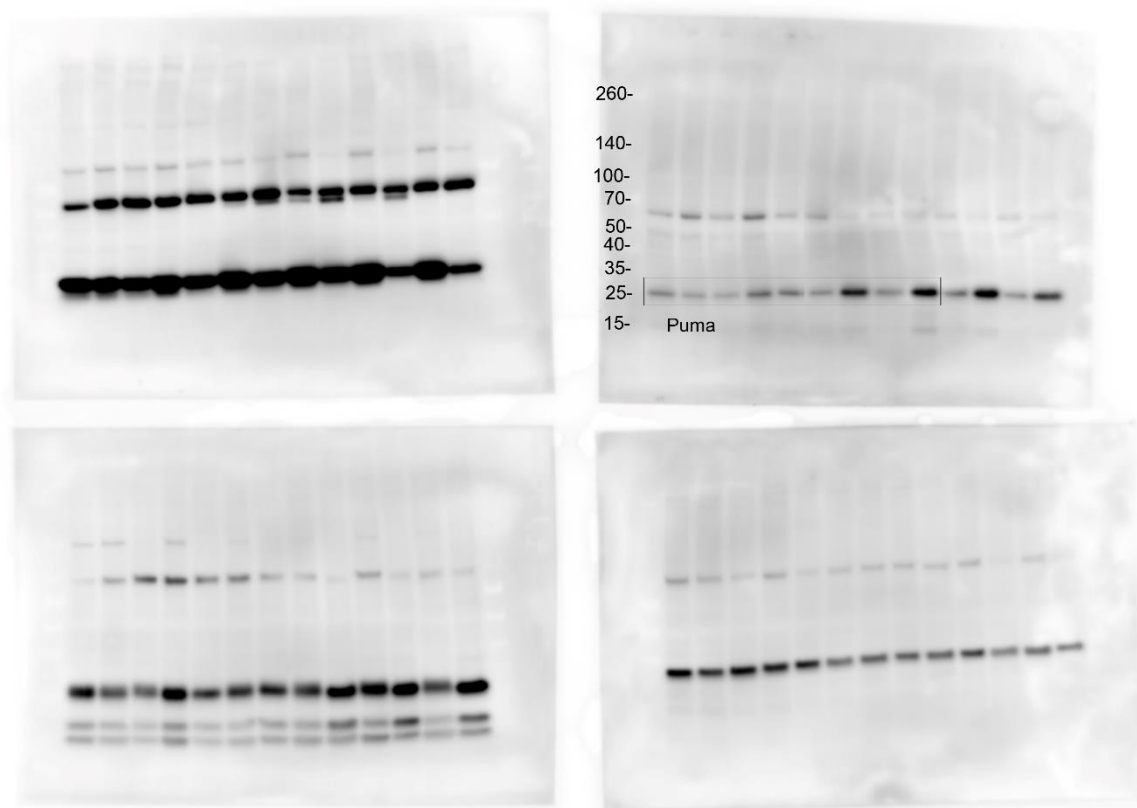

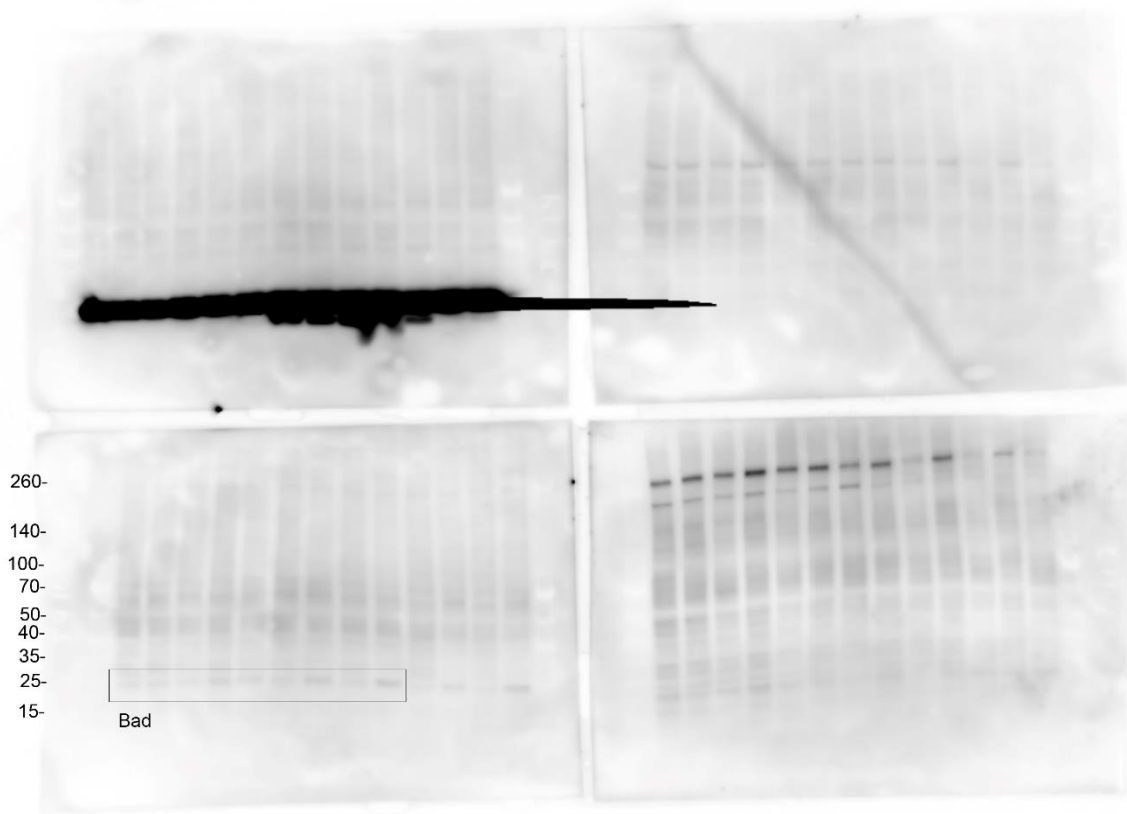

---

260-

140-

100-

70-

50-

40-

35-

25-

15-

BAX

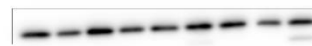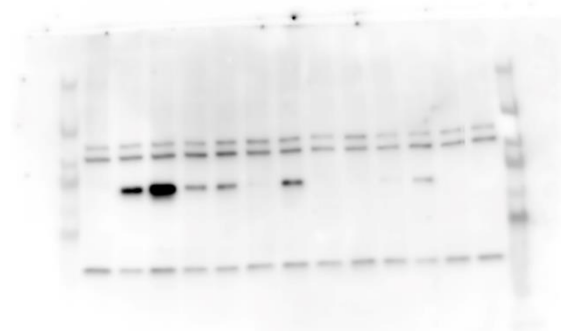

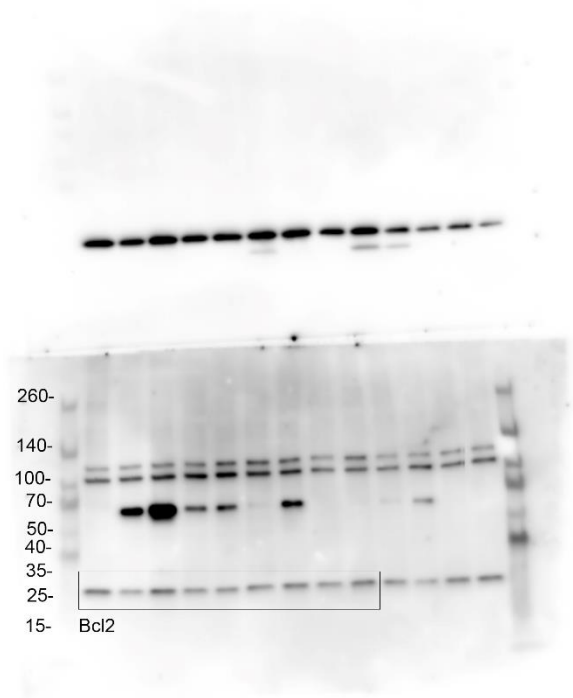

260-

140-

100-

70-

50-

40-

35-

25-

15-

Bid

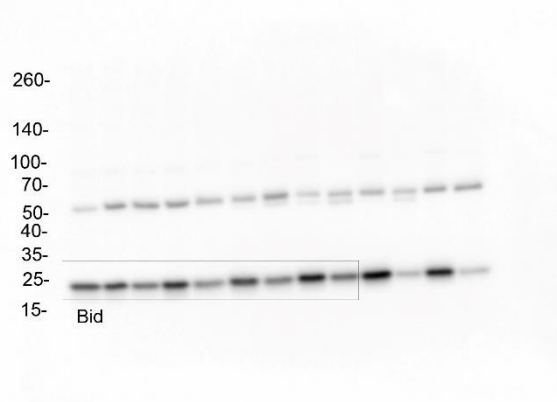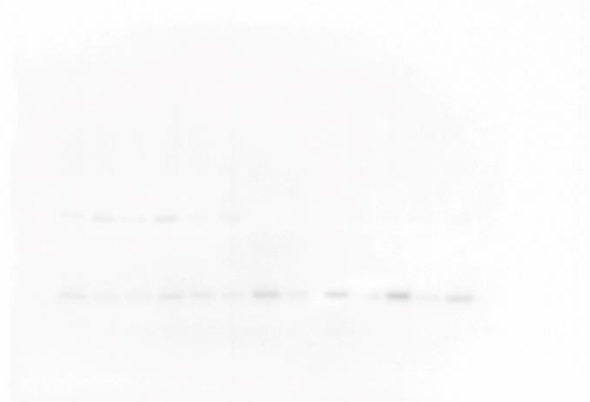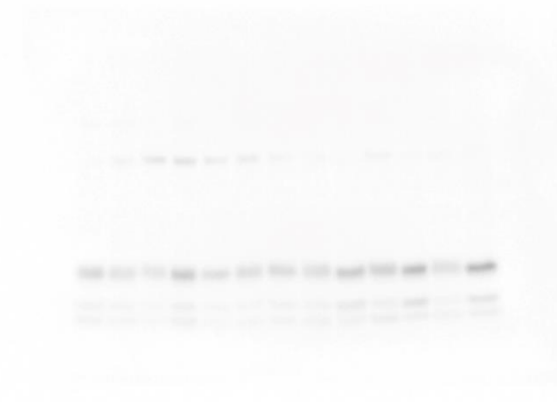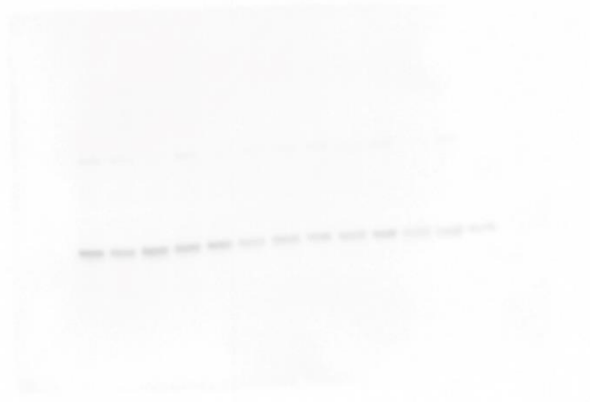

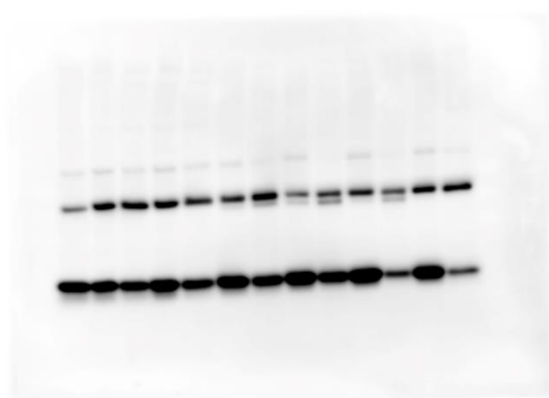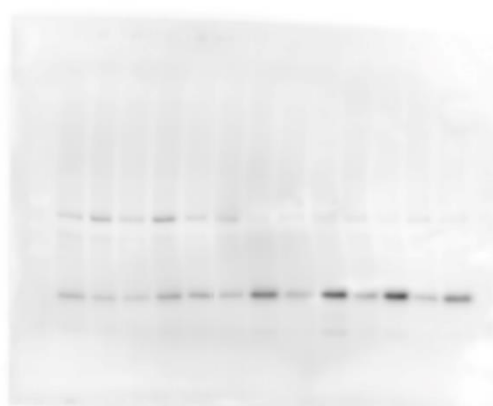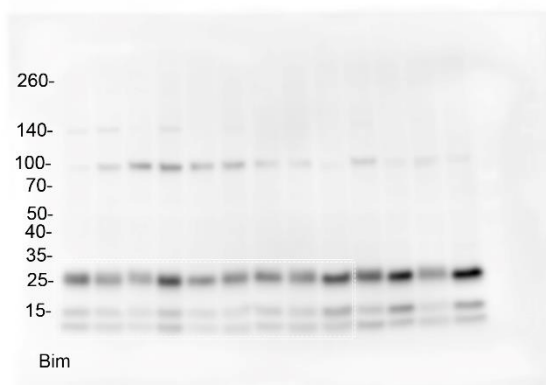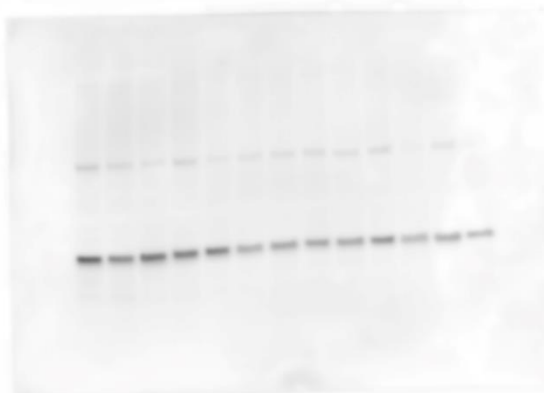

---

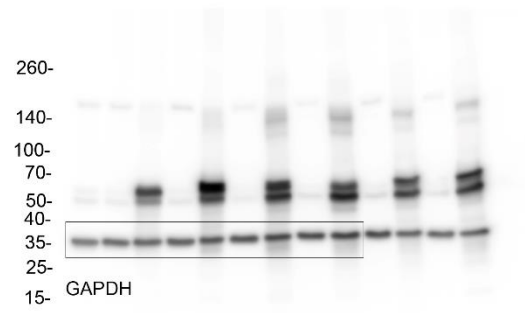

---

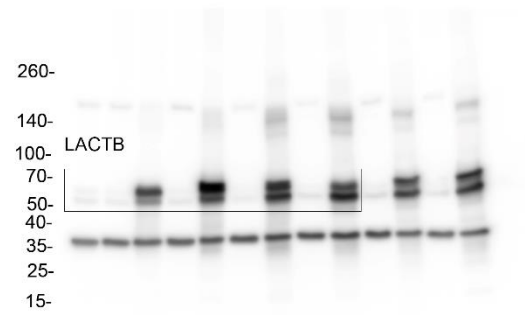

---

Supplement: Supplementary file 1 — Supplementary Material 1 [file 10495_2022_1775_MOESM1_ESM.pdf]

**Figure 2C**

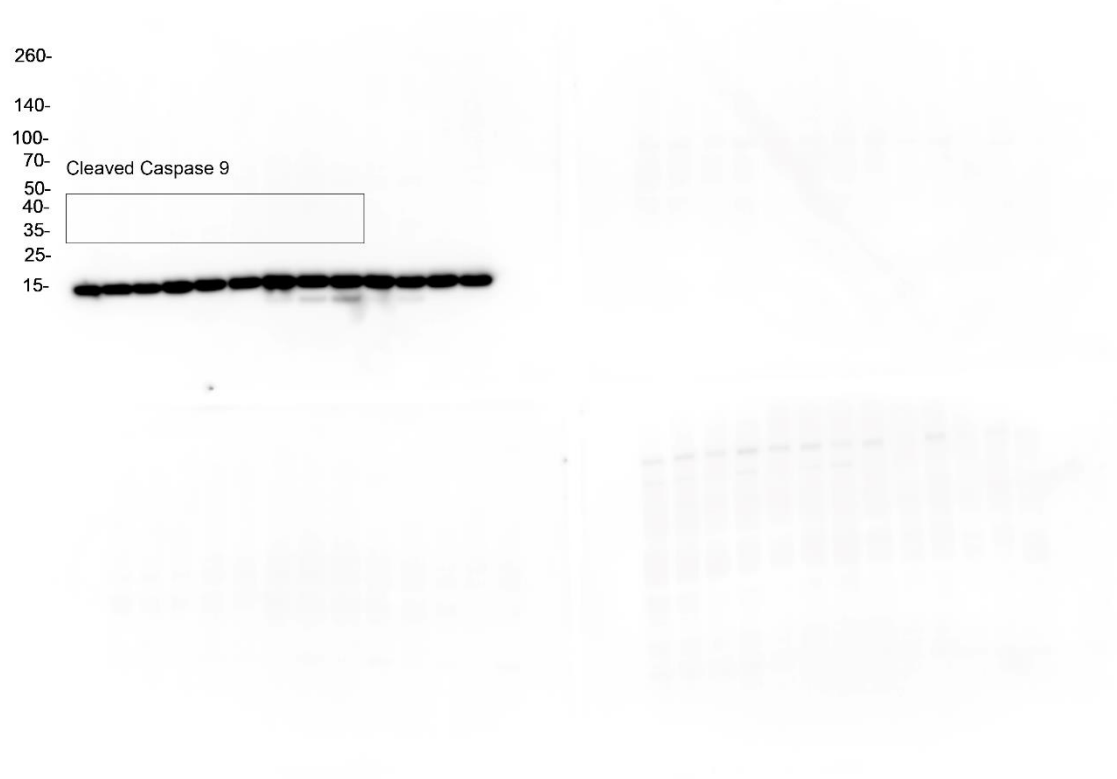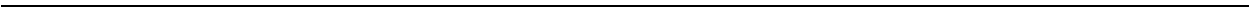

260-

140-

100-

70-

50-

40-

35-

25-

15-

Cleaved PARP

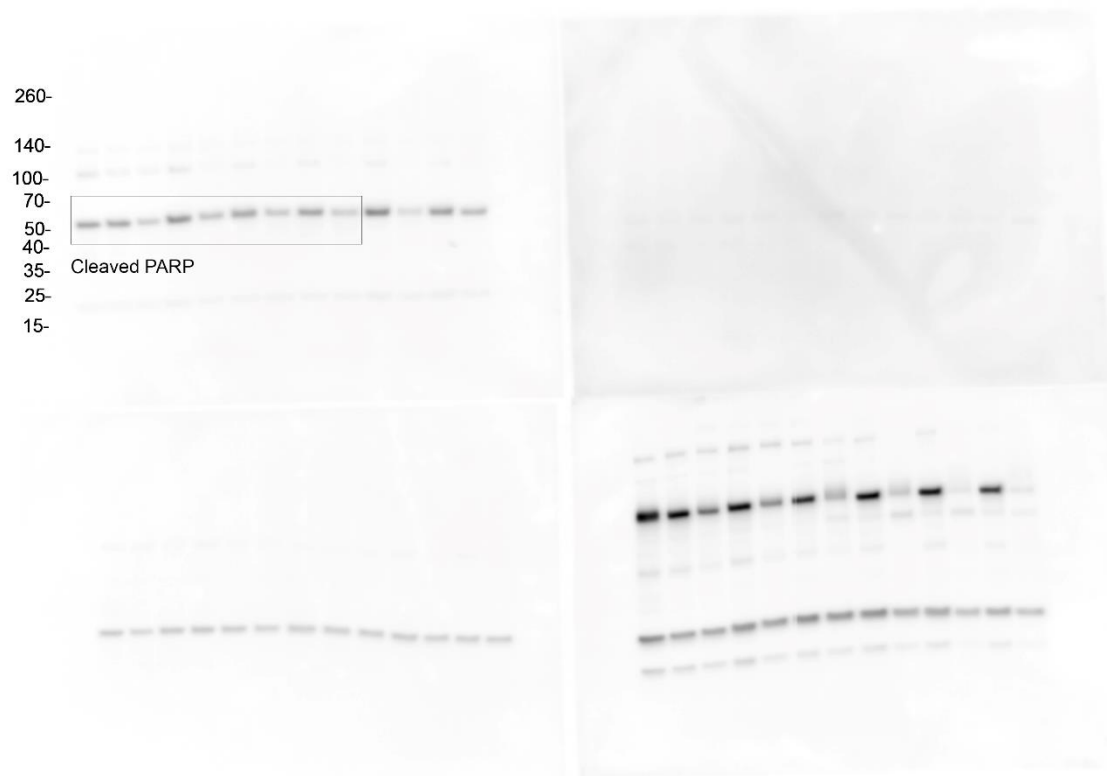

260-  
140-  
100-  
70-  
50-  
40-  
35-  
25-  
15-

GAPDH

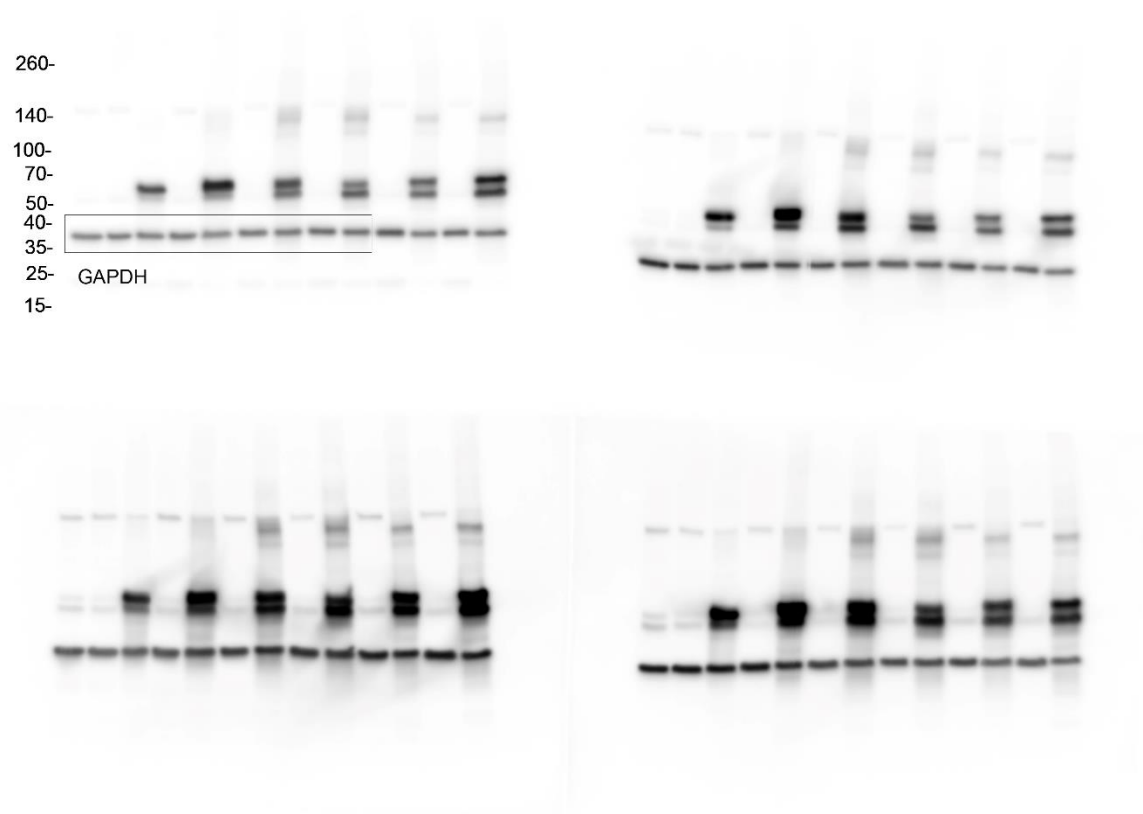

260-

140-

100- LACTB

70-

50-

40-

35-

25-

15-

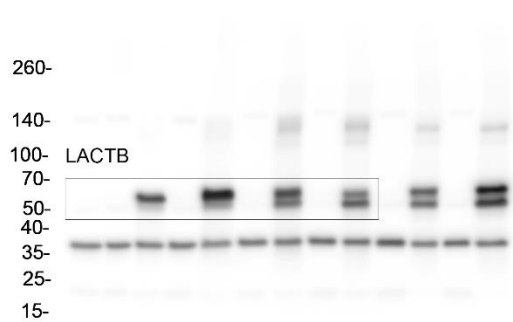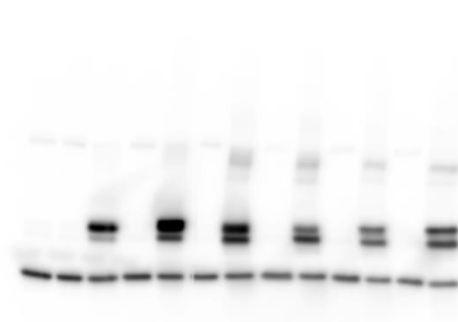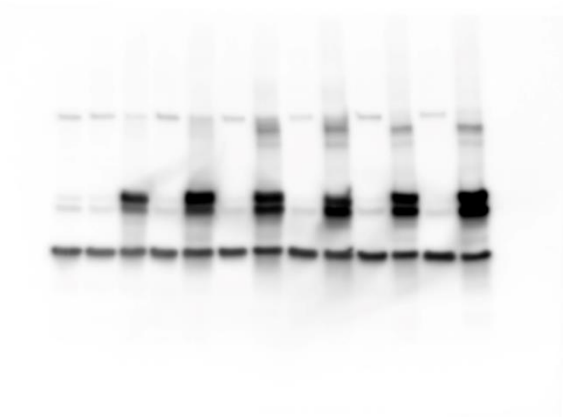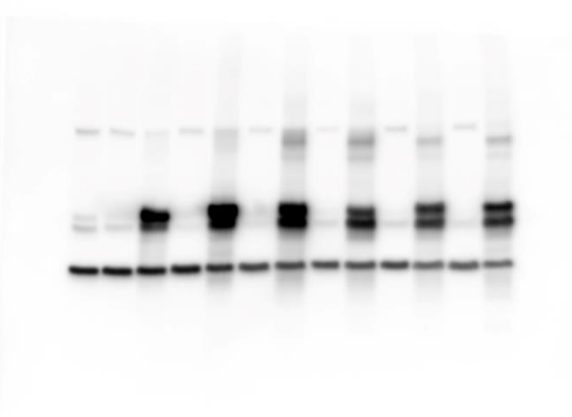

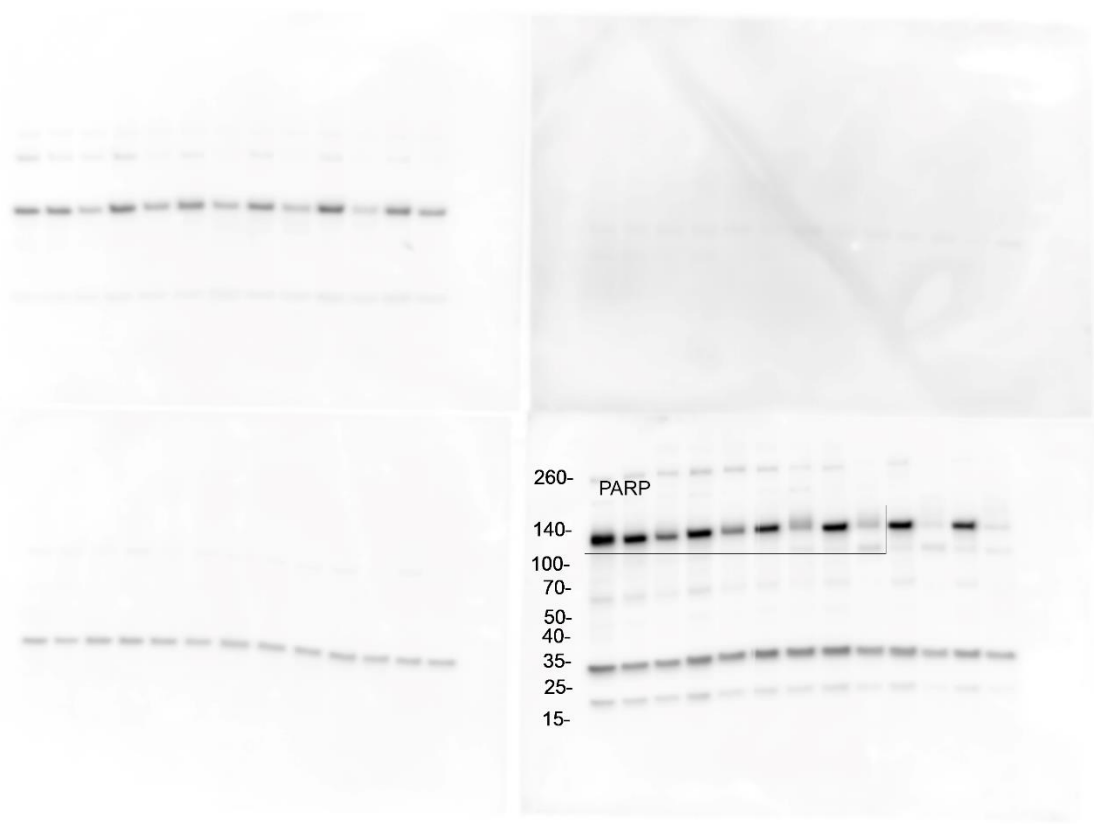

---

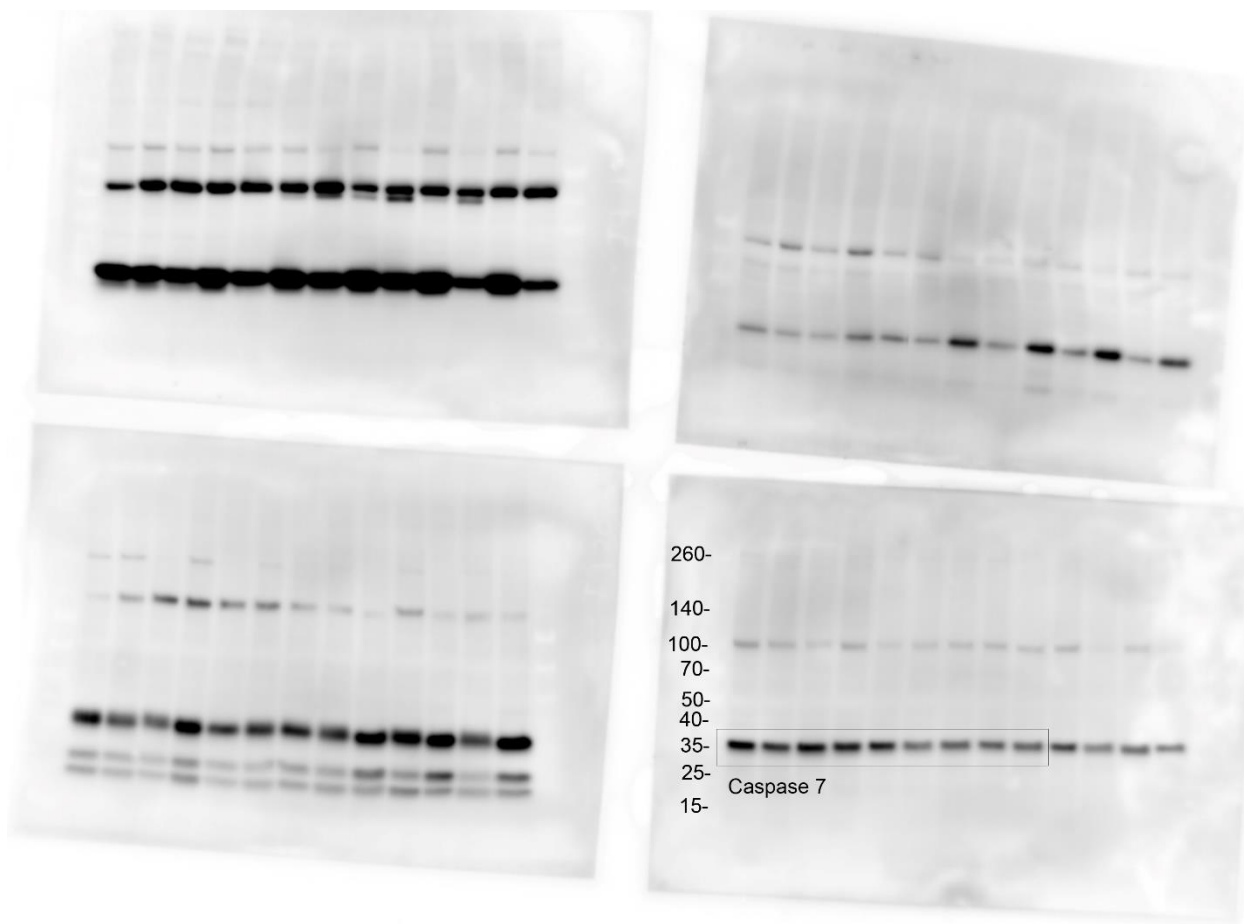

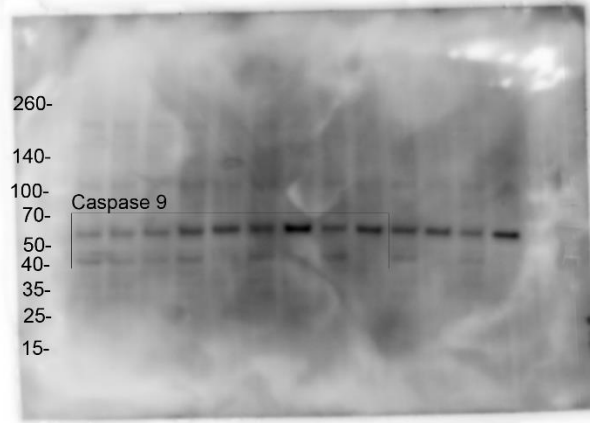

---

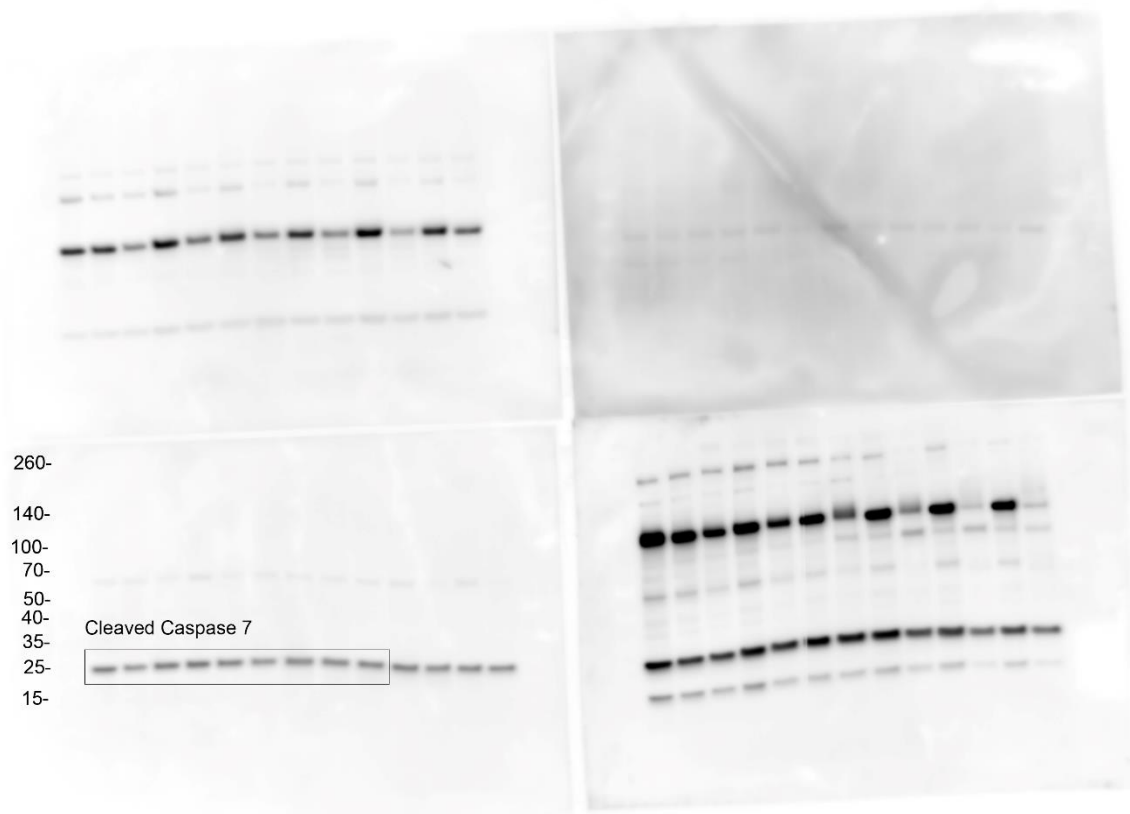

Supplement: Supplementary file 2 — Supplementary Material 2 [file 10495_2022_1775_MOESM2_ESM.pdf]

**Figure 4A**

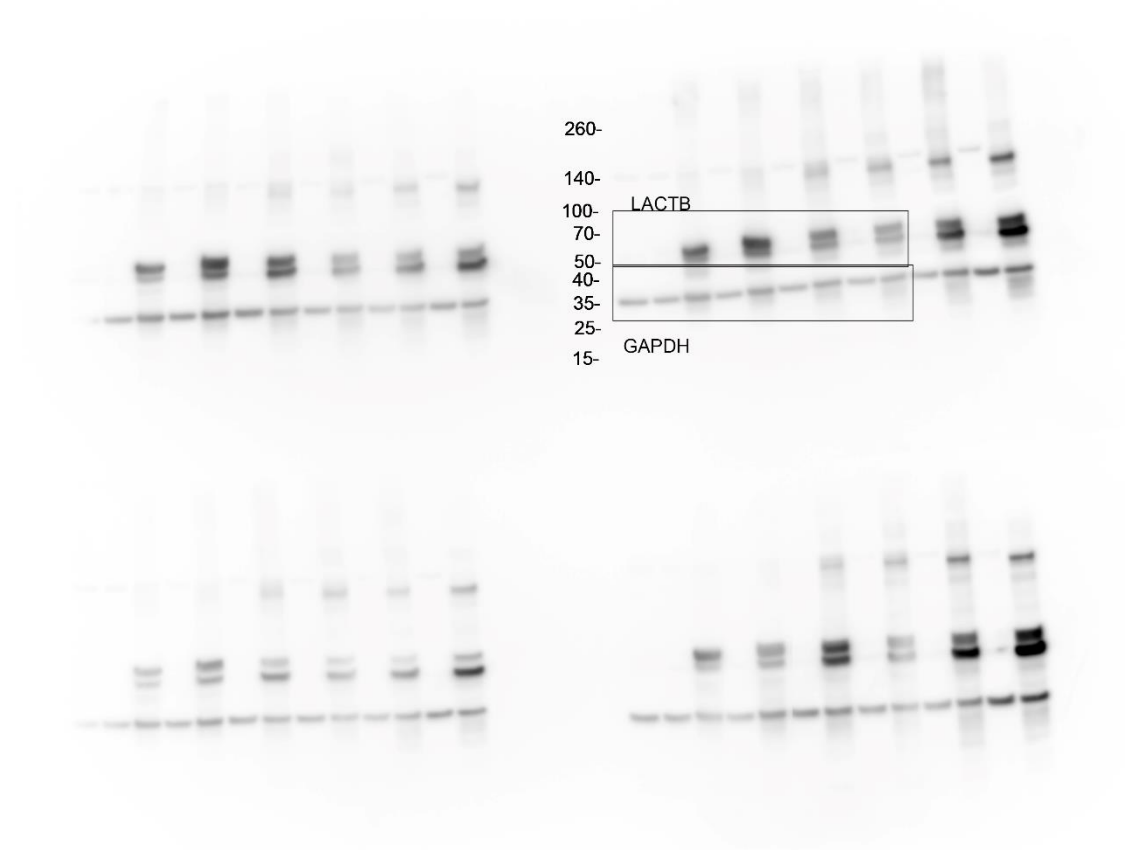

260-  
140-  
100-  
70-  
50-  
40-  
35-  
25-  
15-

pAKT473

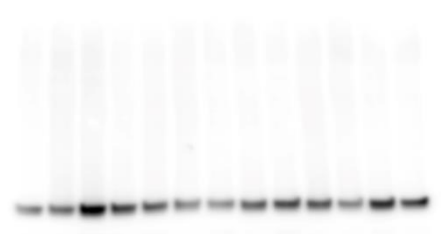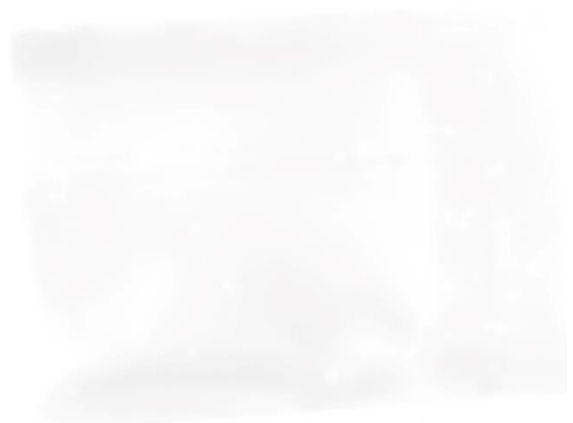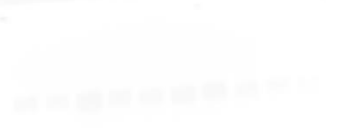

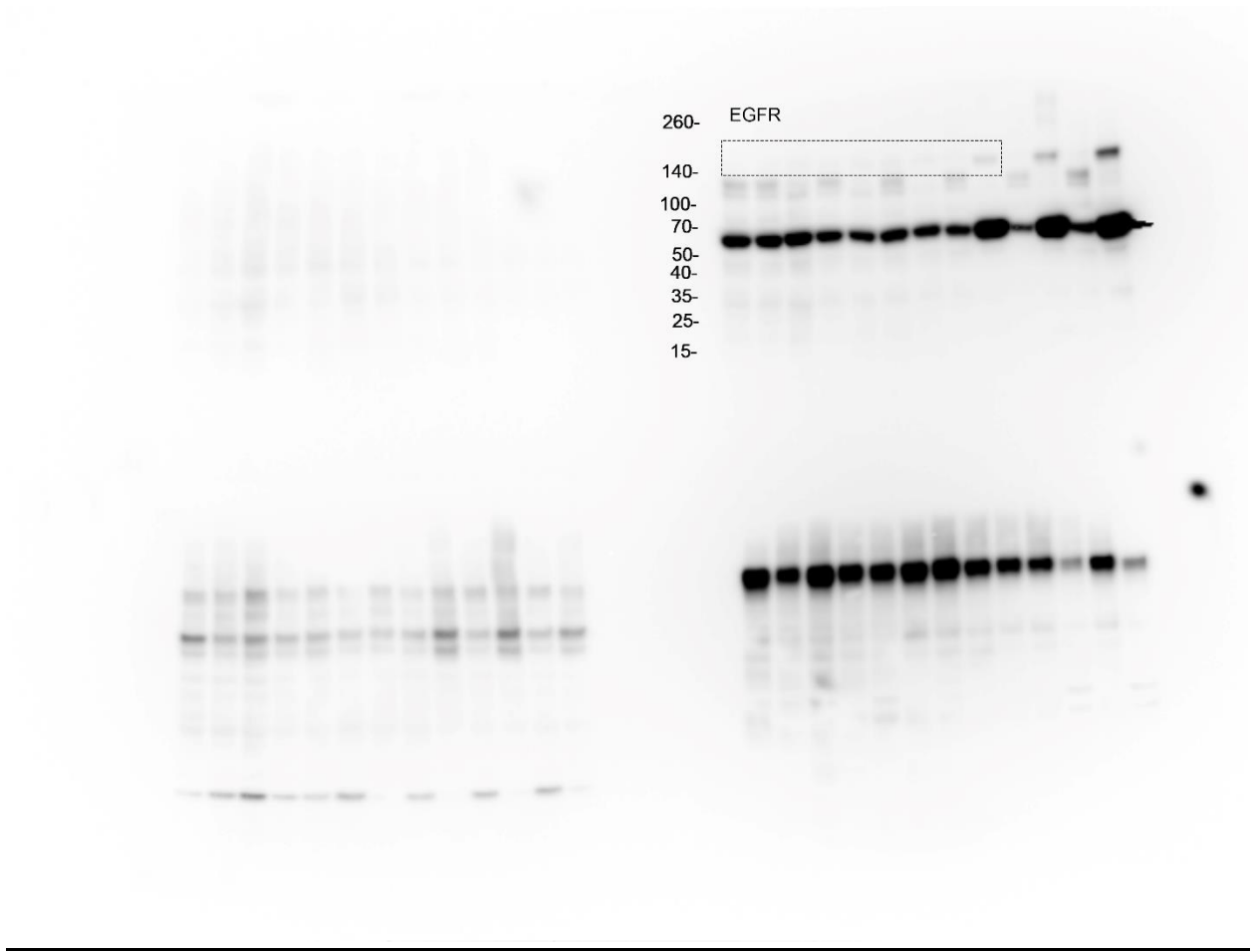

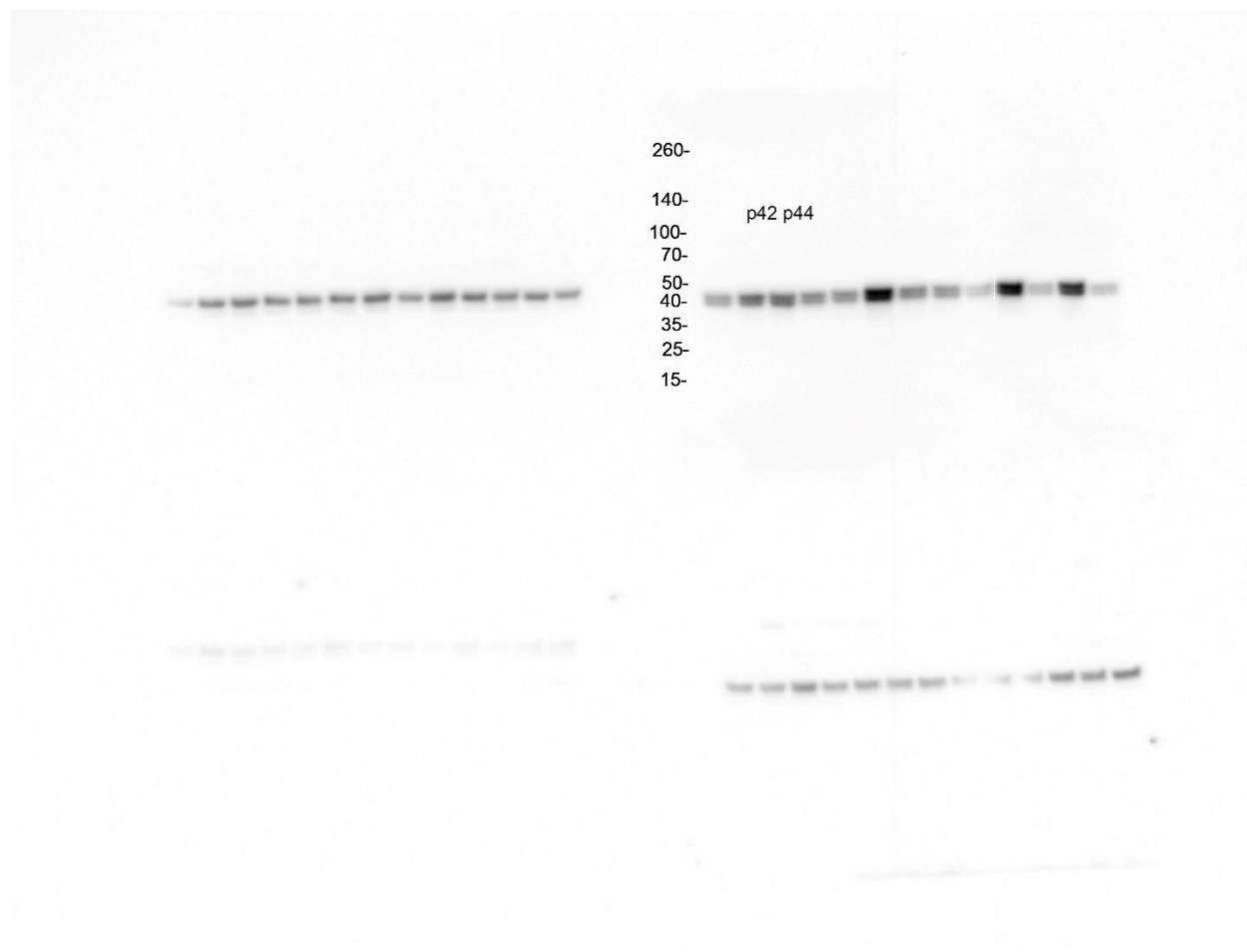

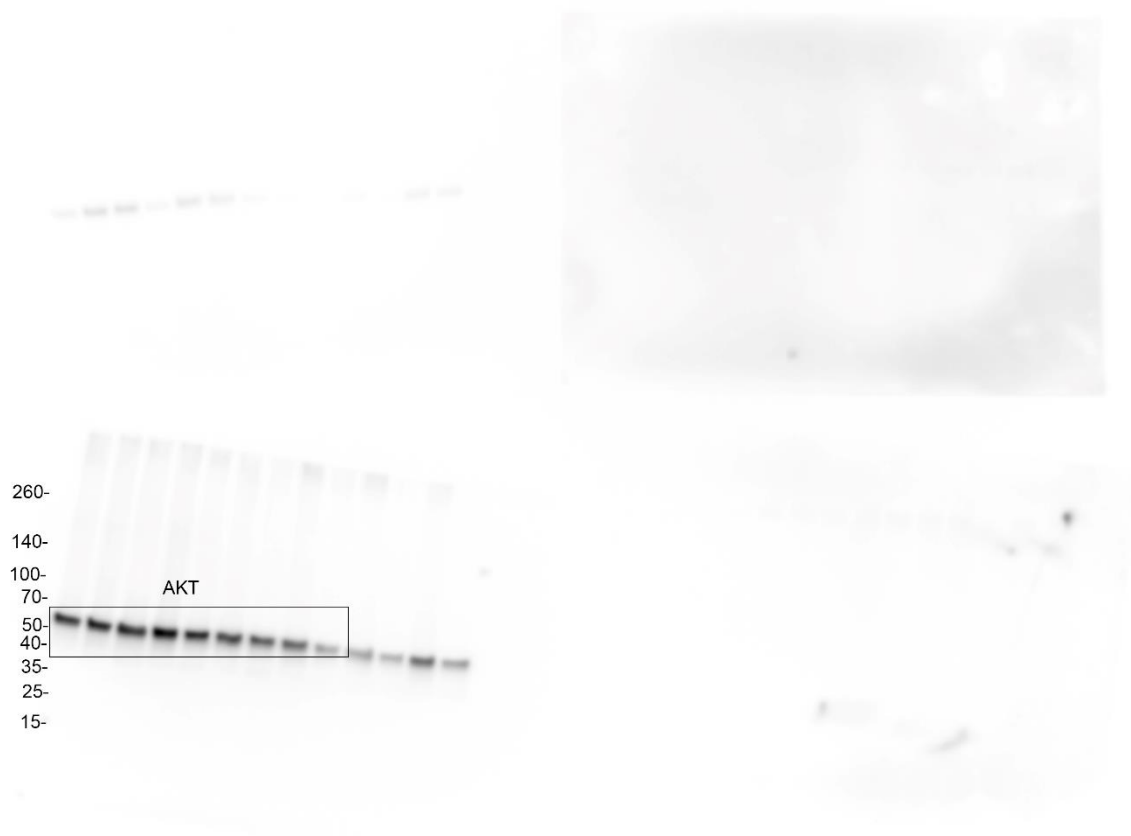

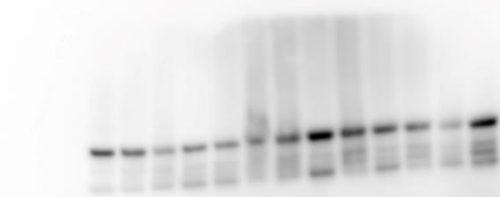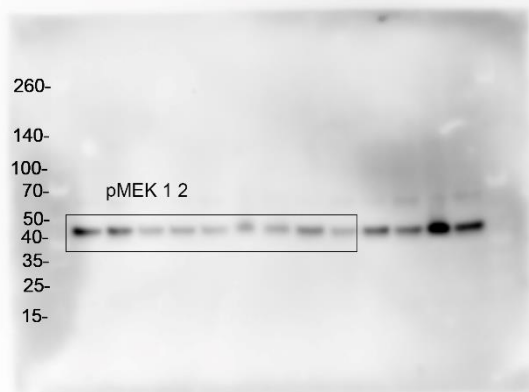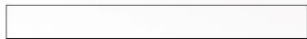

Supplement: Supplementary file 3 — Supplementary Material 3 [file 10495_2022_1775_MOESM3_ESM.pdf]

**Figure 4B**

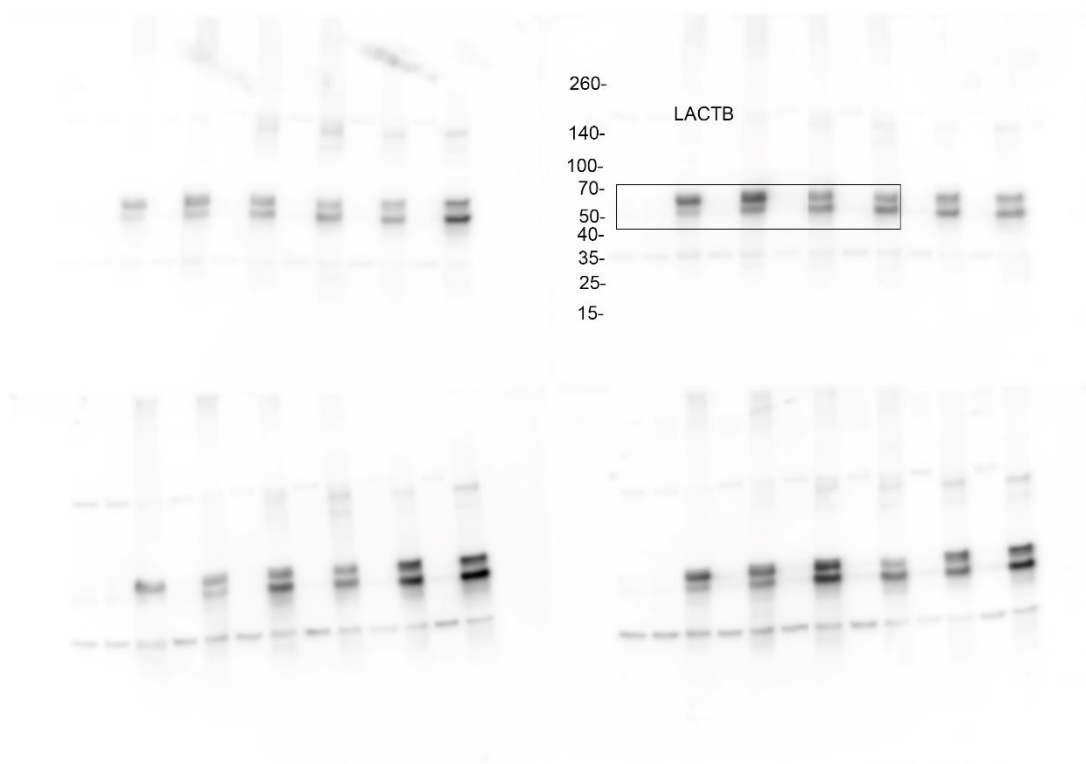

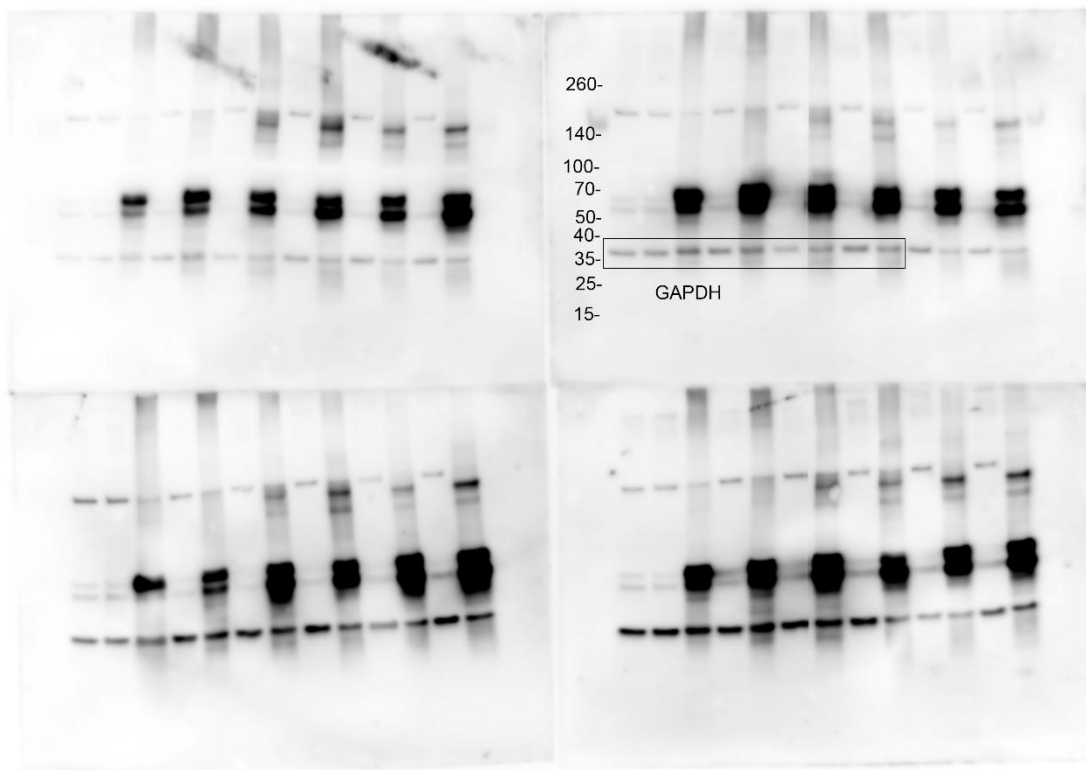

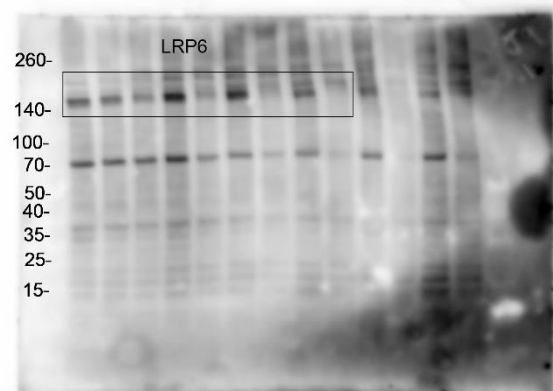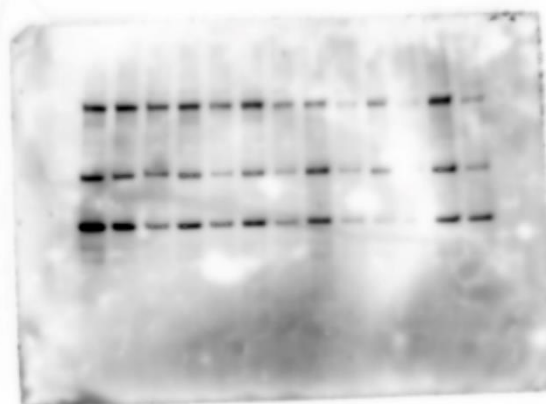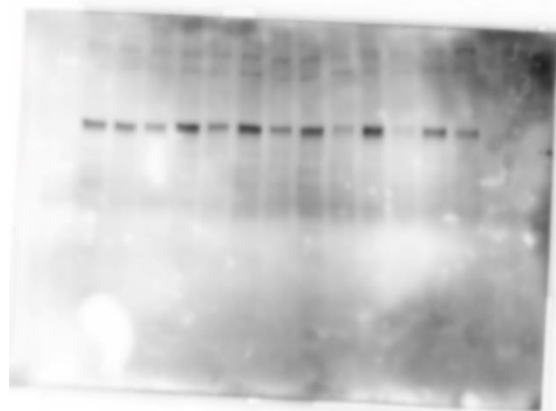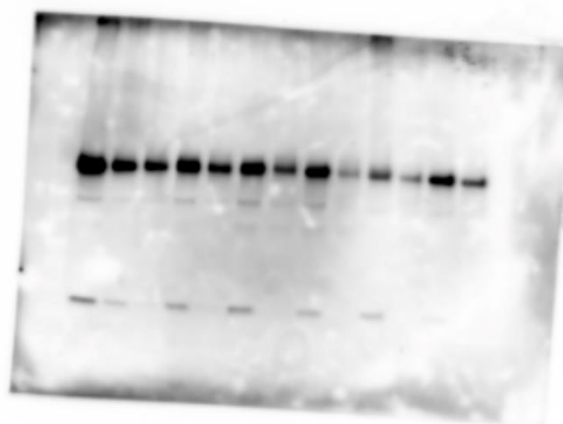

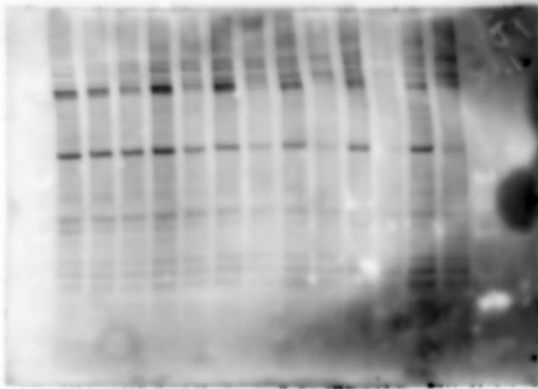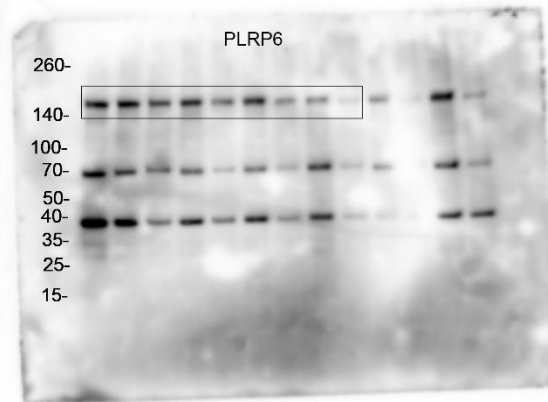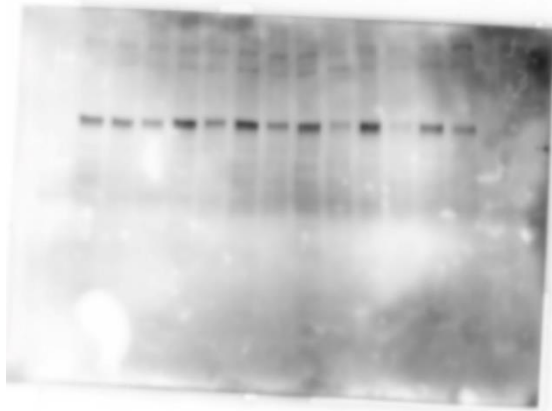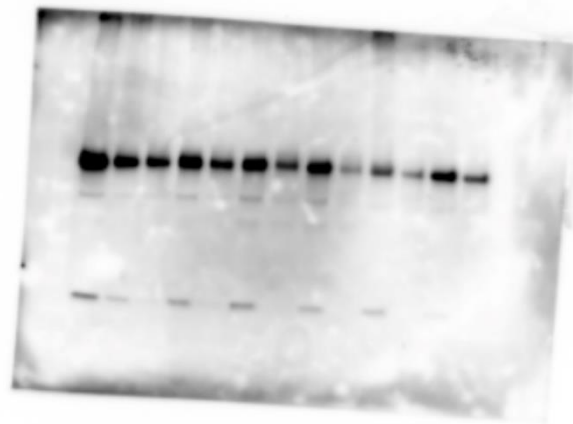

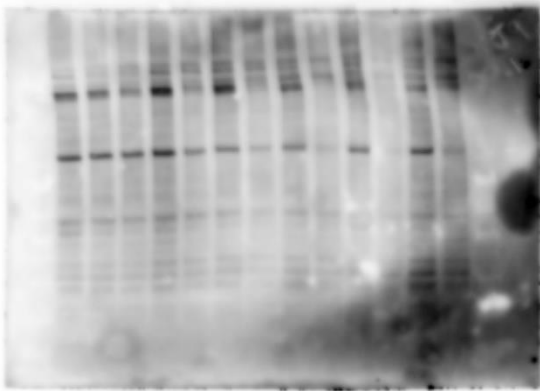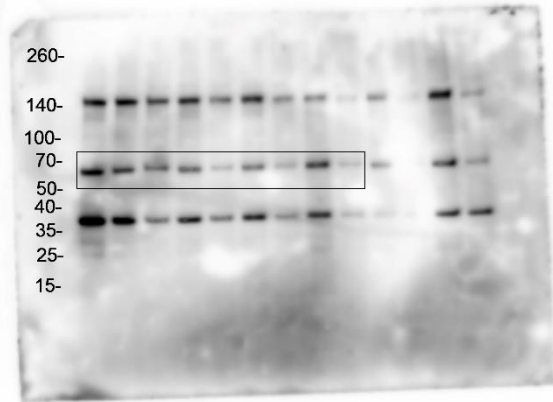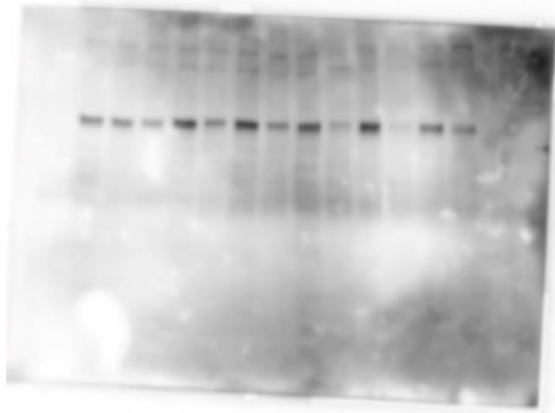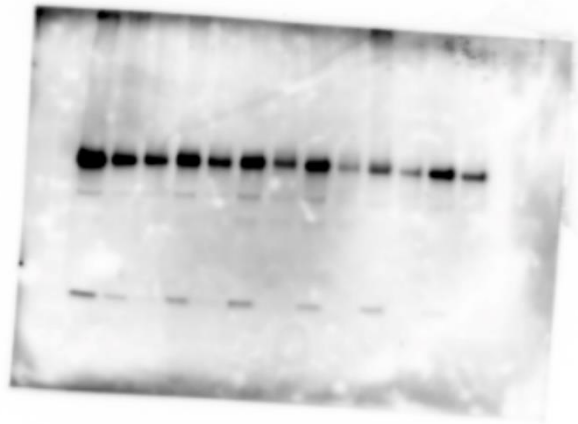

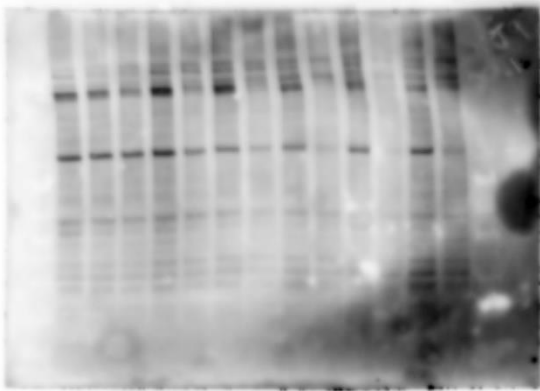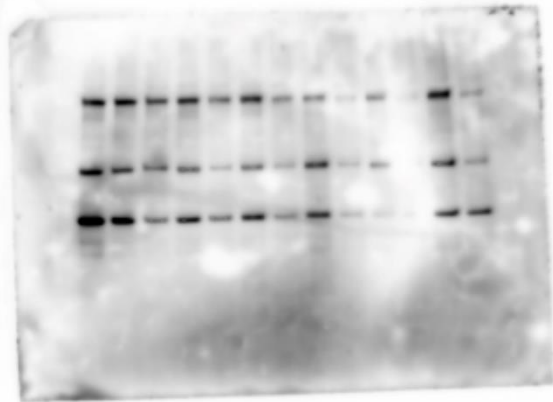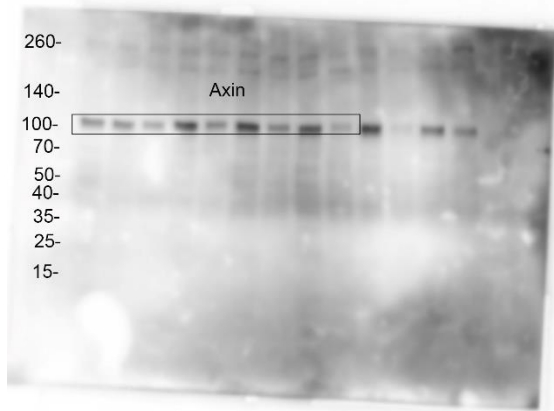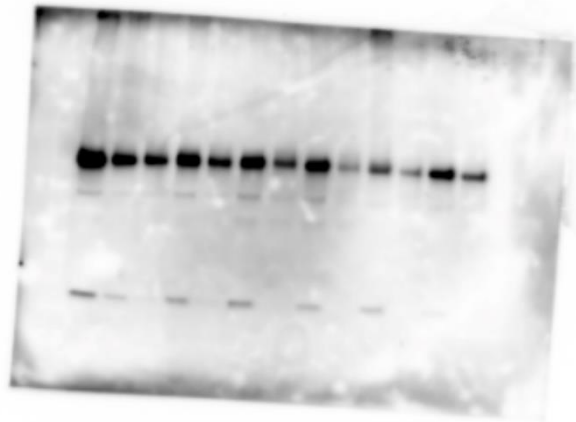

Supplement: Supplementary file 4 — Supplementary Material 4 [file 10495_2022_1775_MOESM4_ESM.pdf]

**Figure 4C**

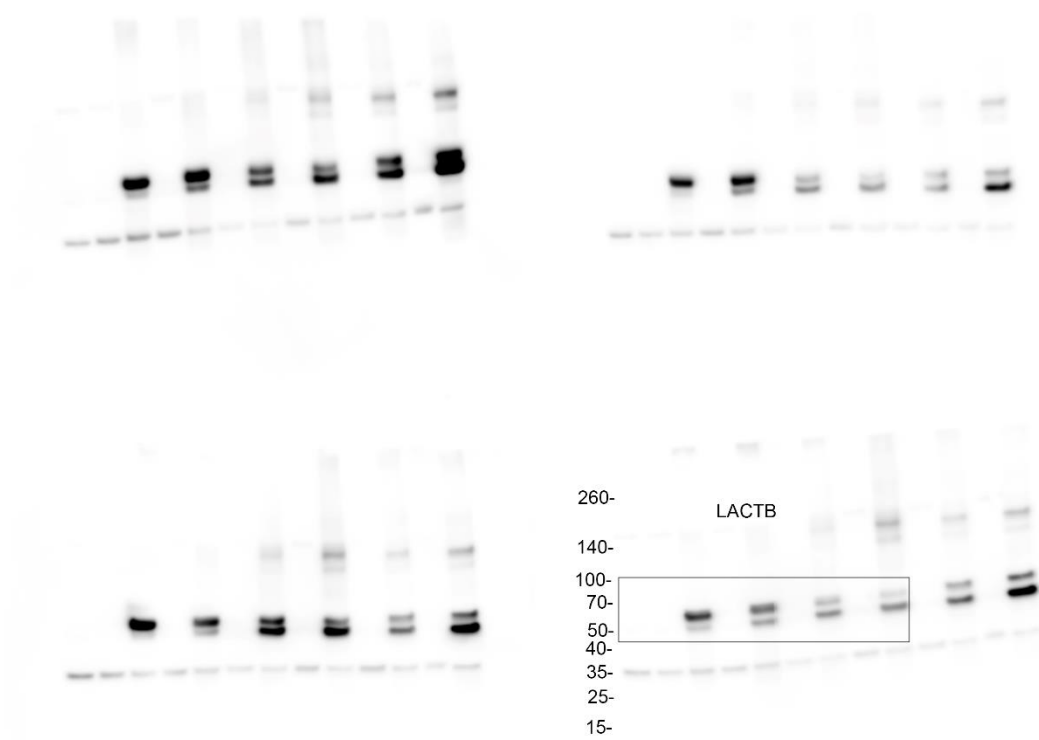

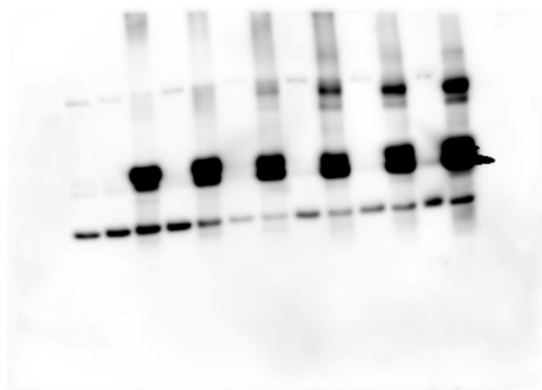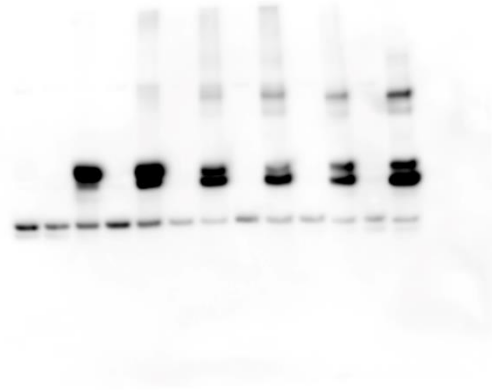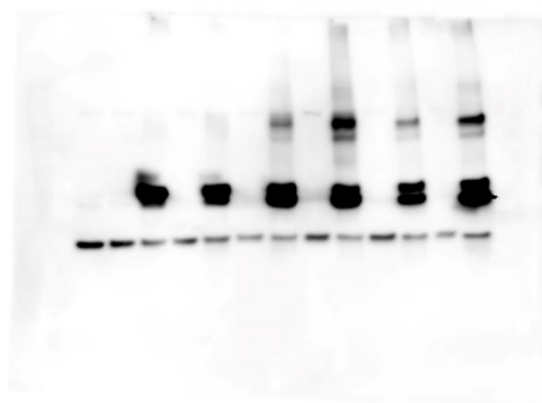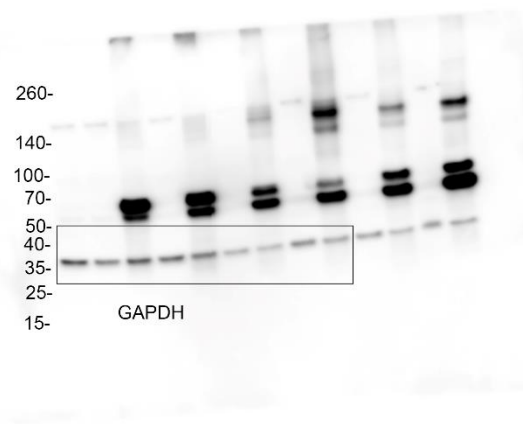

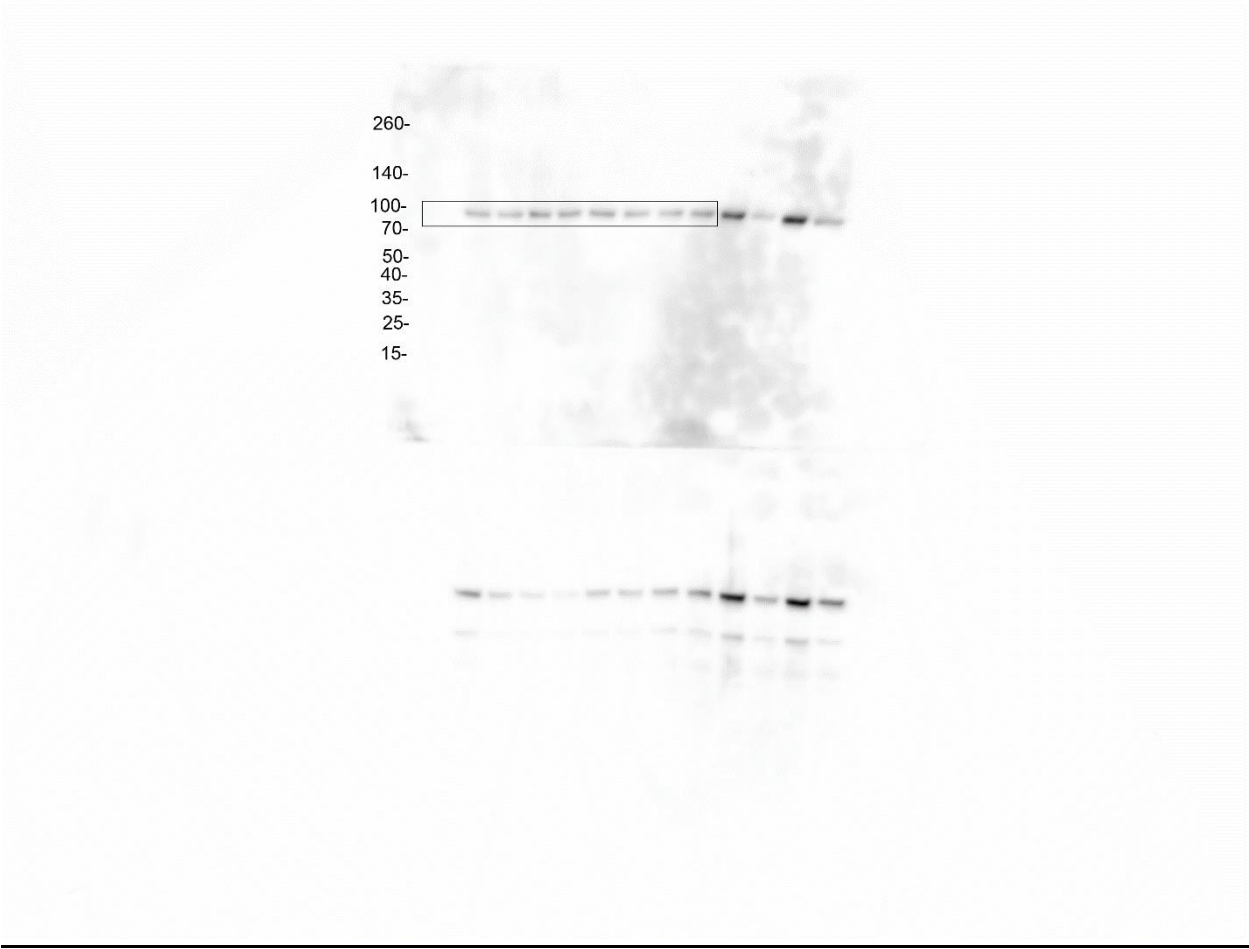

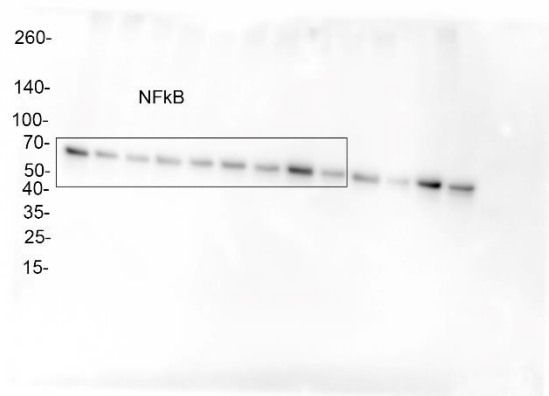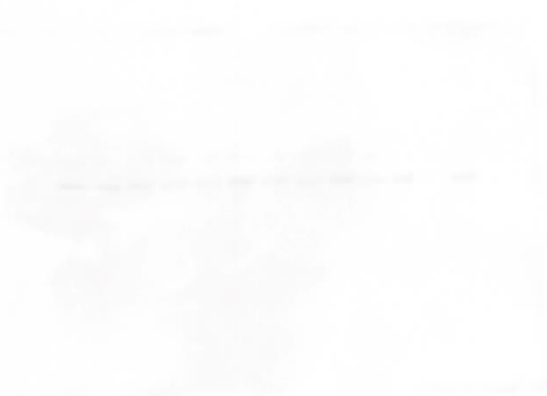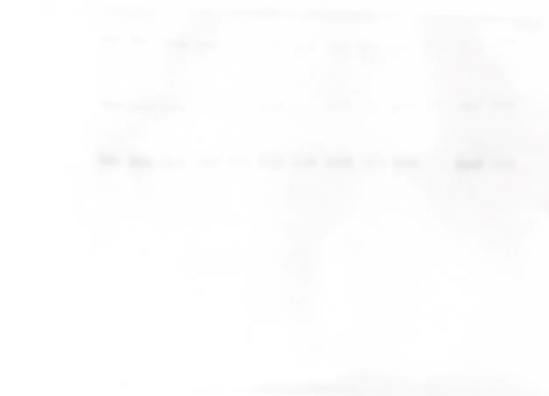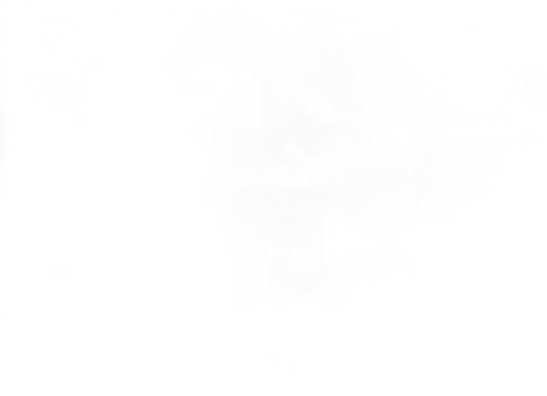

---

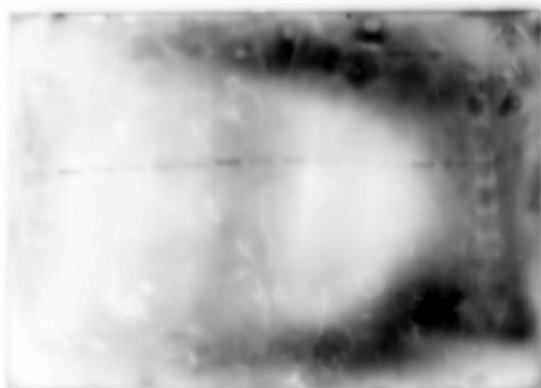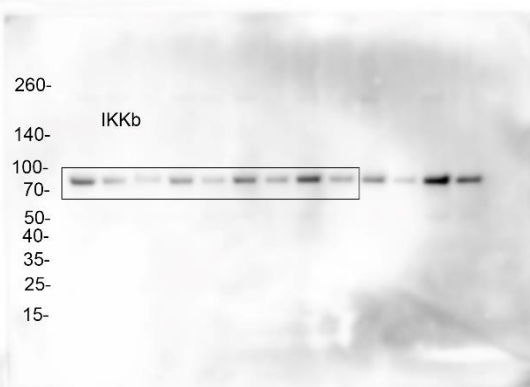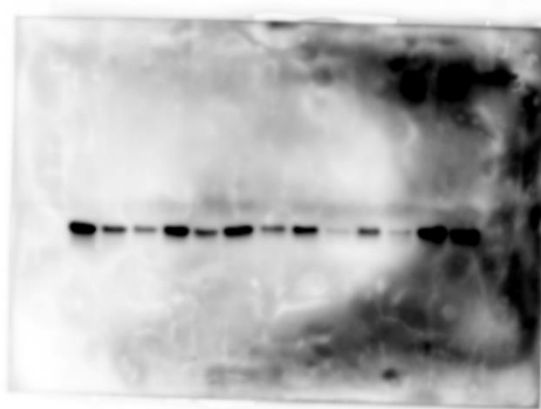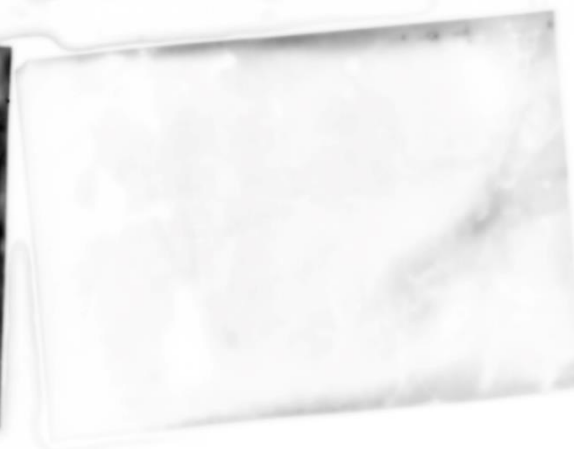

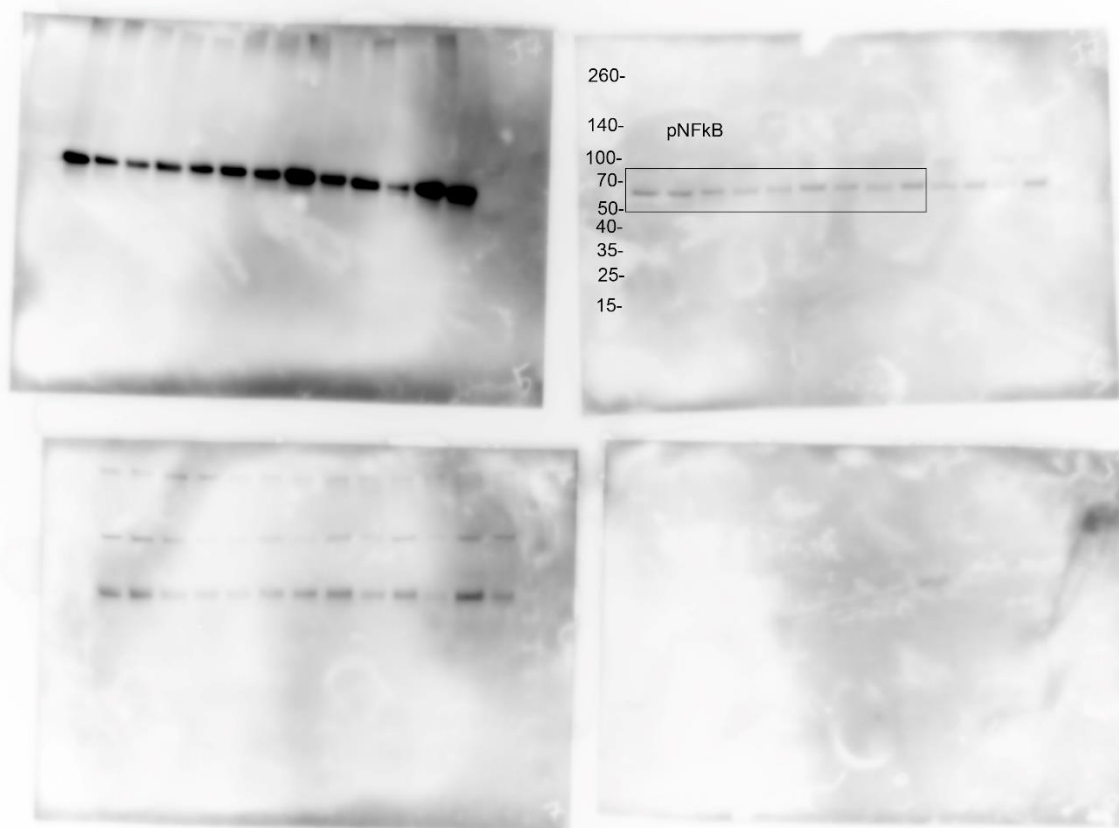

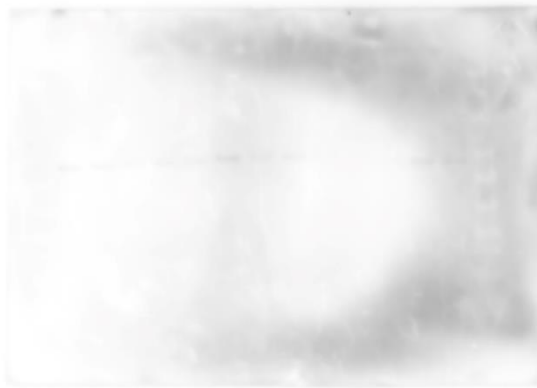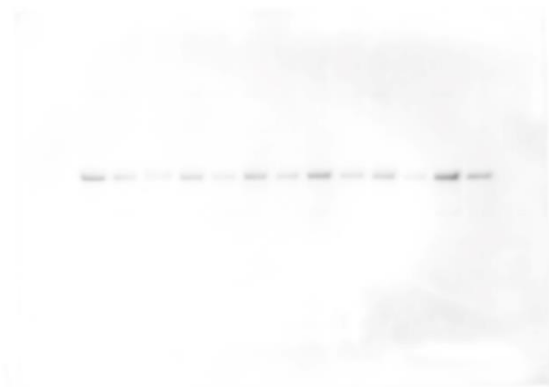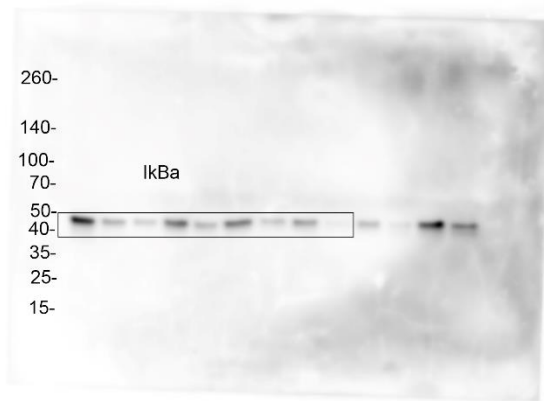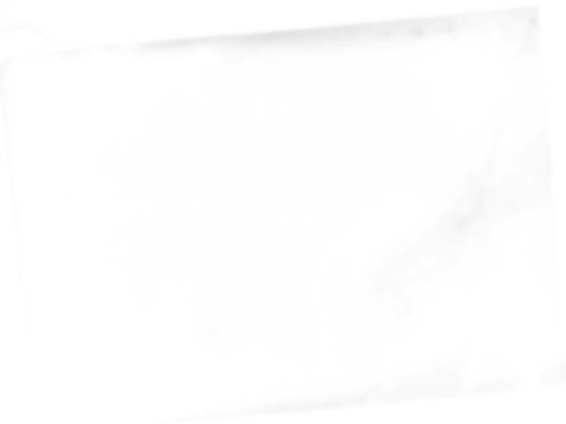

Supplement: Supplementary file 5 — Supplementary Material 5 [file 10495_2022_1775_MOESM5_ESM.pdf]

**Figure 5C**

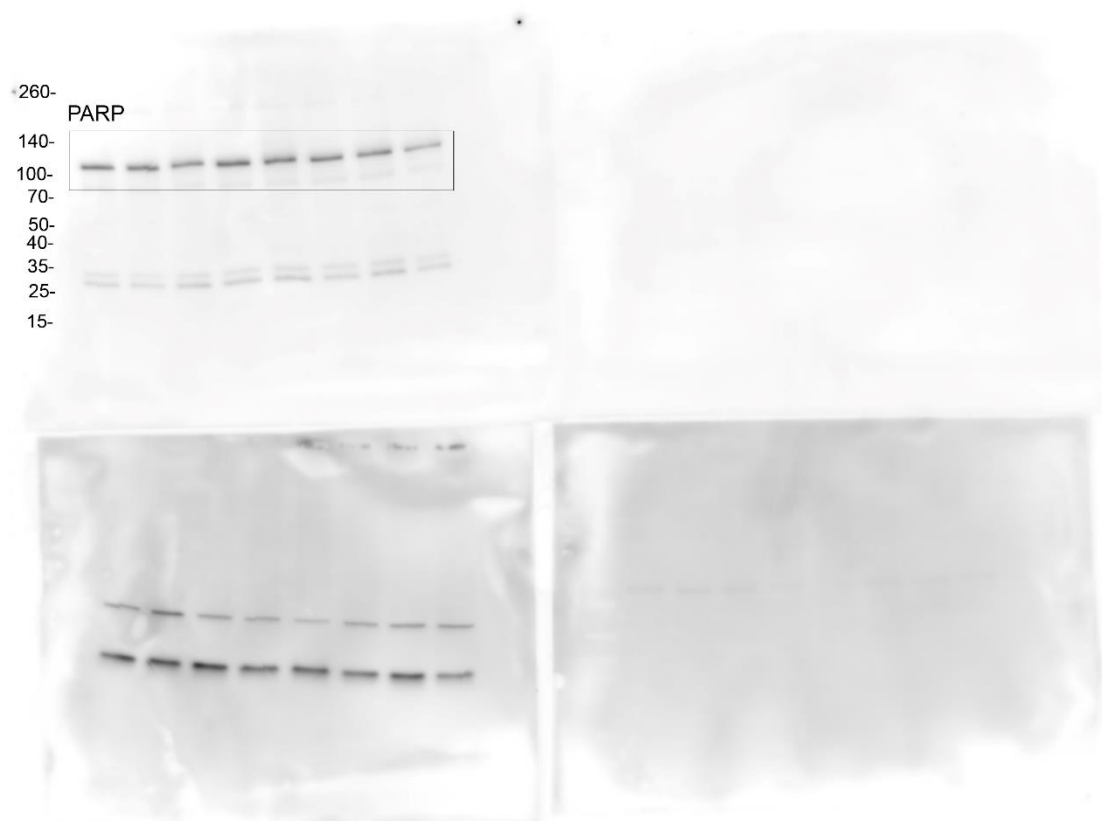

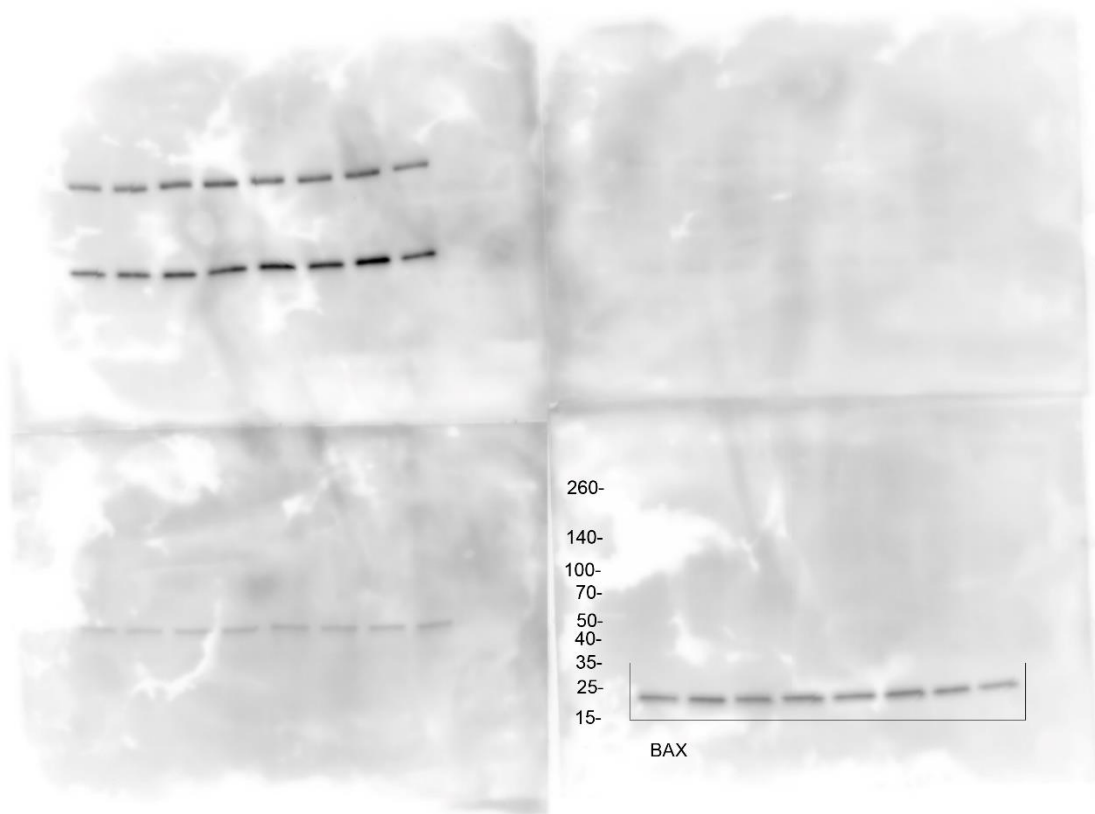

---

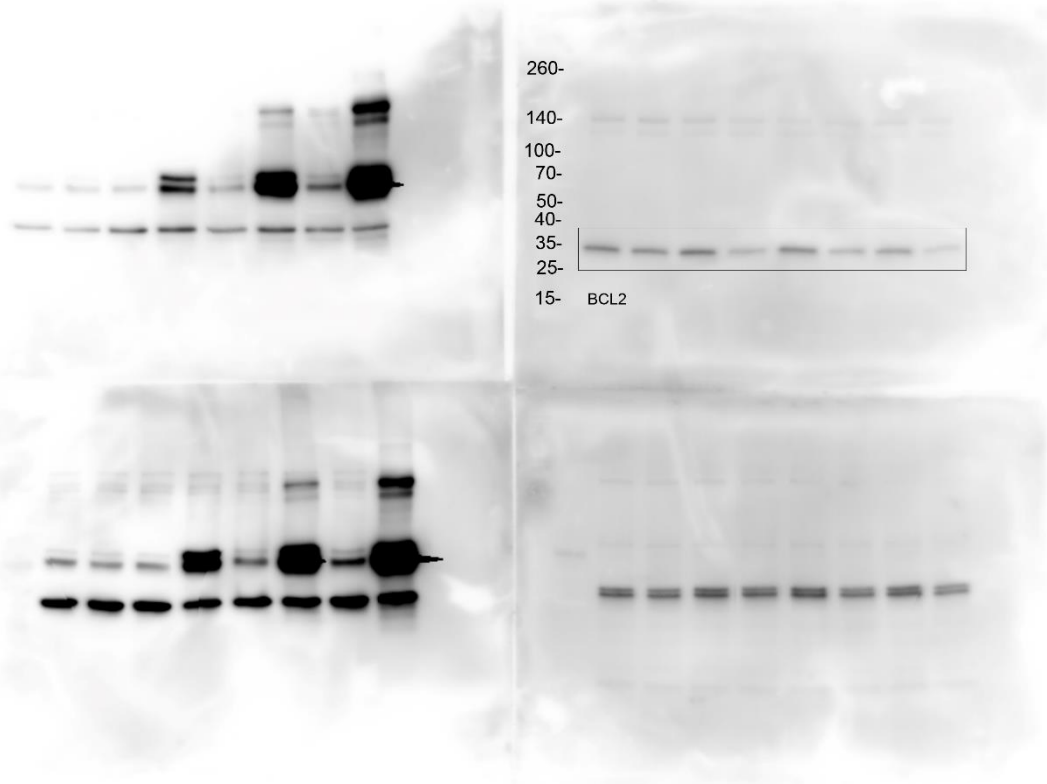

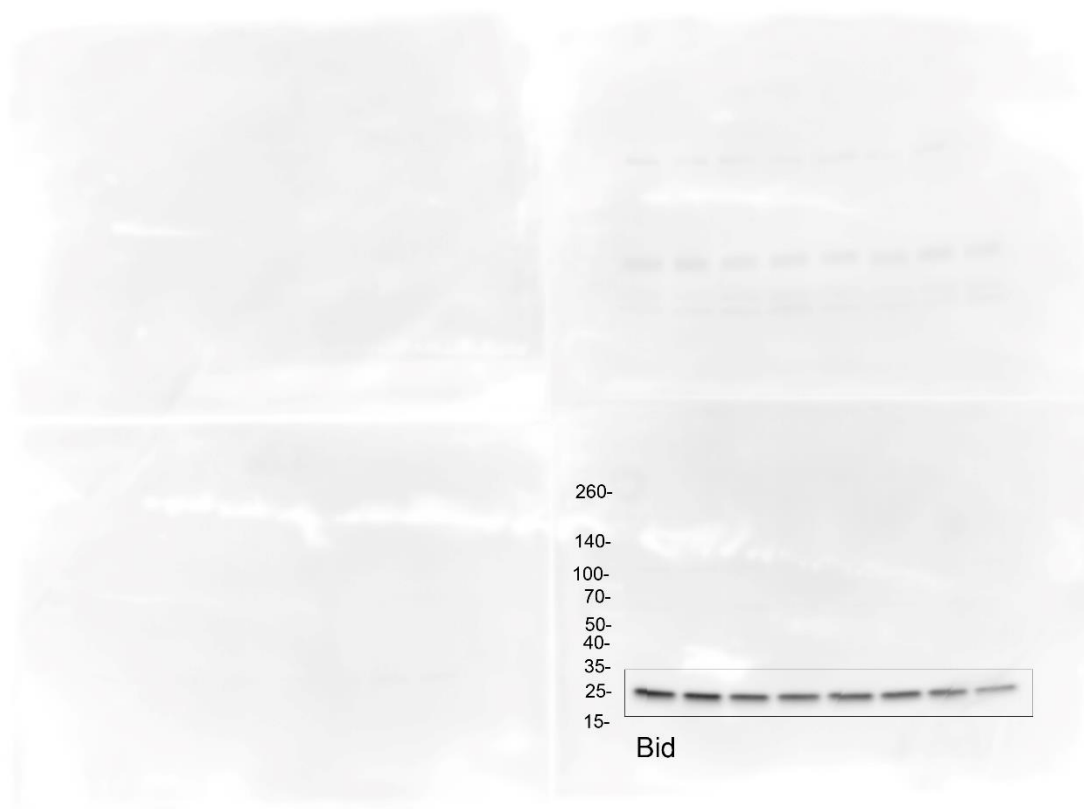

---

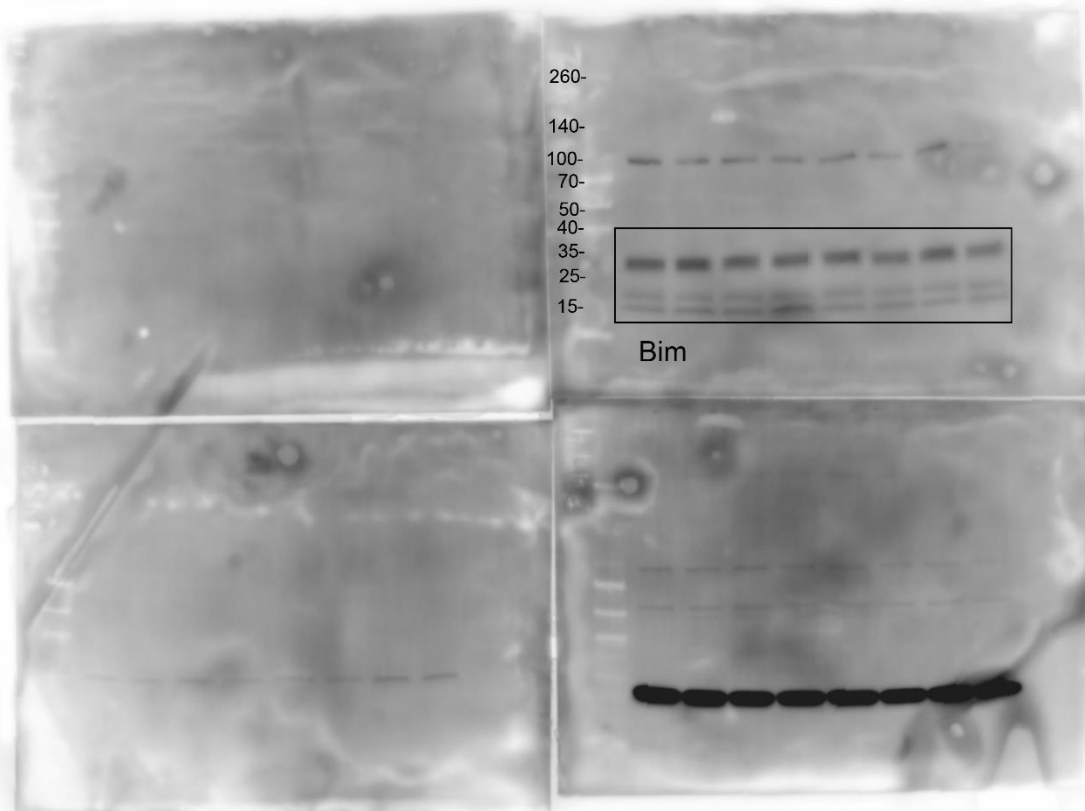

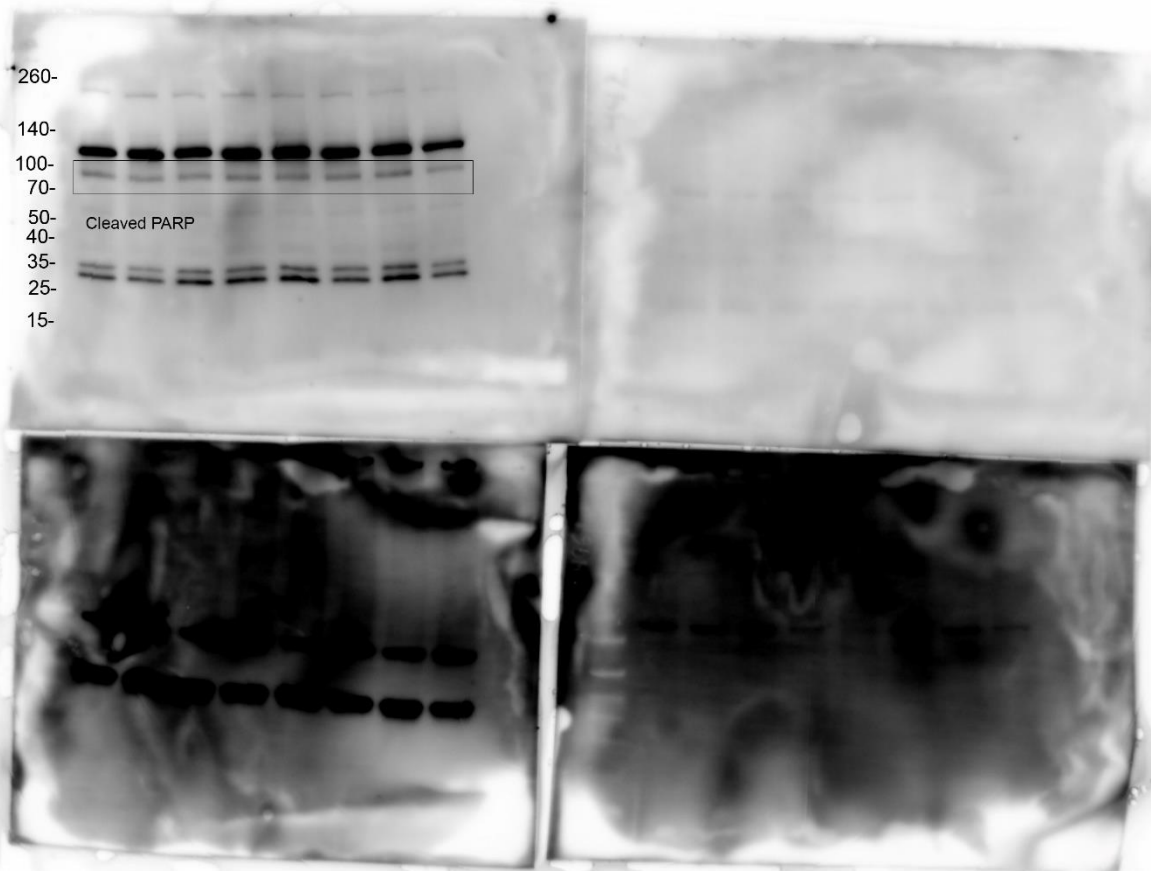

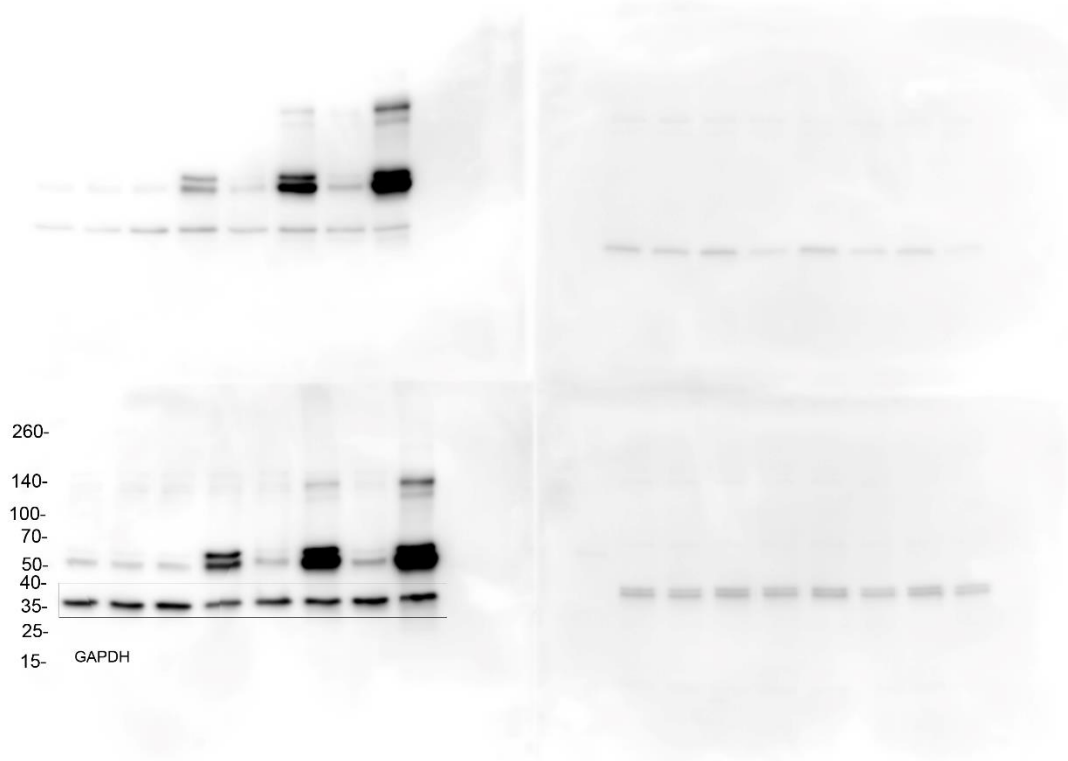

---

Supplement: Supplementary file 6 — Supplementary Material 6 [file 10495_2022_1775_MOESM6_ESM.pdf]

## Supplementary Figure 2

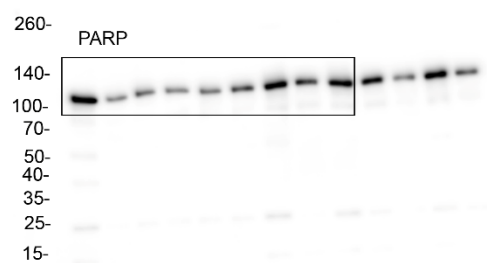

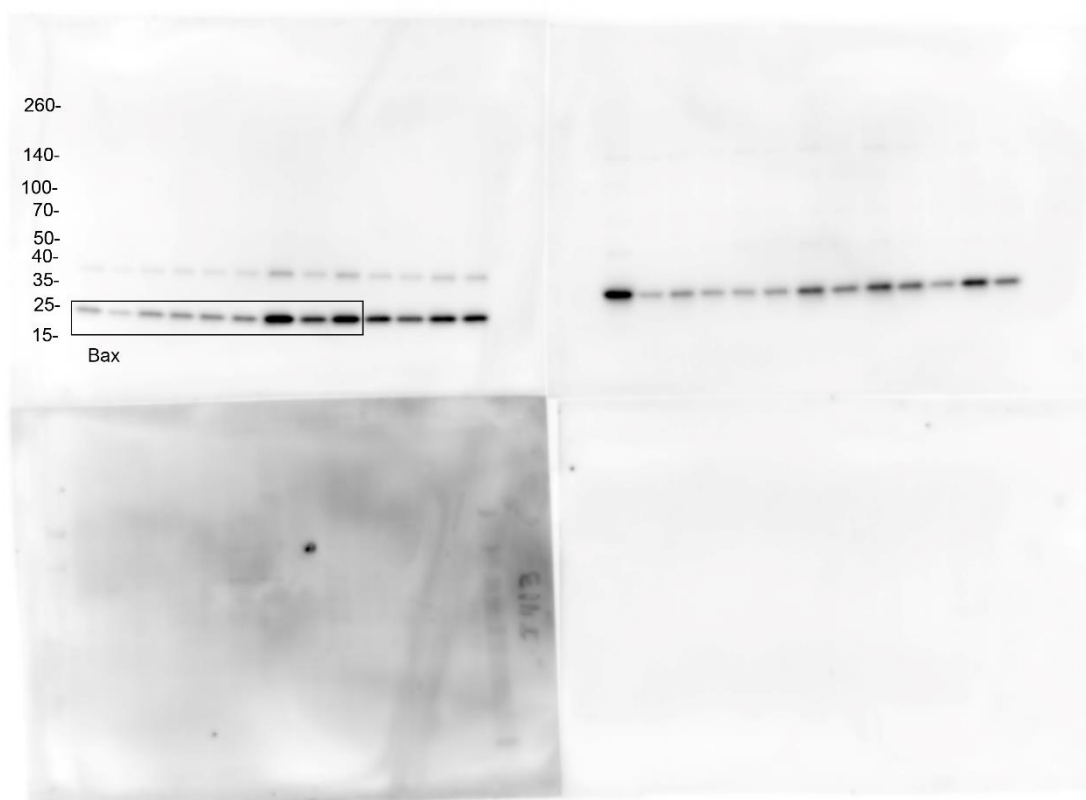

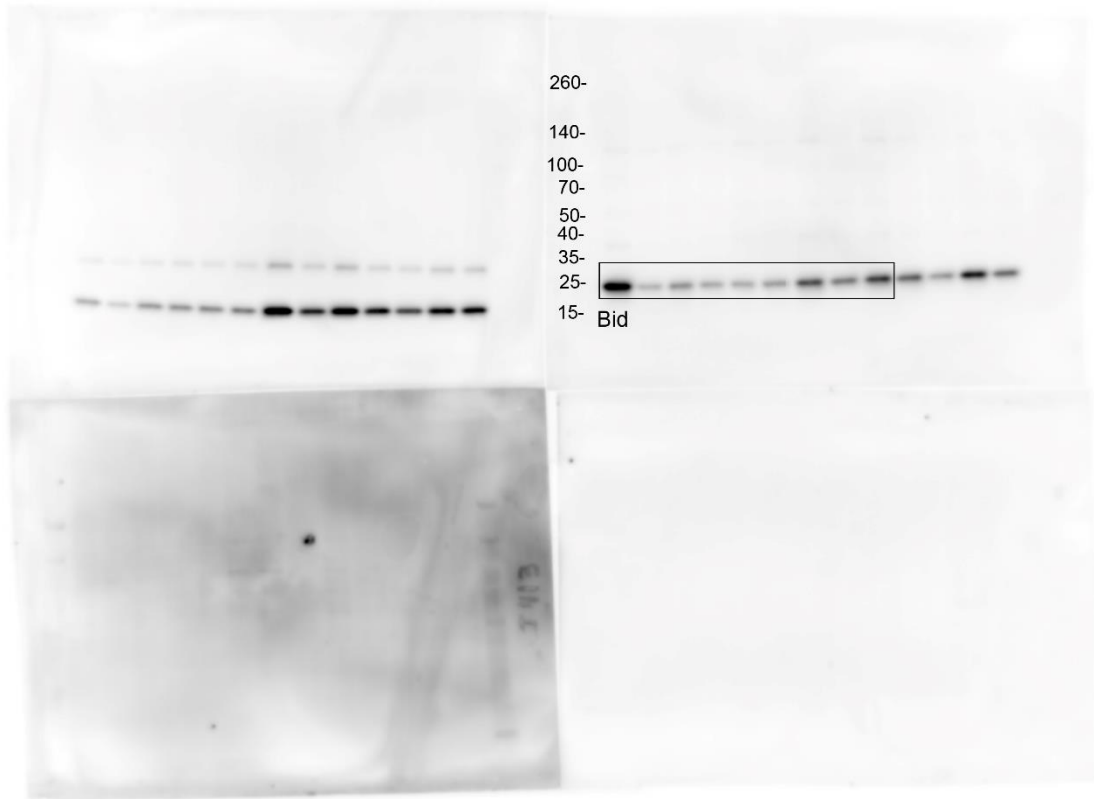

---

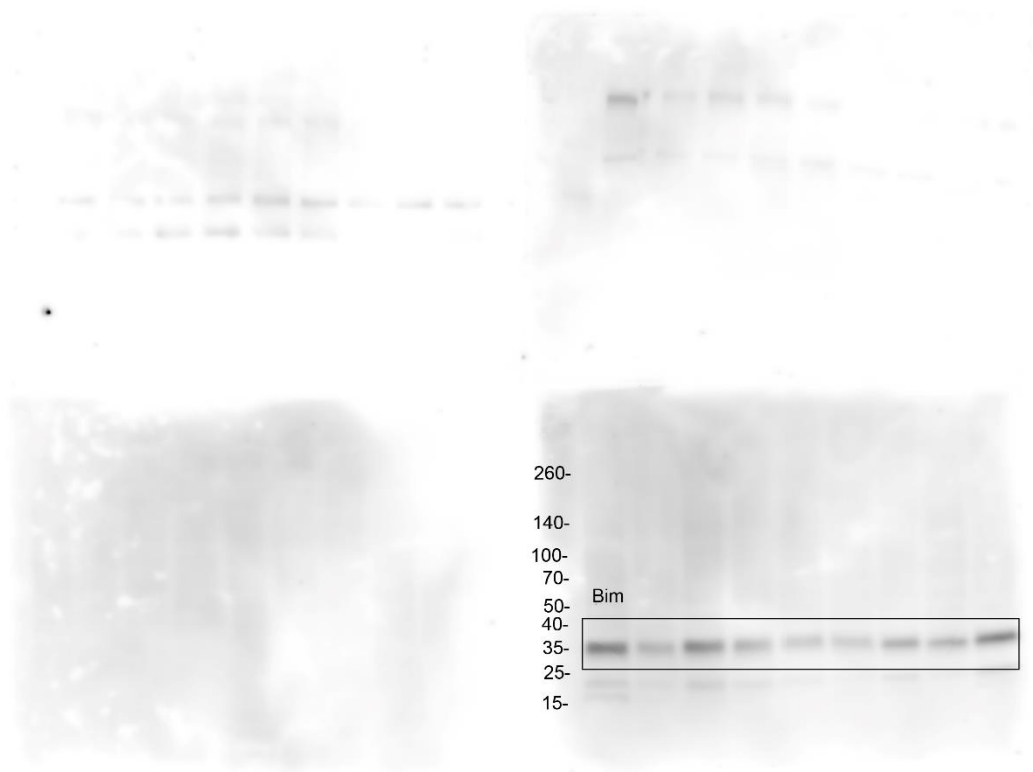

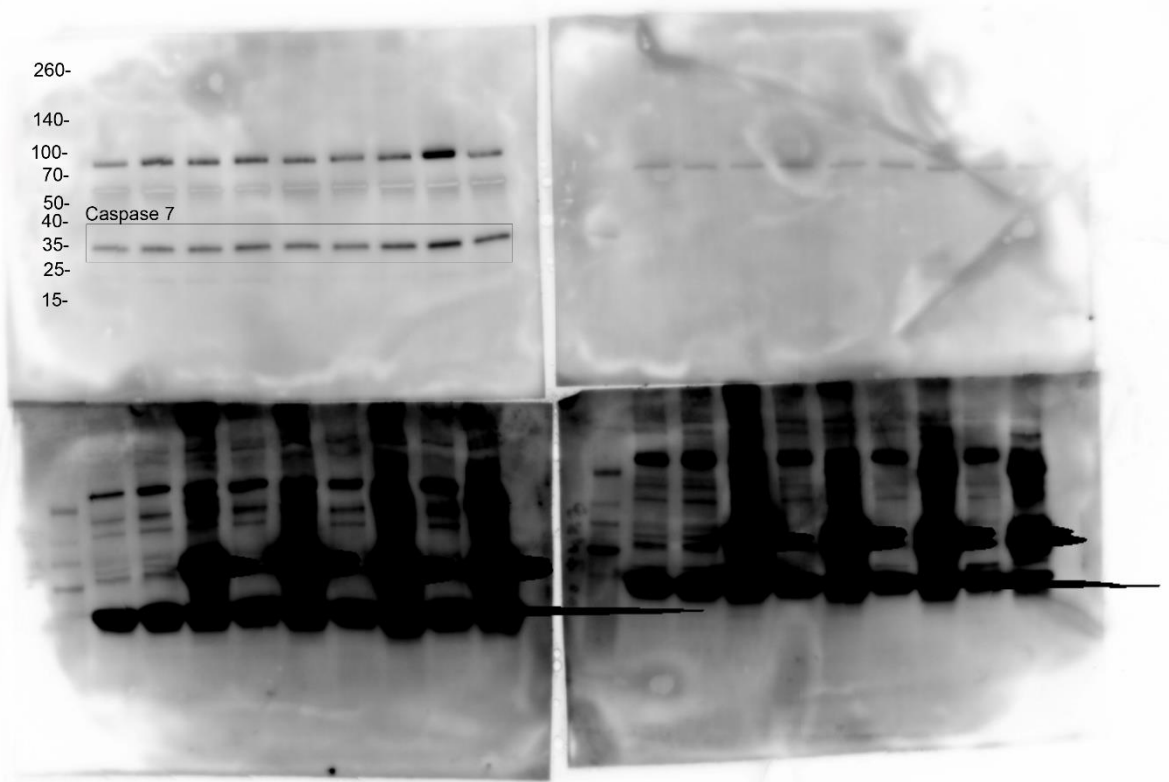

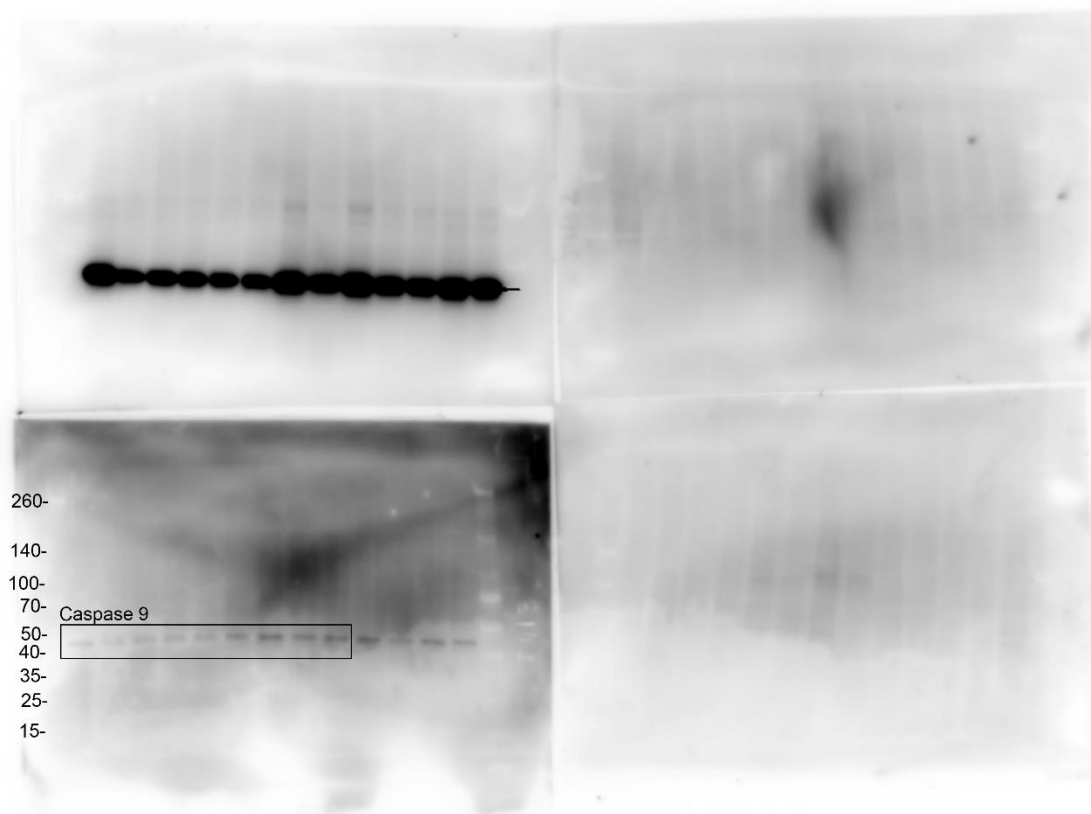

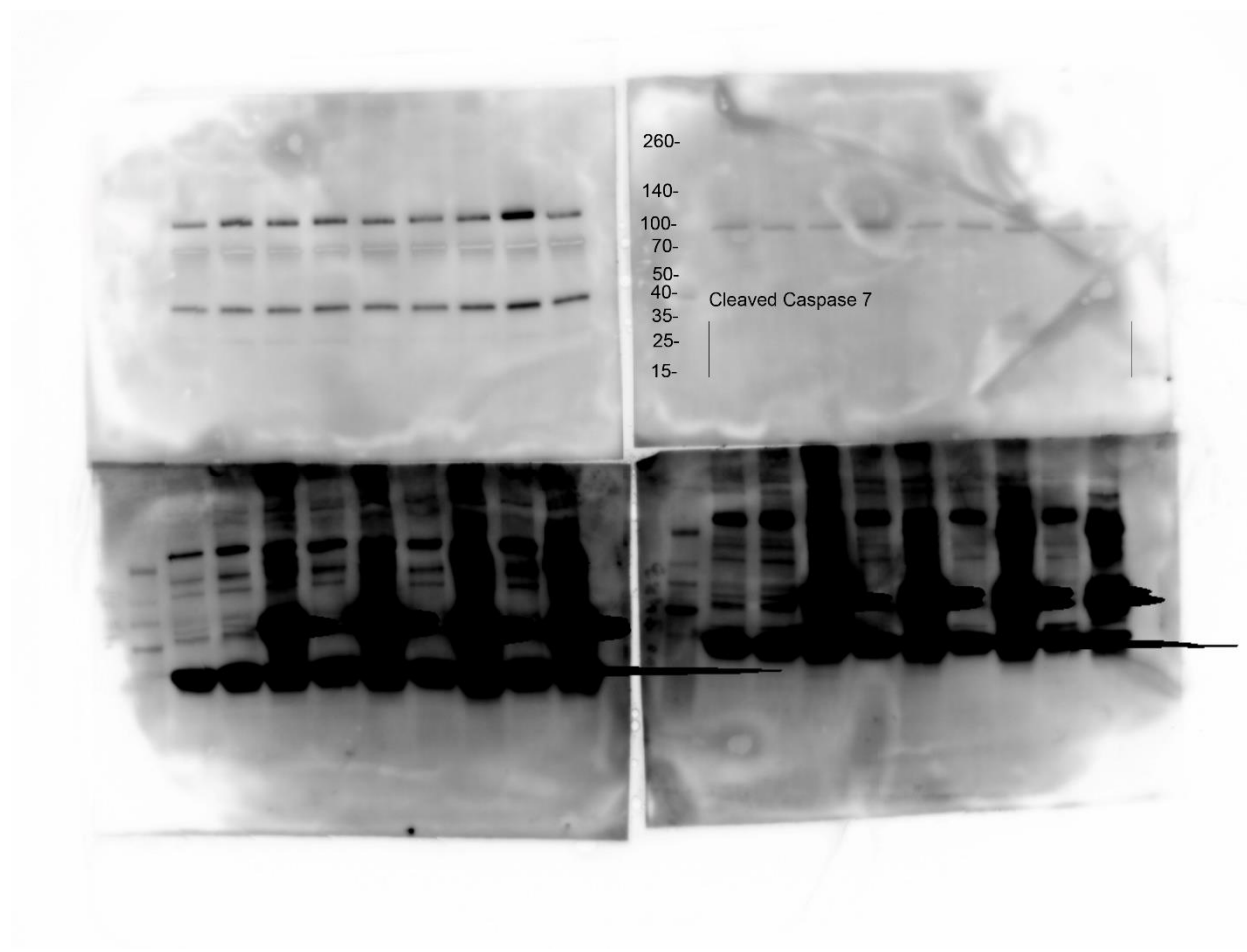

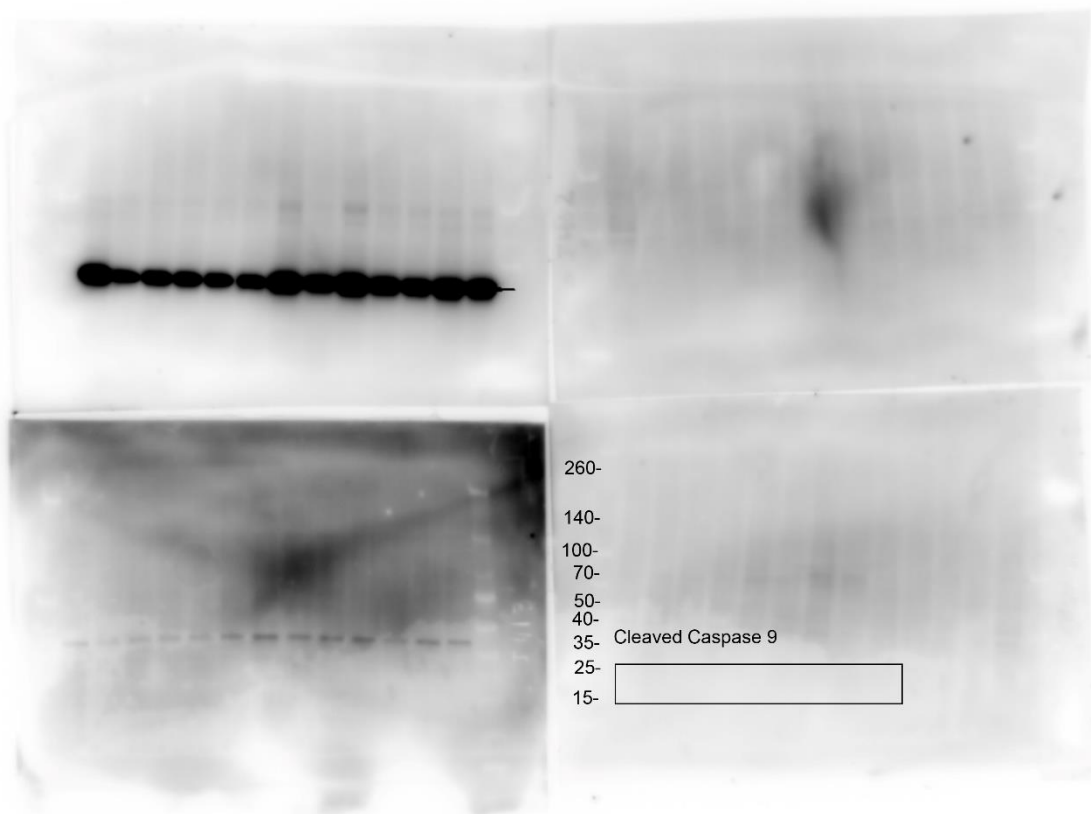

260-

140-

100-

70-

50-

40-

35-

25-

15- GAPDH

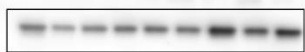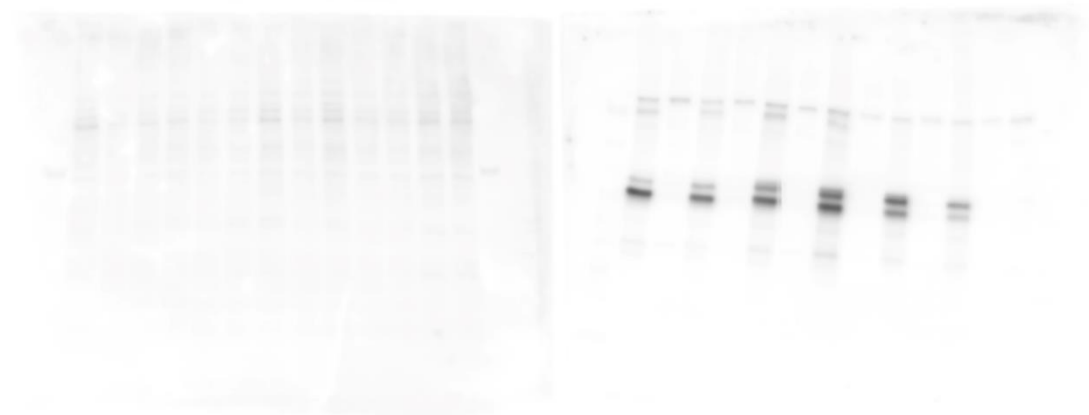

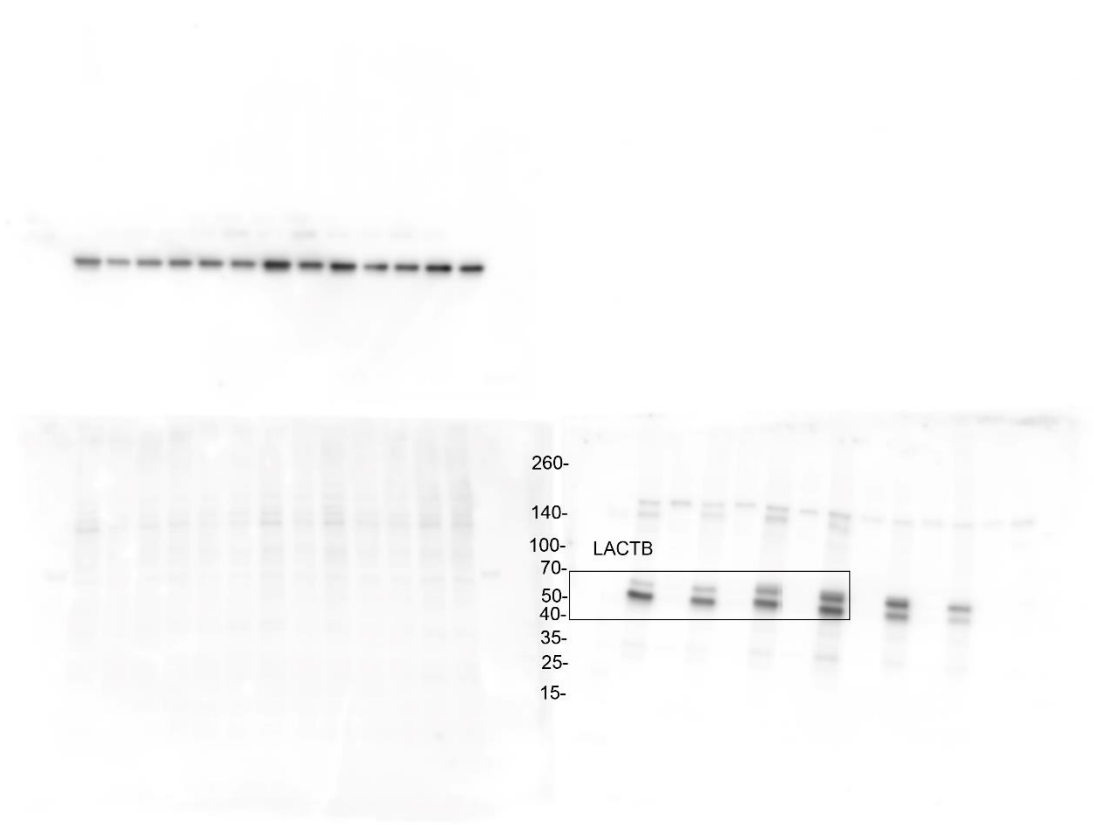

---

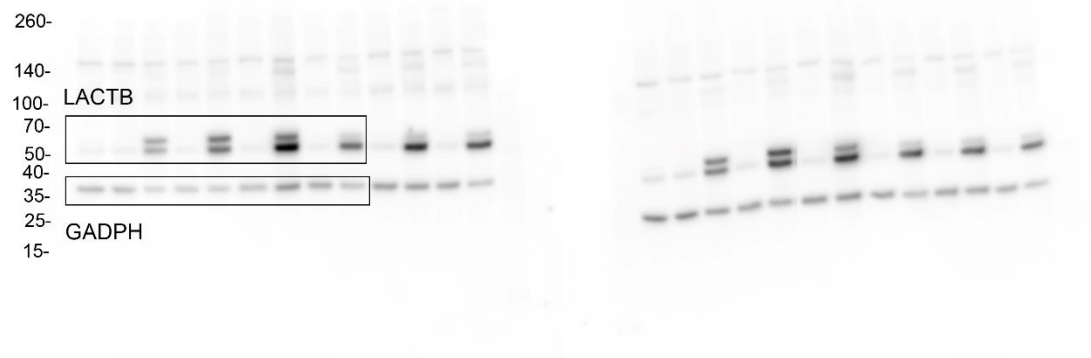

---

260-

140-

100-

70-

50-

40-

35-

25-

15-

Bid

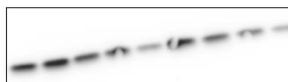

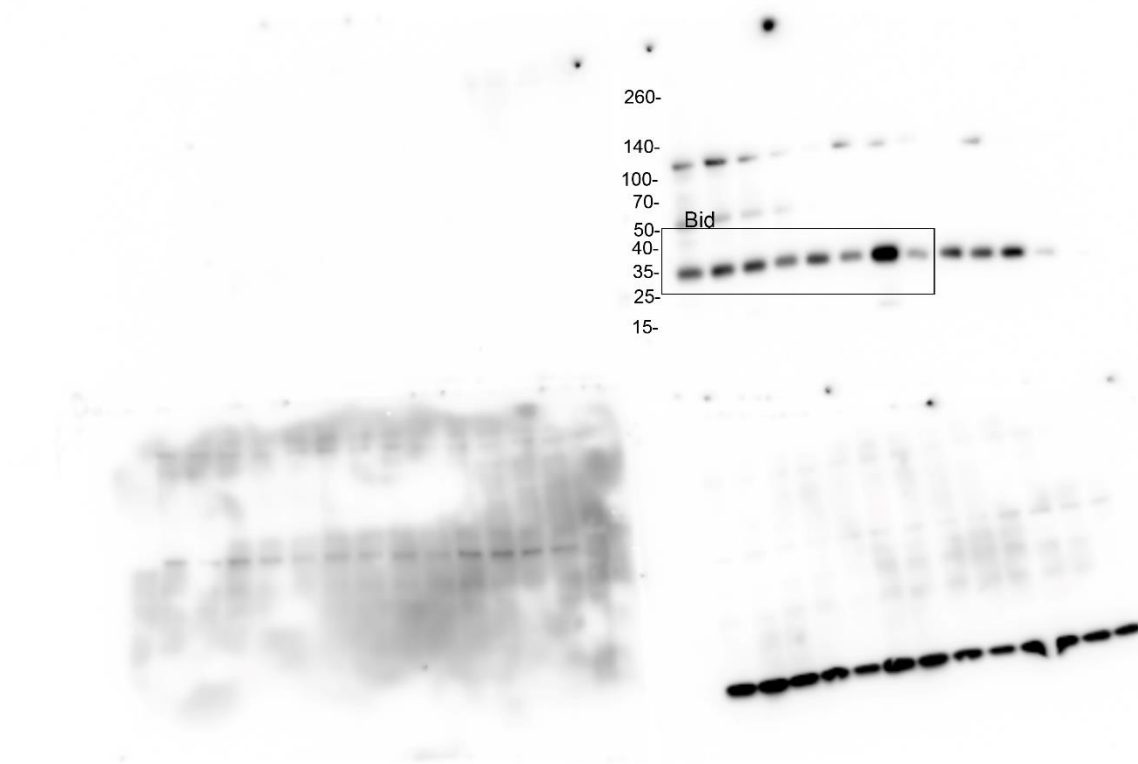

260-

140-

100-

70-

50-

40-

35-

25-

15-

Caspase 7

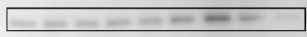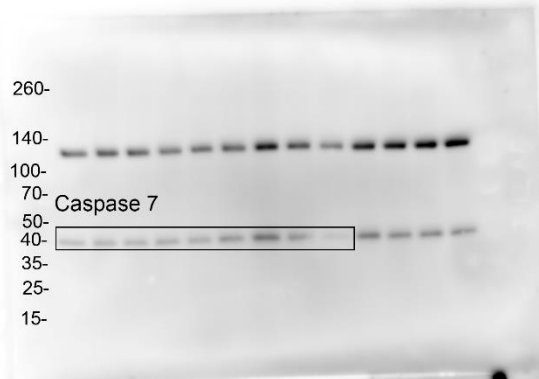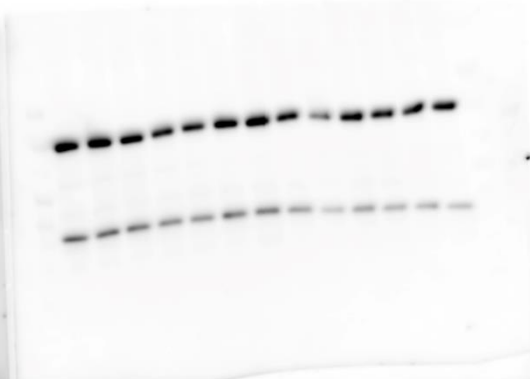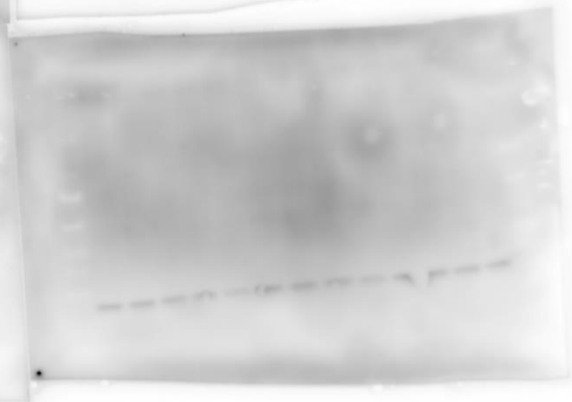

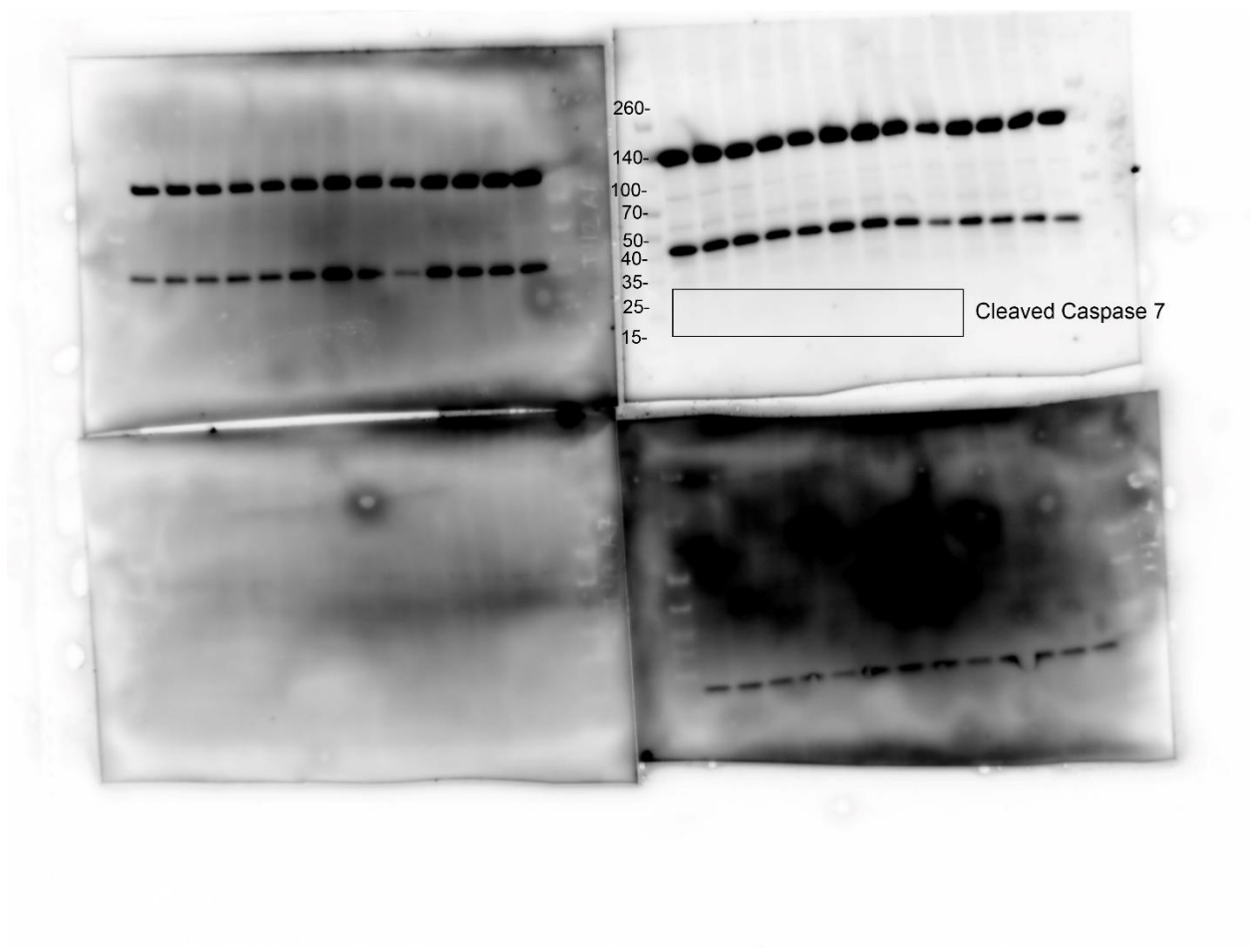

Supplement: Supplementary file 7 — Supplementary Material 7 [file 10495_2022_1775_MOESM7_ESM.pdf]

### Supplementary Figure 3 HCC1806

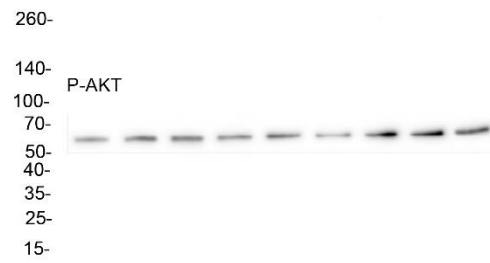

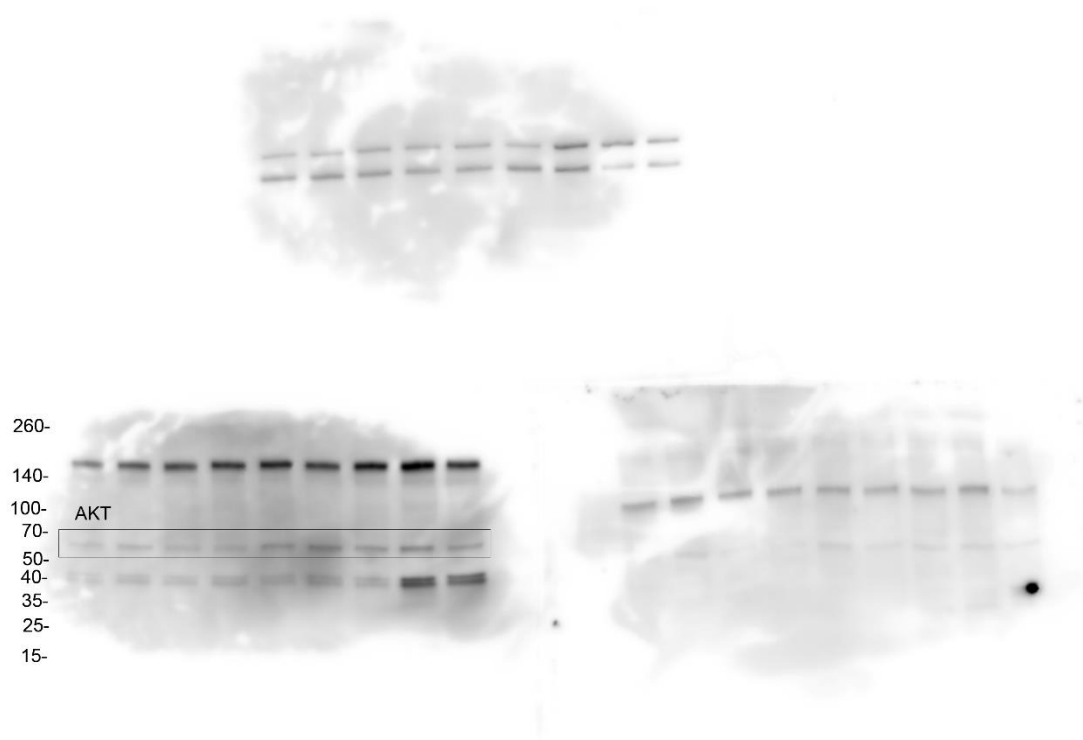

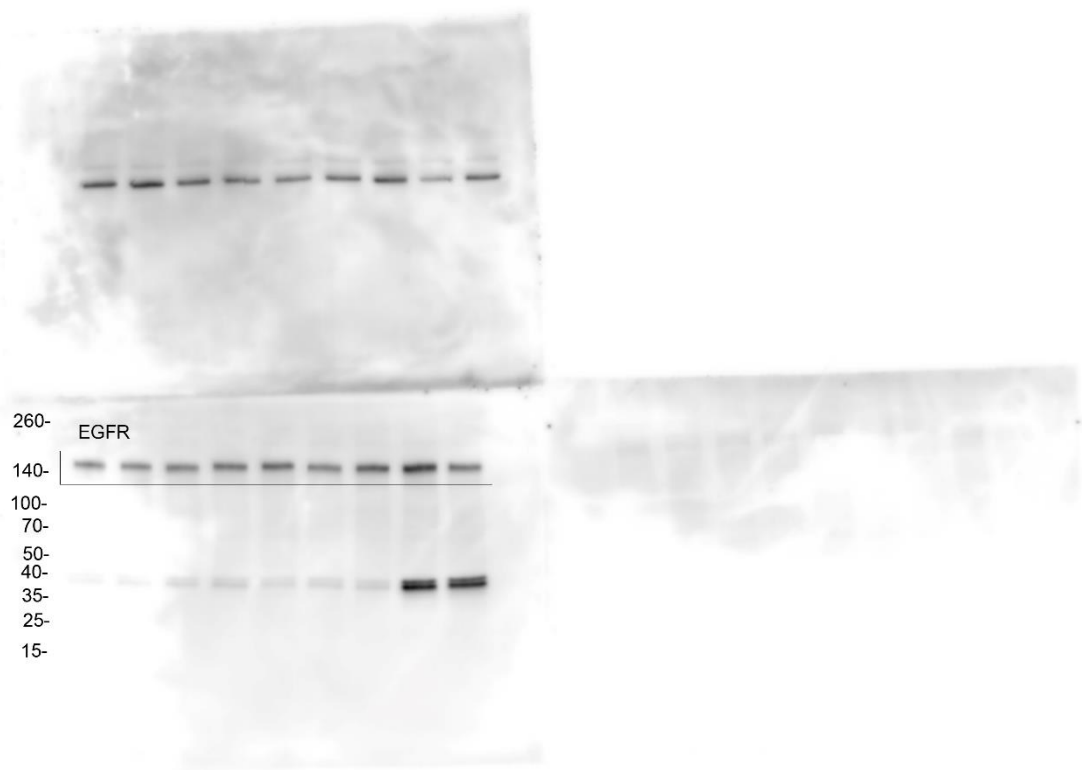

---

260-  
140-  
100-  
70-  
50-  
40-  
35-  
25-  
15-

GAPDH

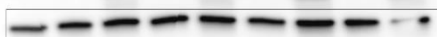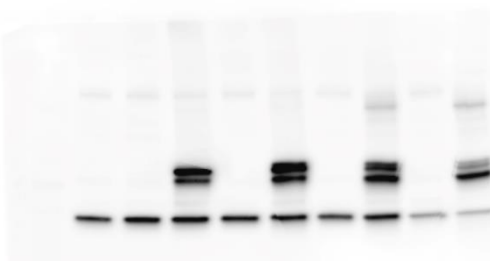

260-  
140-  
100-  
70-  
50-  
40-  
35-  
25-  
15-

LACTB

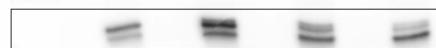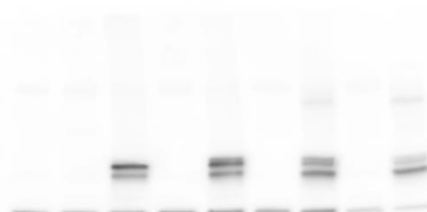

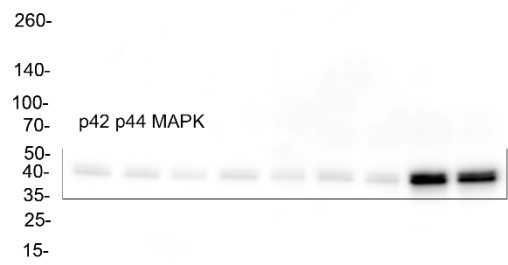

---

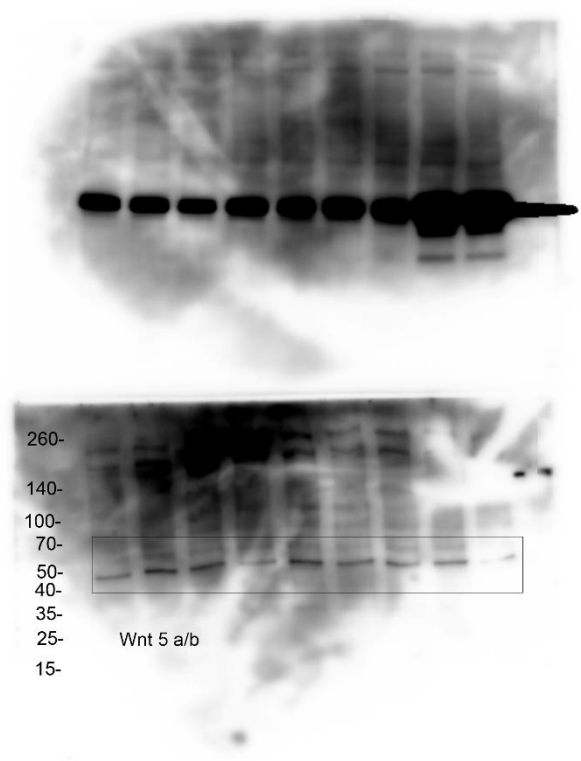

---

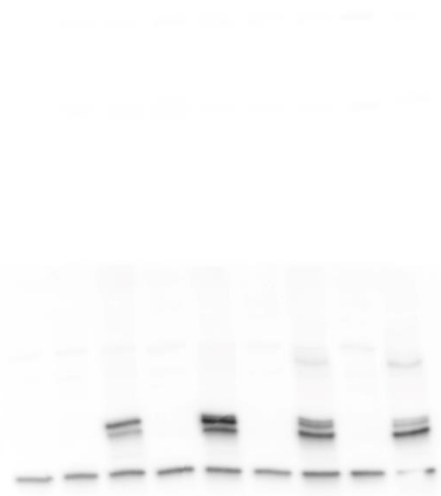

260-  
140-  
100-  
70-  
50-  
40-  
35-  
25-  
15-

GAPDH

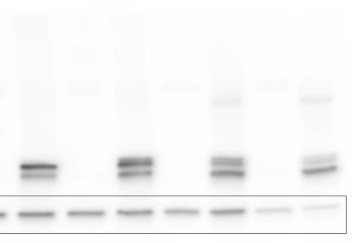

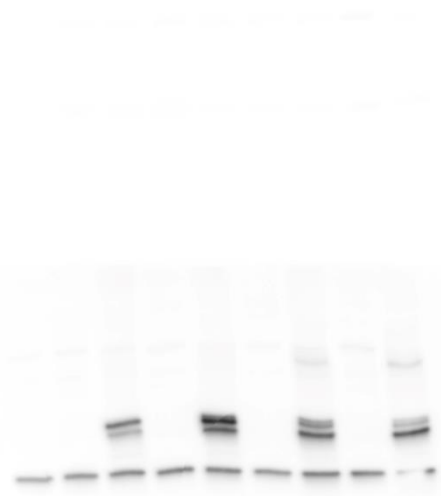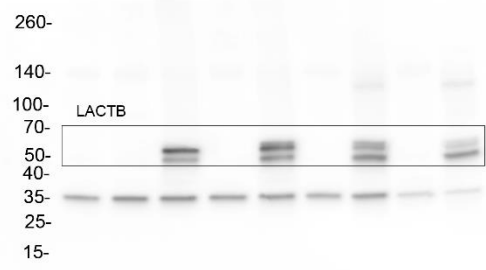

---

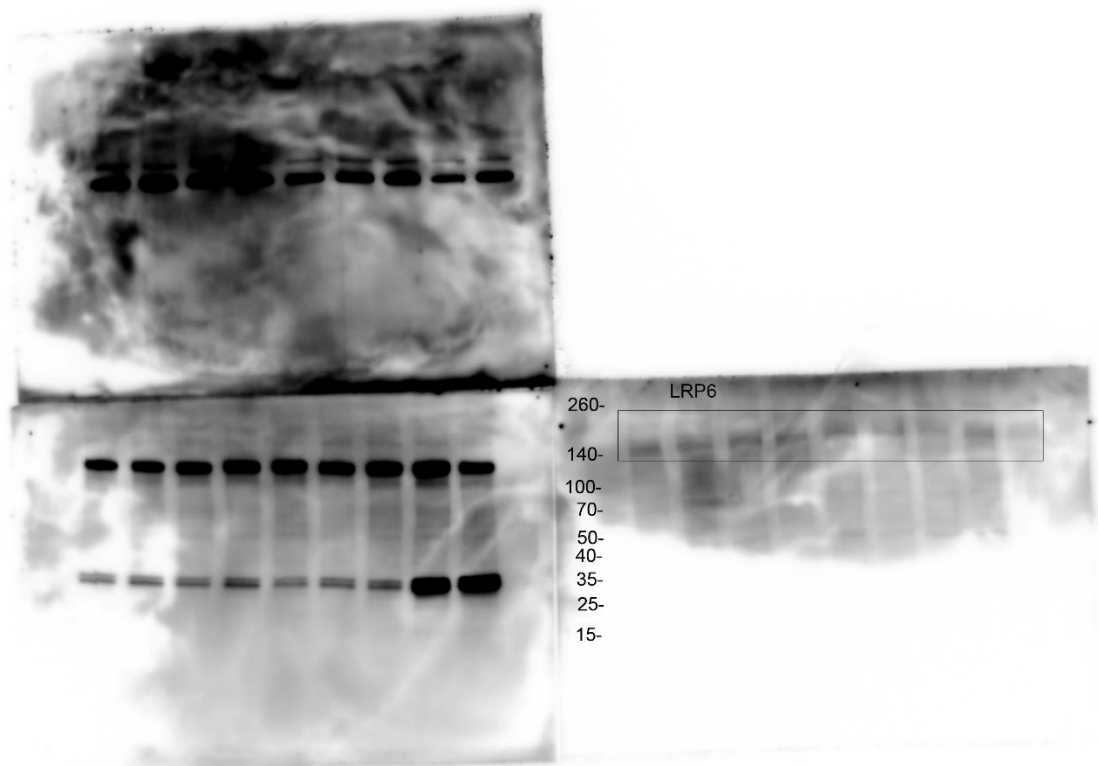

260-

140-

100- NFkB

70-

50-

40-

35-

25-

15-

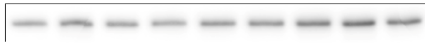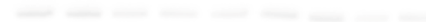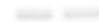

---

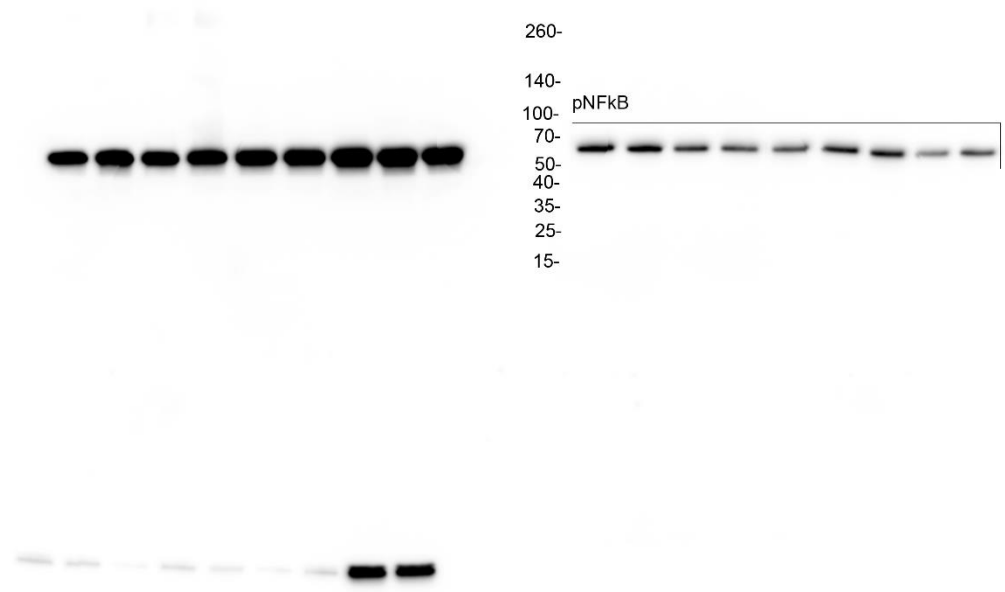

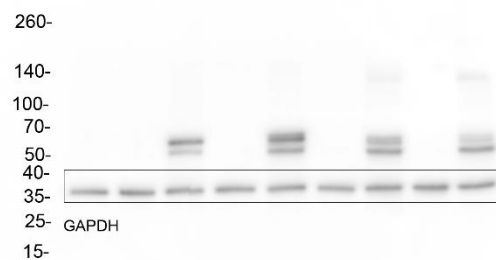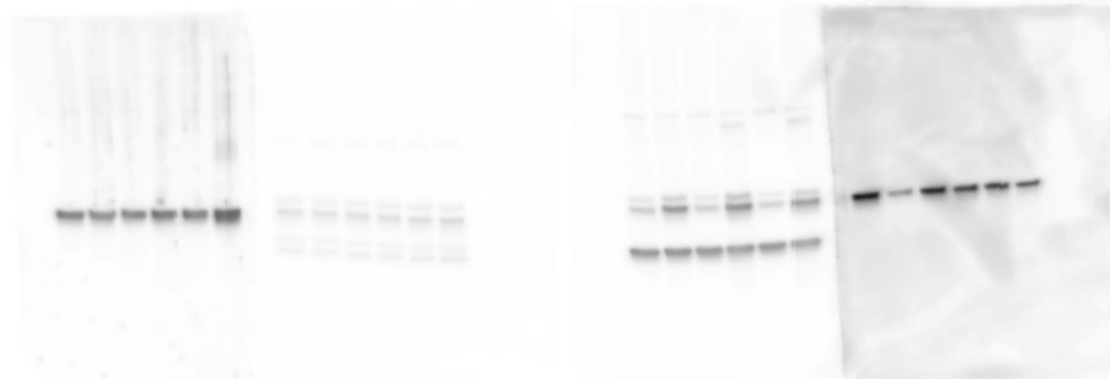

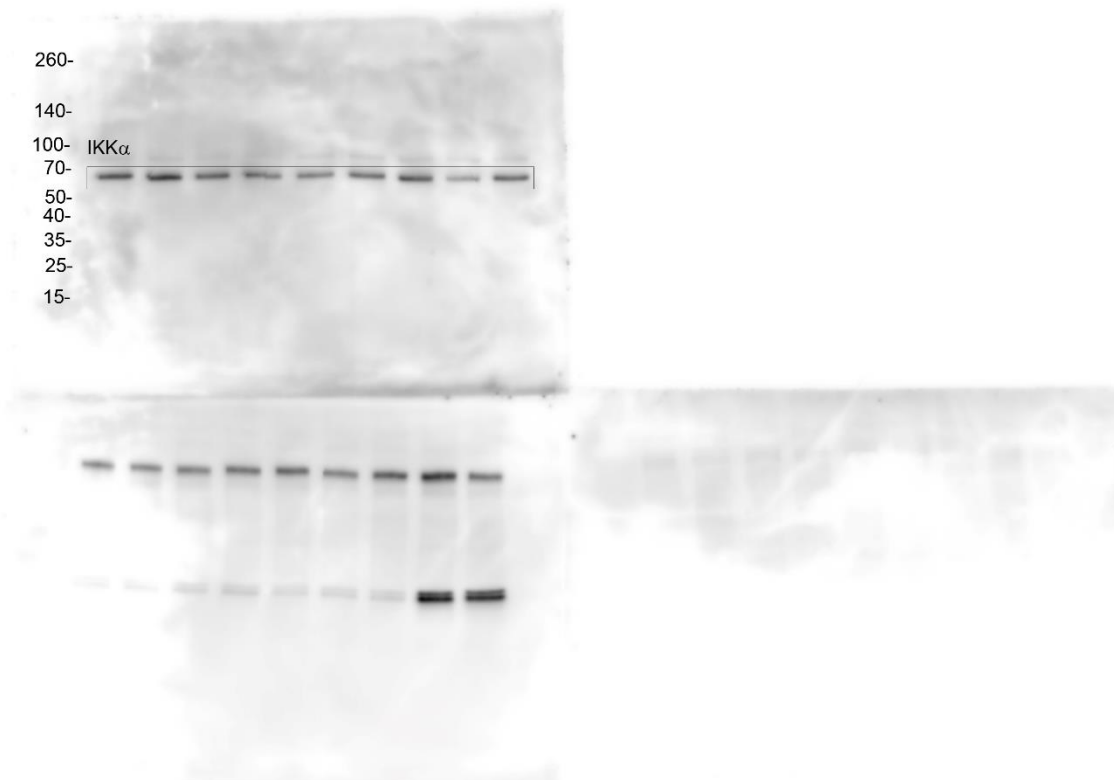

---

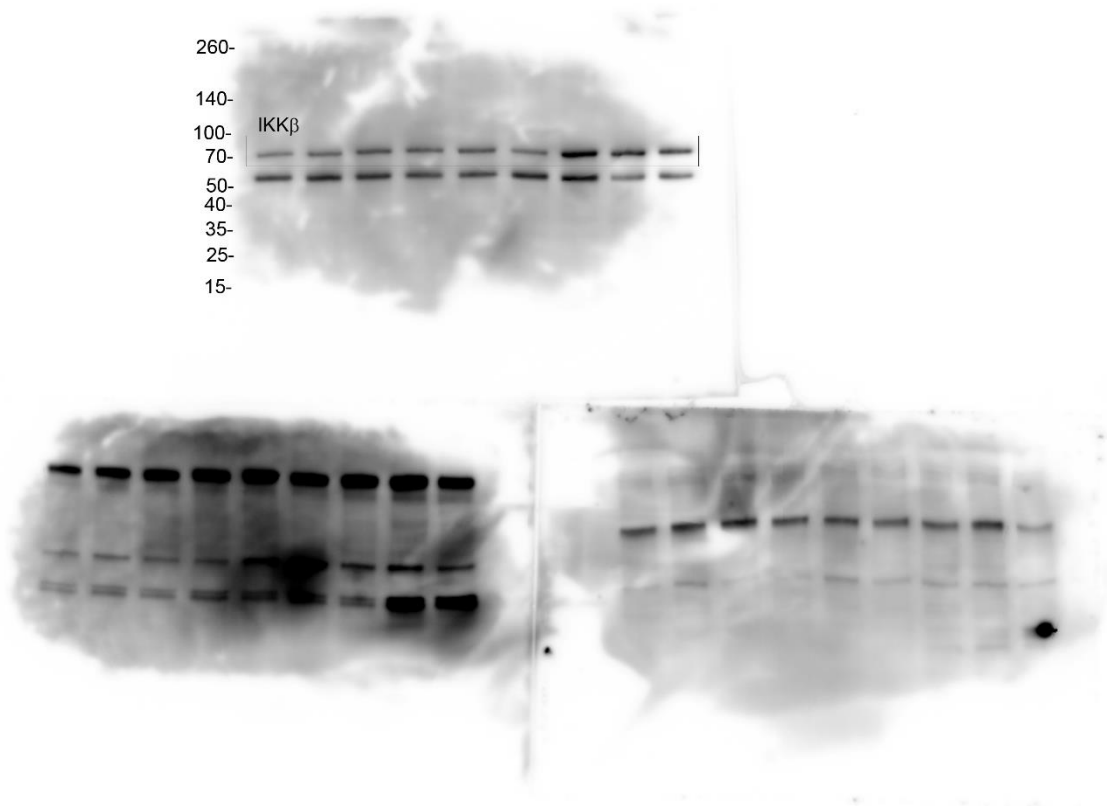

260-

140-

100- LACTB

70-

50-

40-

35-

25-

15-

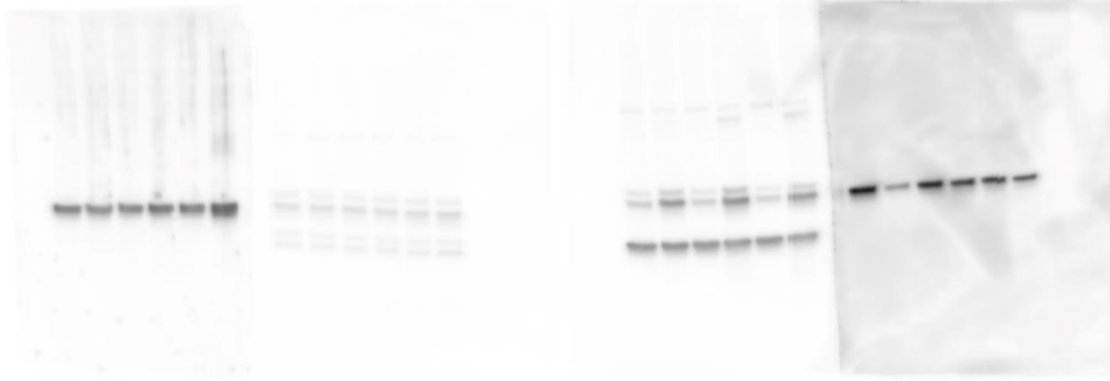

Supplement: Supplementary file 8 — Supplementary Material 8 [file 10495_2022_1775_MOESM8_ESM.pdf]

### Supplementary Figure 3 HS578t

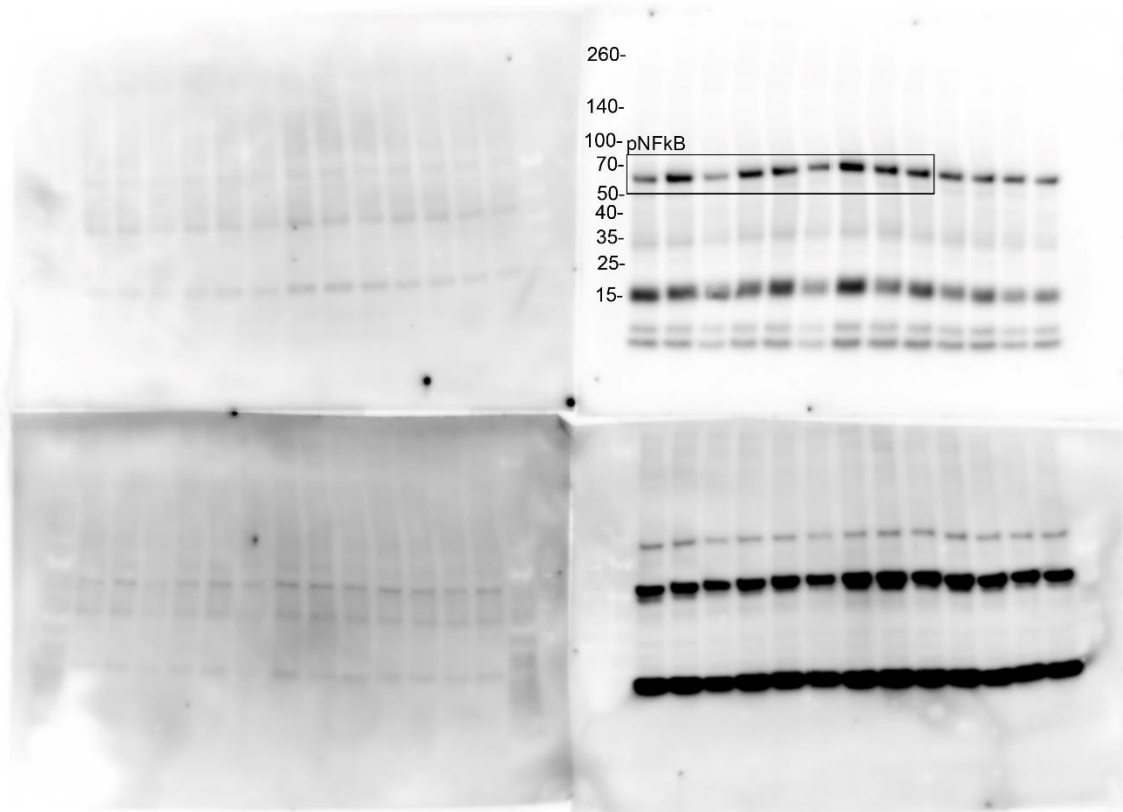

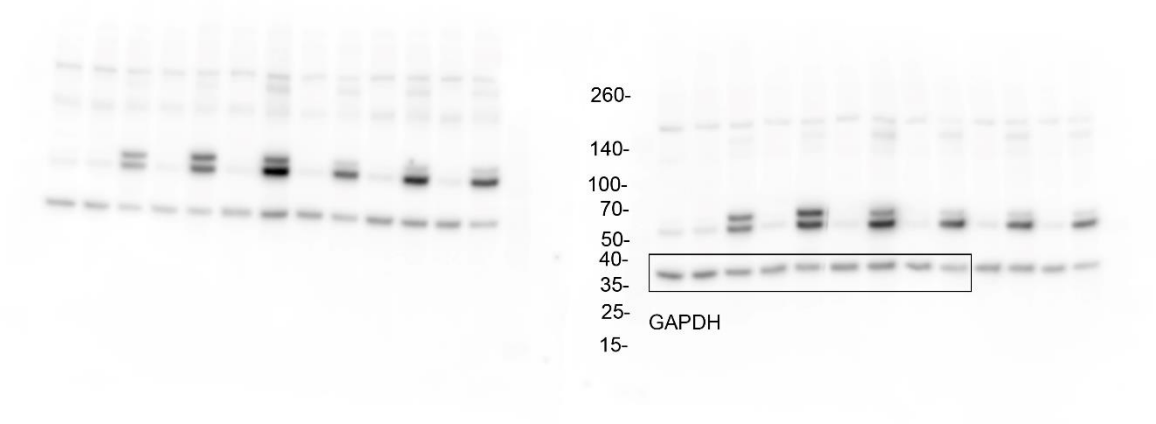

---

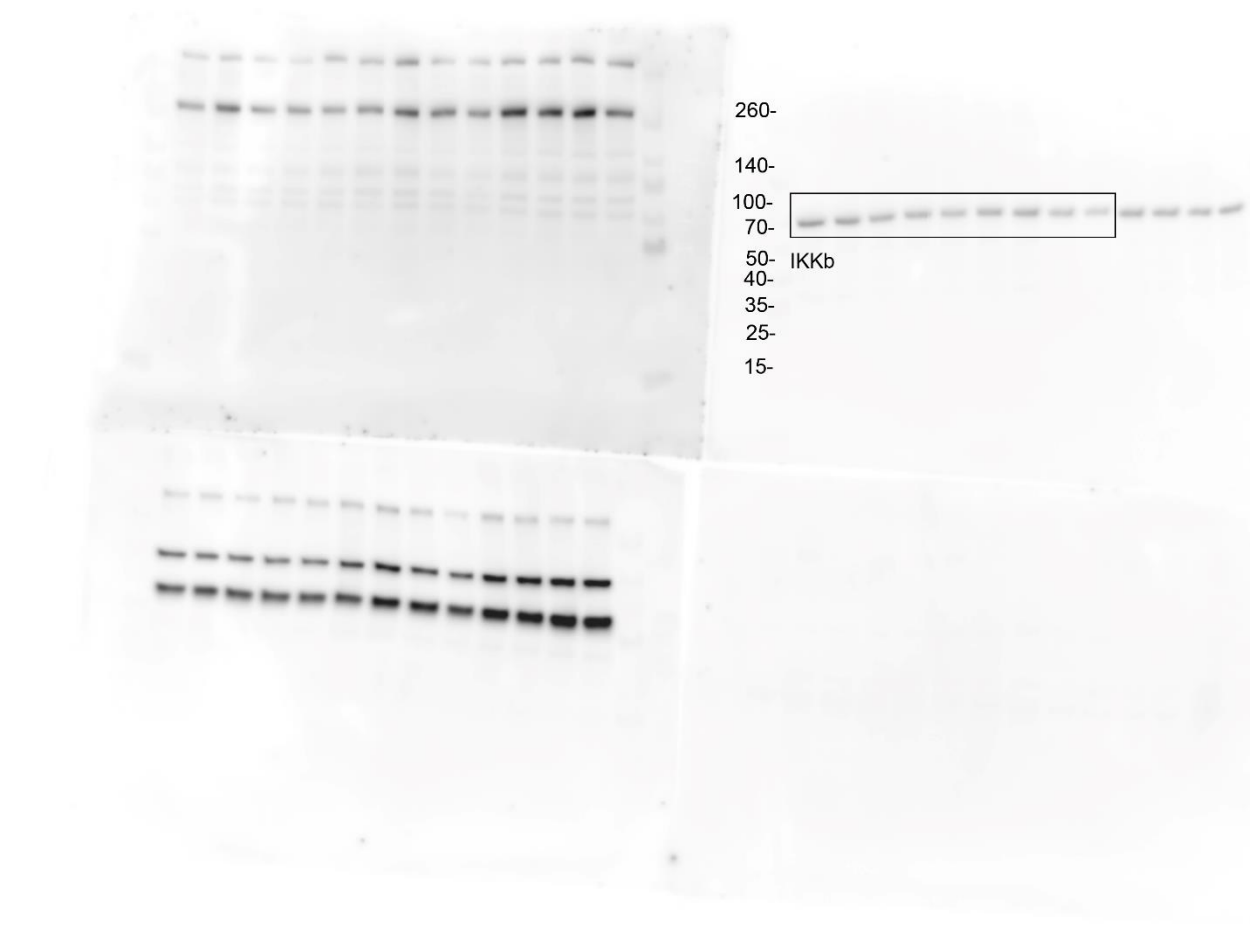

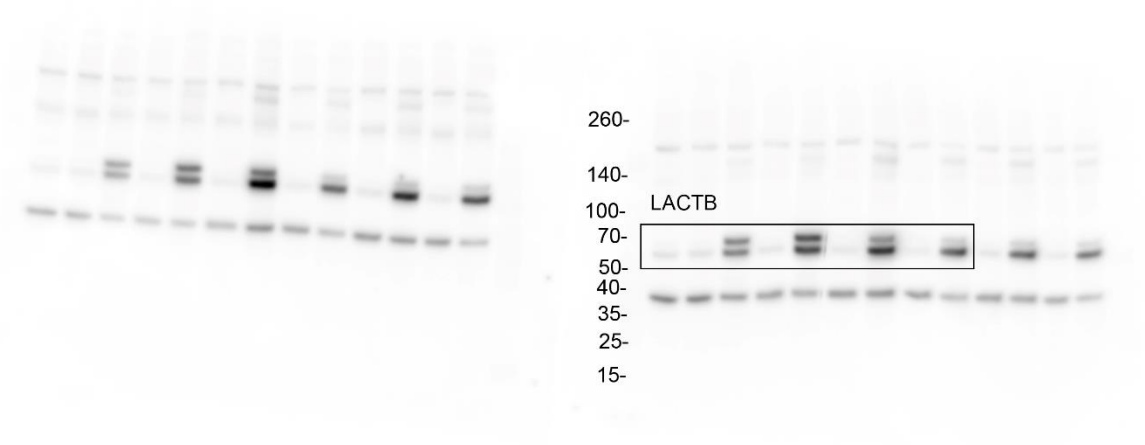

---

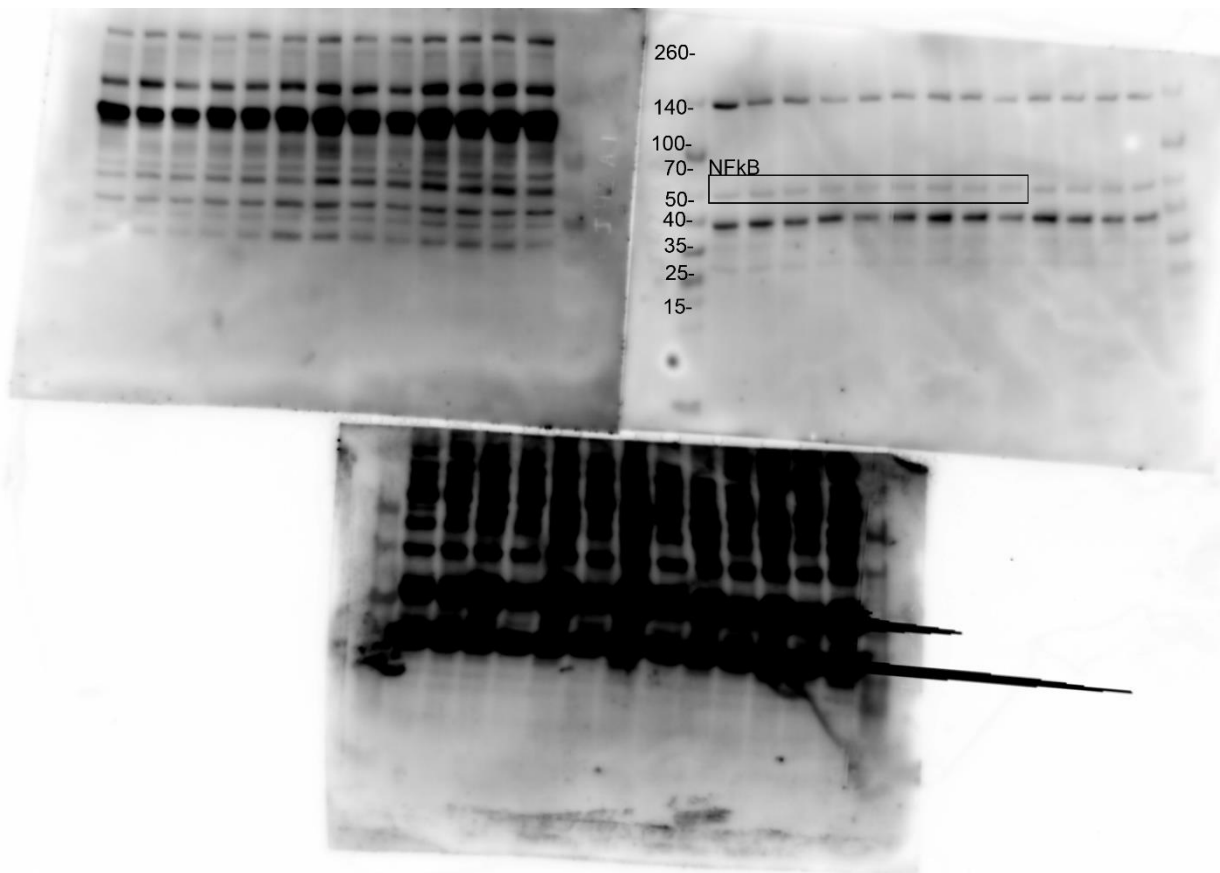

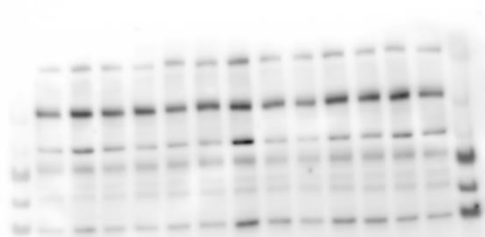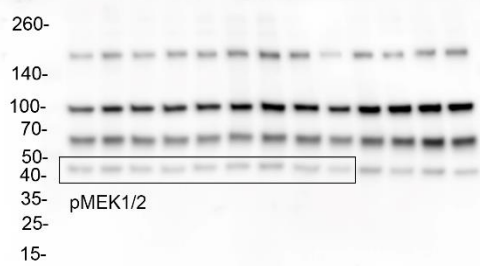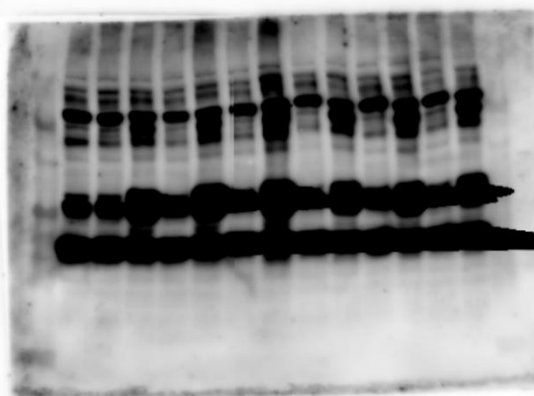

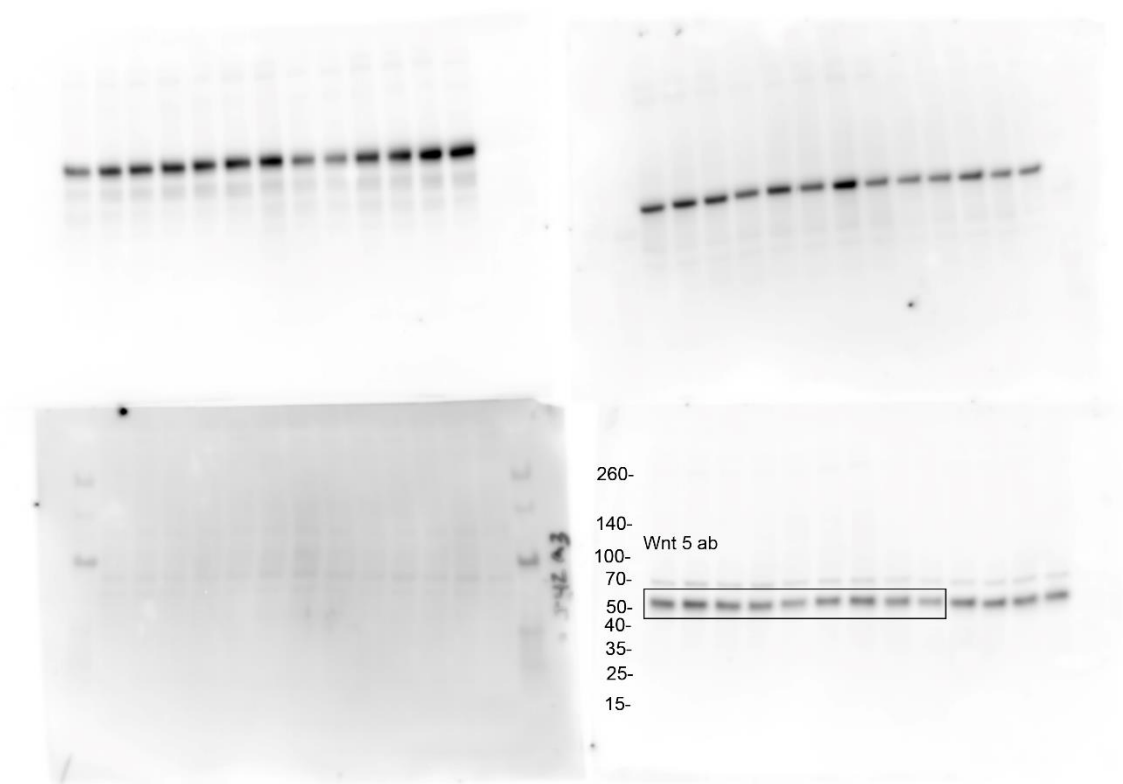

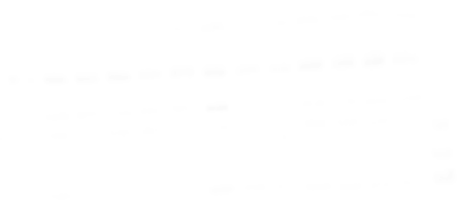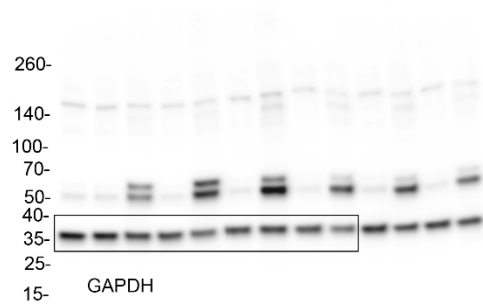

---

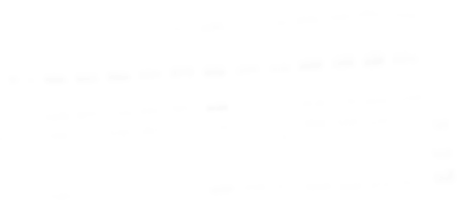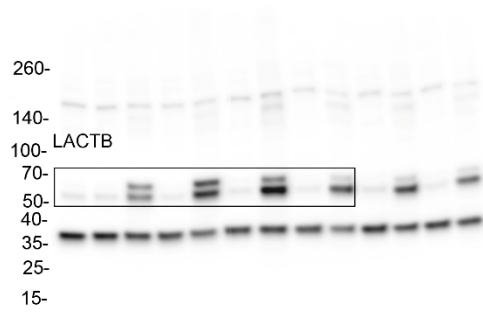

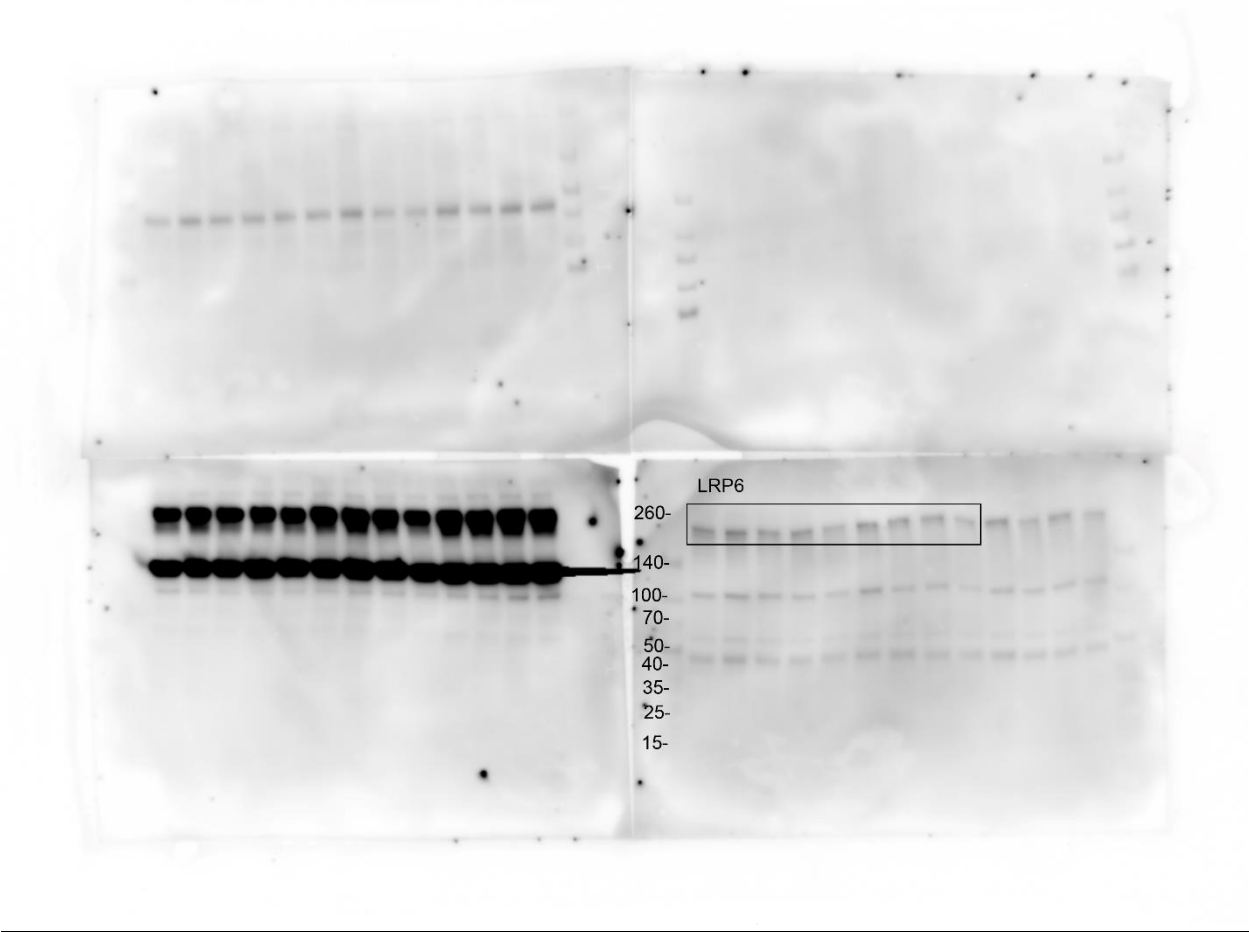

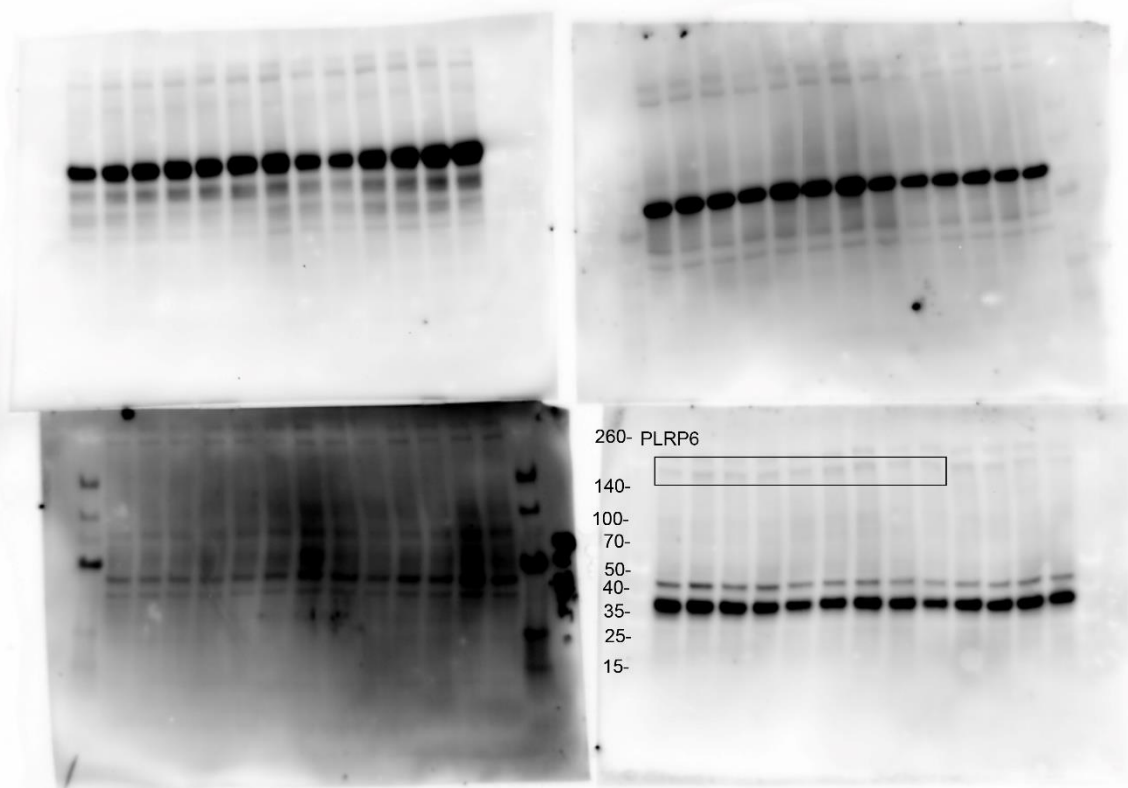

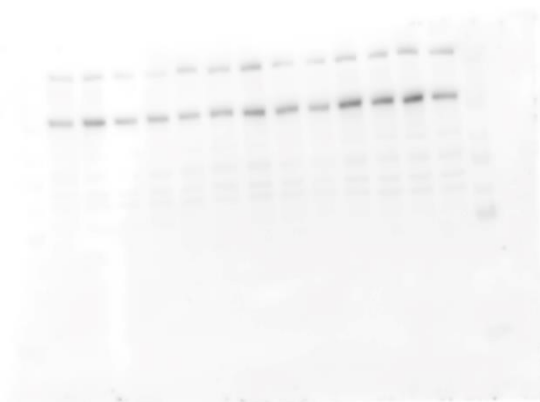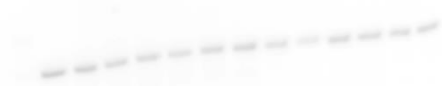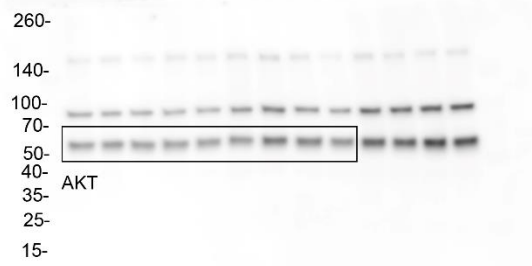

260-  
140-  
100-  
70-  
50-  
40-  
35-  
25-  
15-

EGFR

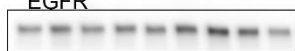

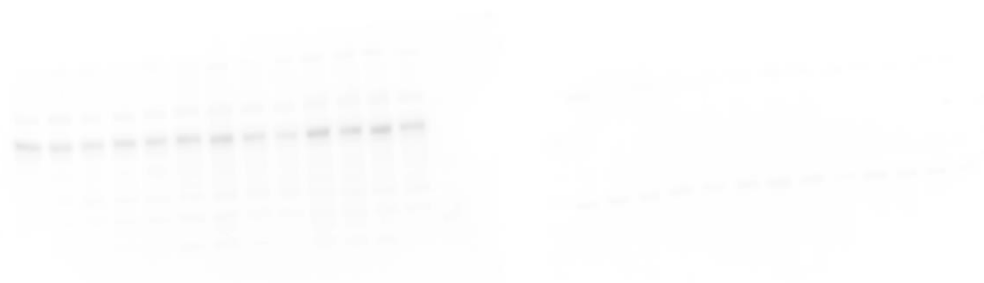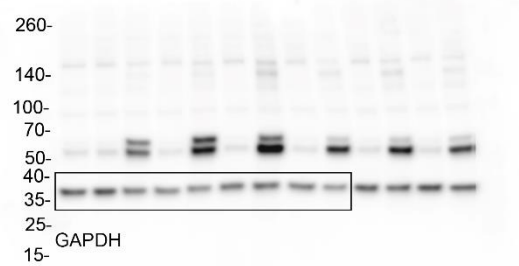

---

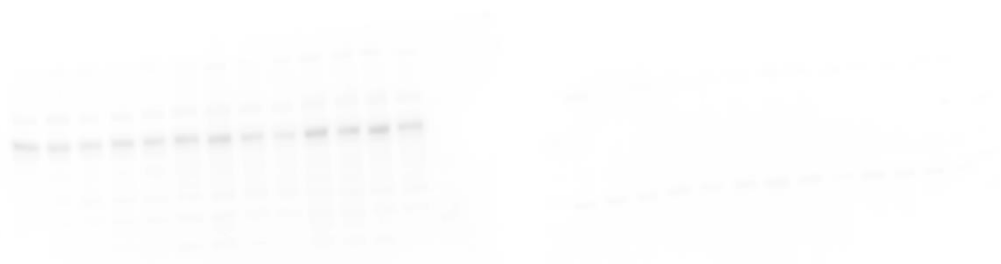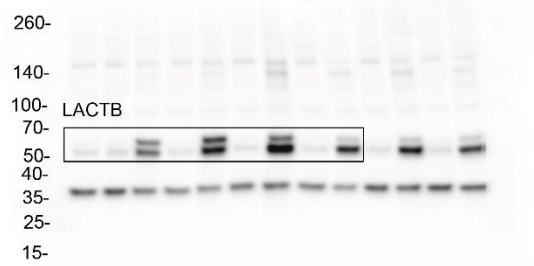

---

Supplement: Supplementary file 9 — Supplementary Material 9 [file 10495_2022_1775_MOESM9_ESM.pdf]

## Supplementary Figure 5

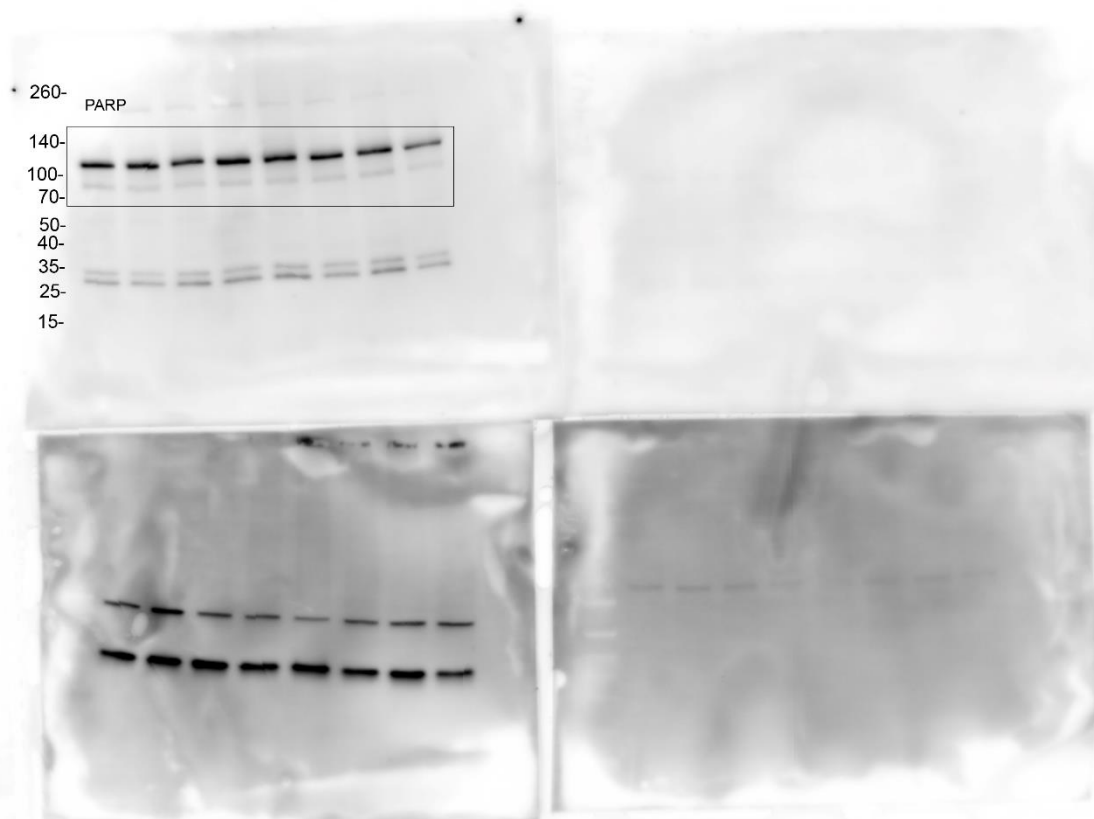

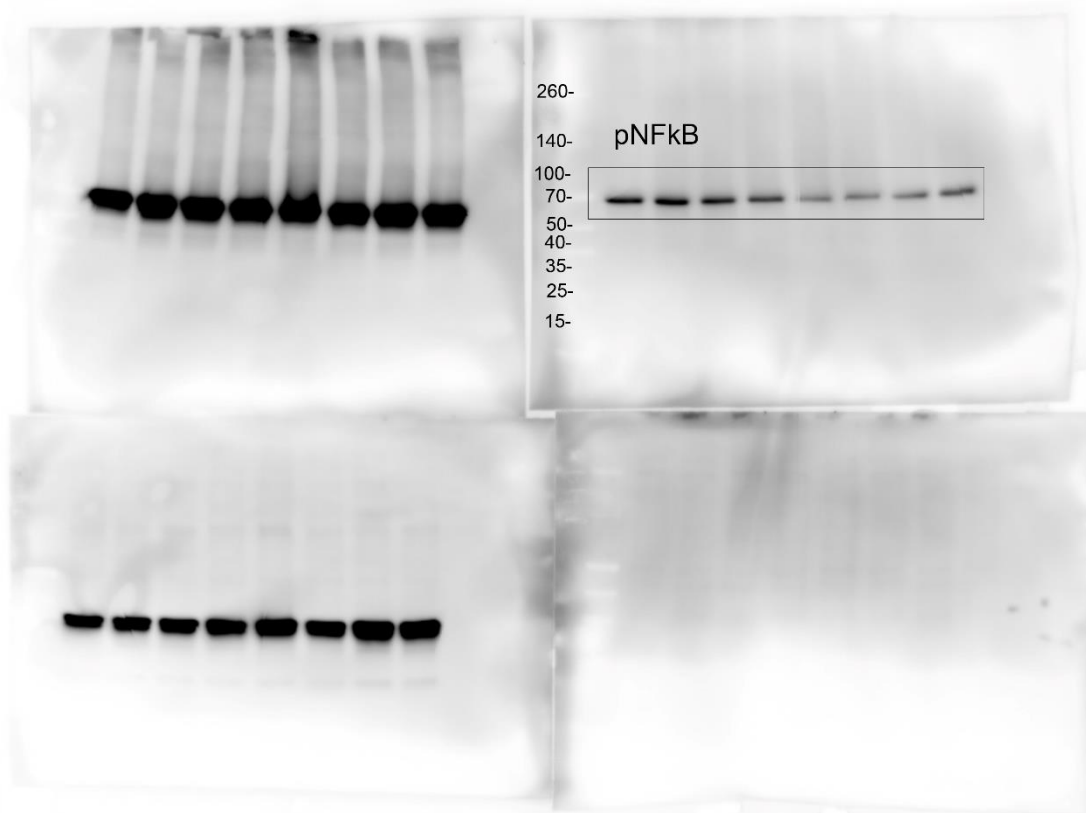

---

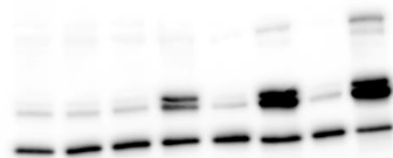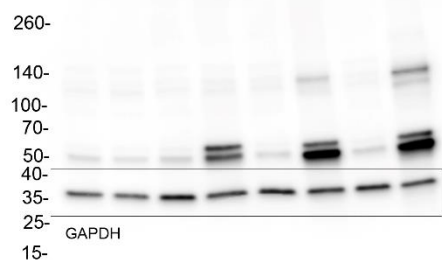

---

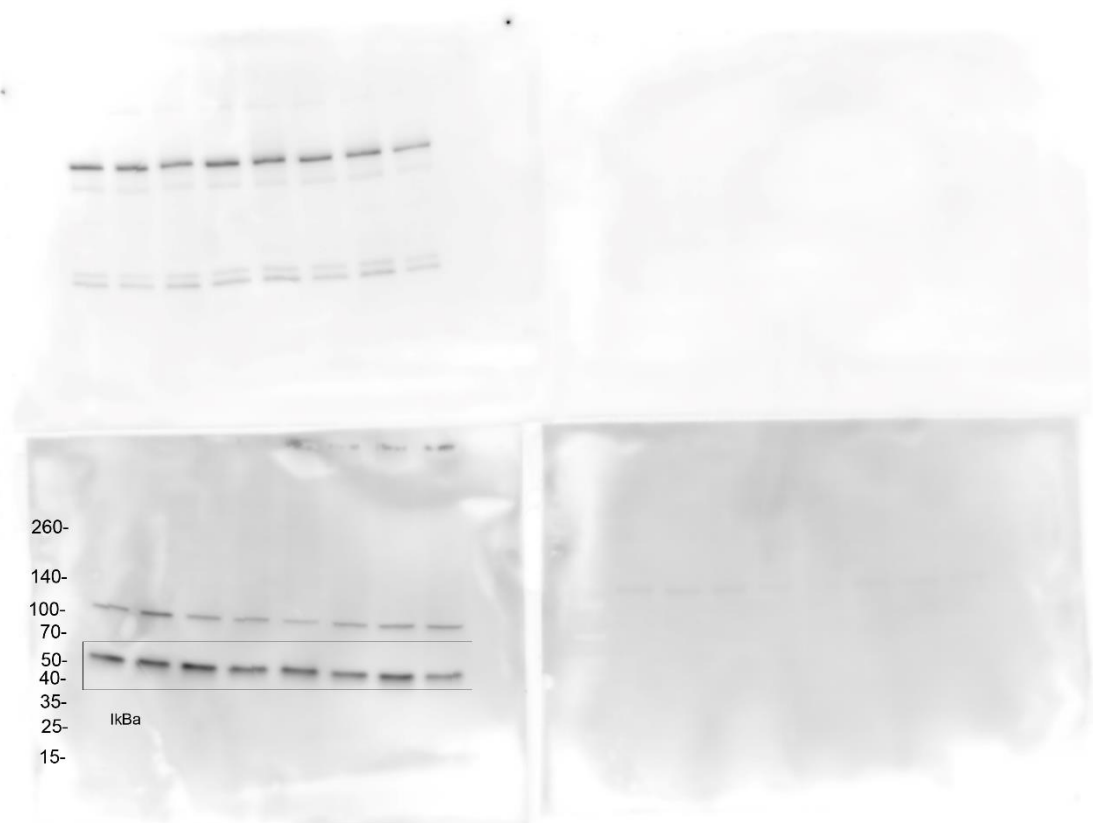

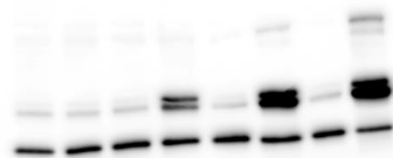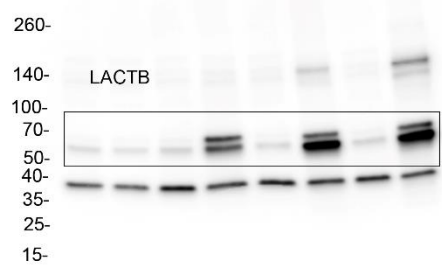

---

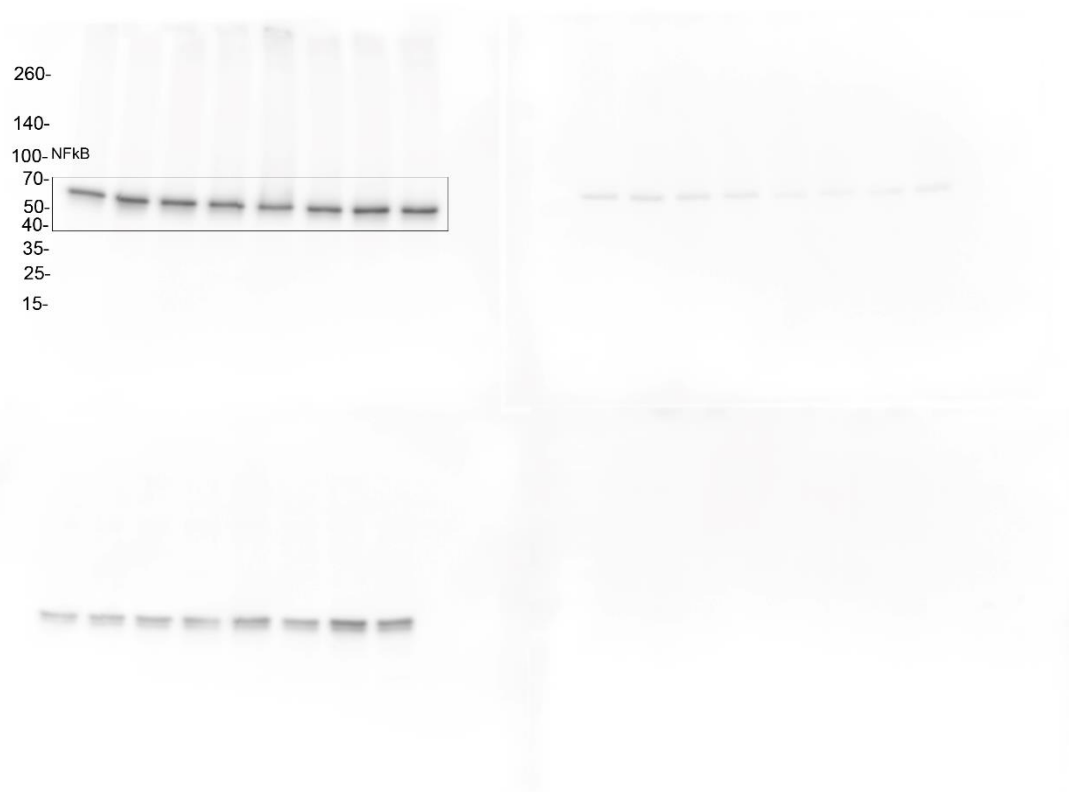

---

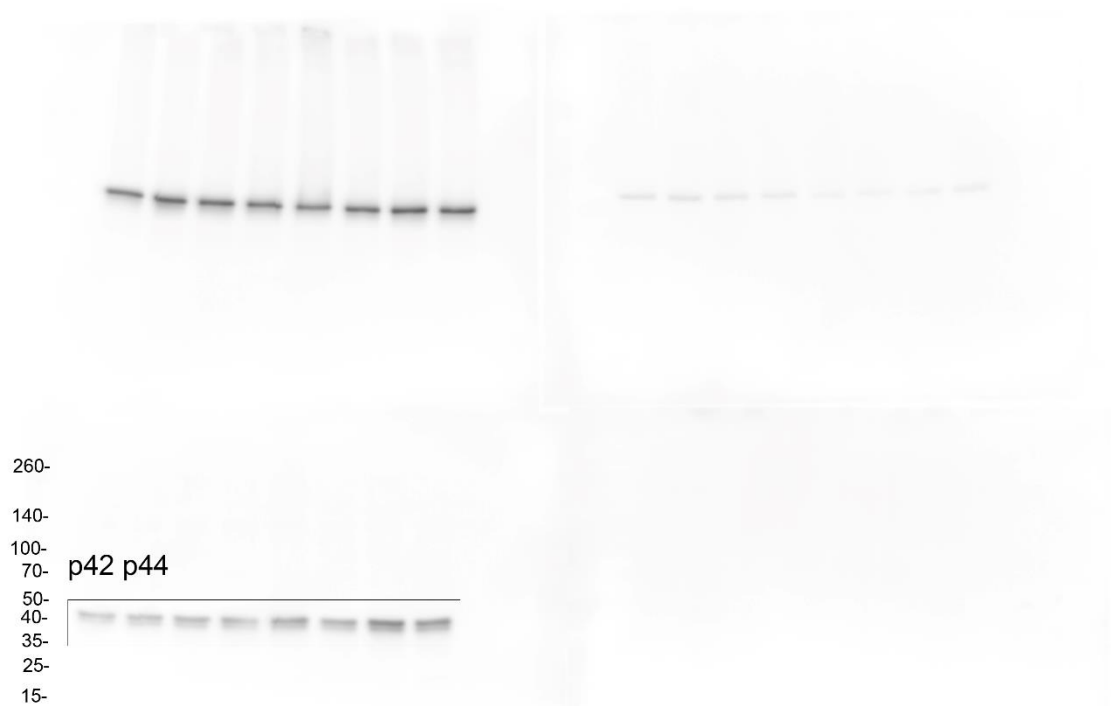

Supplement: Supplementary file 10 — Supplementary Material 10 [file 10495_2022_1775_MOESM10_ESM.pdf]
