# Supplementary material for: LACTB induces cancer cell death through the activation of the intrinsic caspase-independent pathway in breast cancer
Source: Apoptosis. 2022 Oct 25;28(1-2):186–98. doi: 10.1007/s10495-022-01775-4 (PMC9950249; doi:10.1007/s10495-022-01775-4)
Supplement: Supplementary file 11 — Supplementary Material 11 [file 10495_2022_1775_MOESM11_ESM.docx]

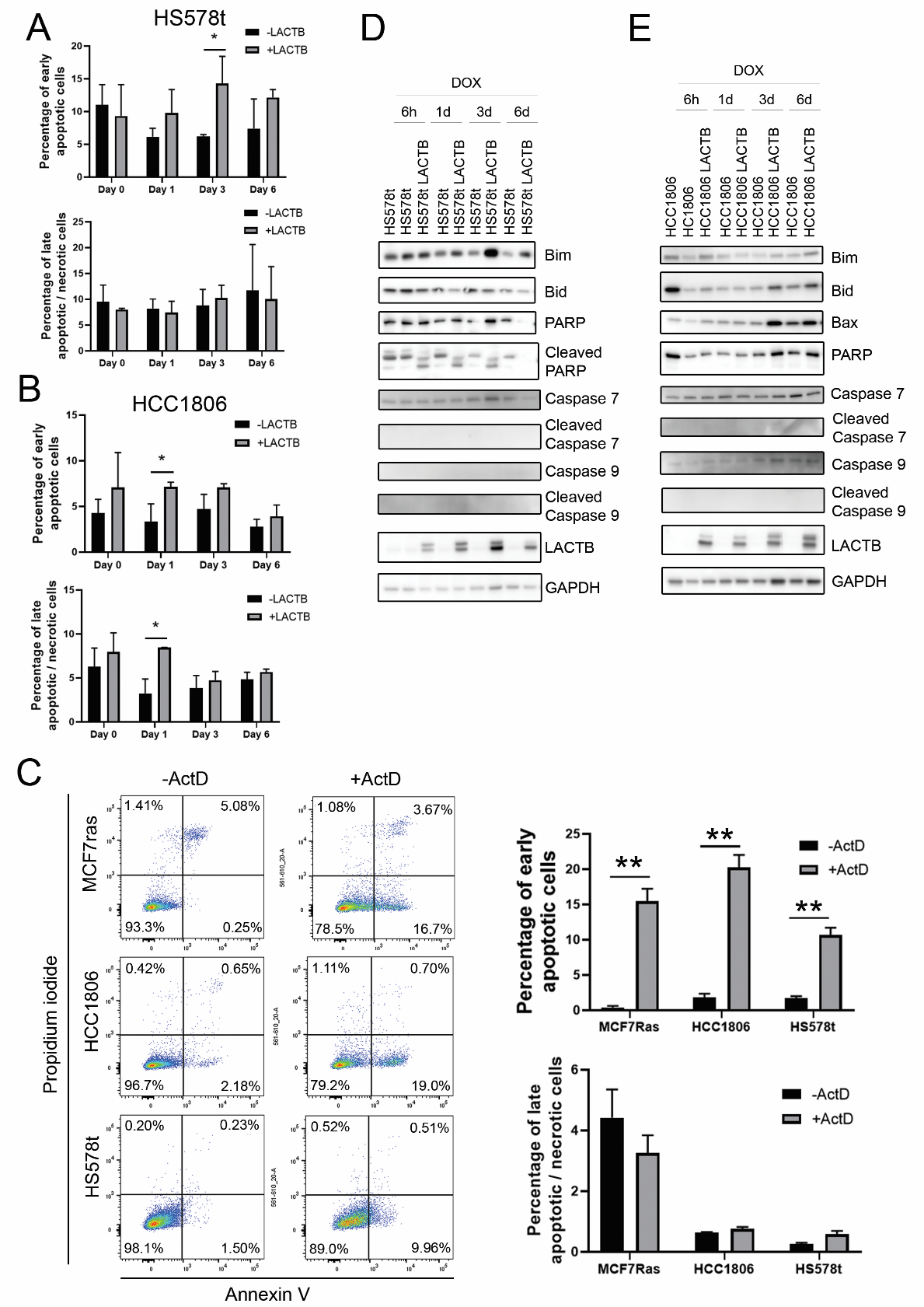


***Supplementary Figure 2: LACTB induces apoptosis in other breast cancer cell lines.*** Annexin V/PI analysis by flow cytometry of HS578t (A) and HCC1806 cells (B). LACTB was induced for the indicated time points and percentage of early apoptotic cells and late apoptotic/necrotic cells were measured by flow cytometry. Values of apoptotic ratios for each condition are presented as the mean ± standard deviation. * P<0.05 vs. control group (-LACTB). (C) Induction of apoptosis by 15nM Actinomycin D for 24 hours in different breast cancer cell lines. Levels of apoptosis were measured by FACS using Annexin V/PI. Percentage of early apoptotic cells and late apoptotic/necrotic cells were measured by flow cytometry. Values of apoptotic ratios for each condition are presented as the mean ± standard deviation. **P<0,01. Western blot analysis of apoptotic pathways in HS578t (D) and HCC1806 (E).
