# Supplementary material for: LACTB induces cancer cell death through the activation of the intrinsic caspase-independent pathway in breast cancer
Source: Apoptosis. 2022 Oct 25;28(1-2):186–98. doi: 10.1007/s10495-022-01775-4 (PMC9950249; doi:10.1007/s10495-022-01775-4)
Supplement: Supplementary file 12 — Supplementary Material 12 [file 10495_2022_1775_MOESM12_ESM.docx]

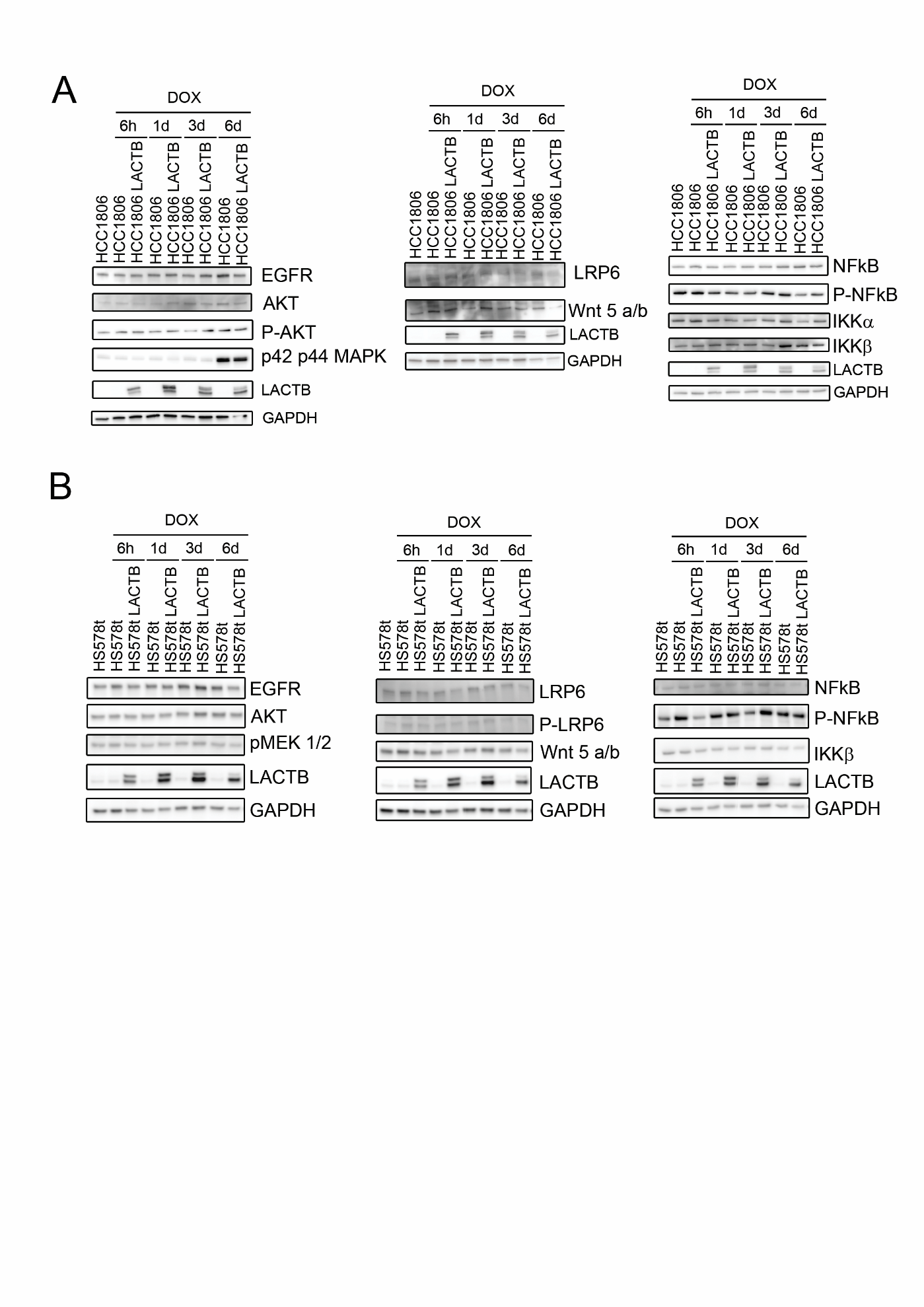


***Supplementary Figure 4: LACTB affects proliferation pathways in other breast cancer cell lines.*** Western blot of pro-survival pathways in HCC1806 (A) and HS578t (B). LACTB was induced with doxycycline for the indicated time points.
