# Supplementary material for: LACTB induces cancer cell death through the activation of the intrinsic caspase-independent pathway in breast cancer
Source: Apoptosis. 2022 Oct 25;28(1-2):186–98. doi: 10.1007/s10495-022-01775-4 (PMC9950249; doi:10.1007/s10495-022-01775-4)
Supplement: Supplementary file 13 — Supplementary Material 13 [file 10495_2022_1775_MOESM13_ESM.docx]

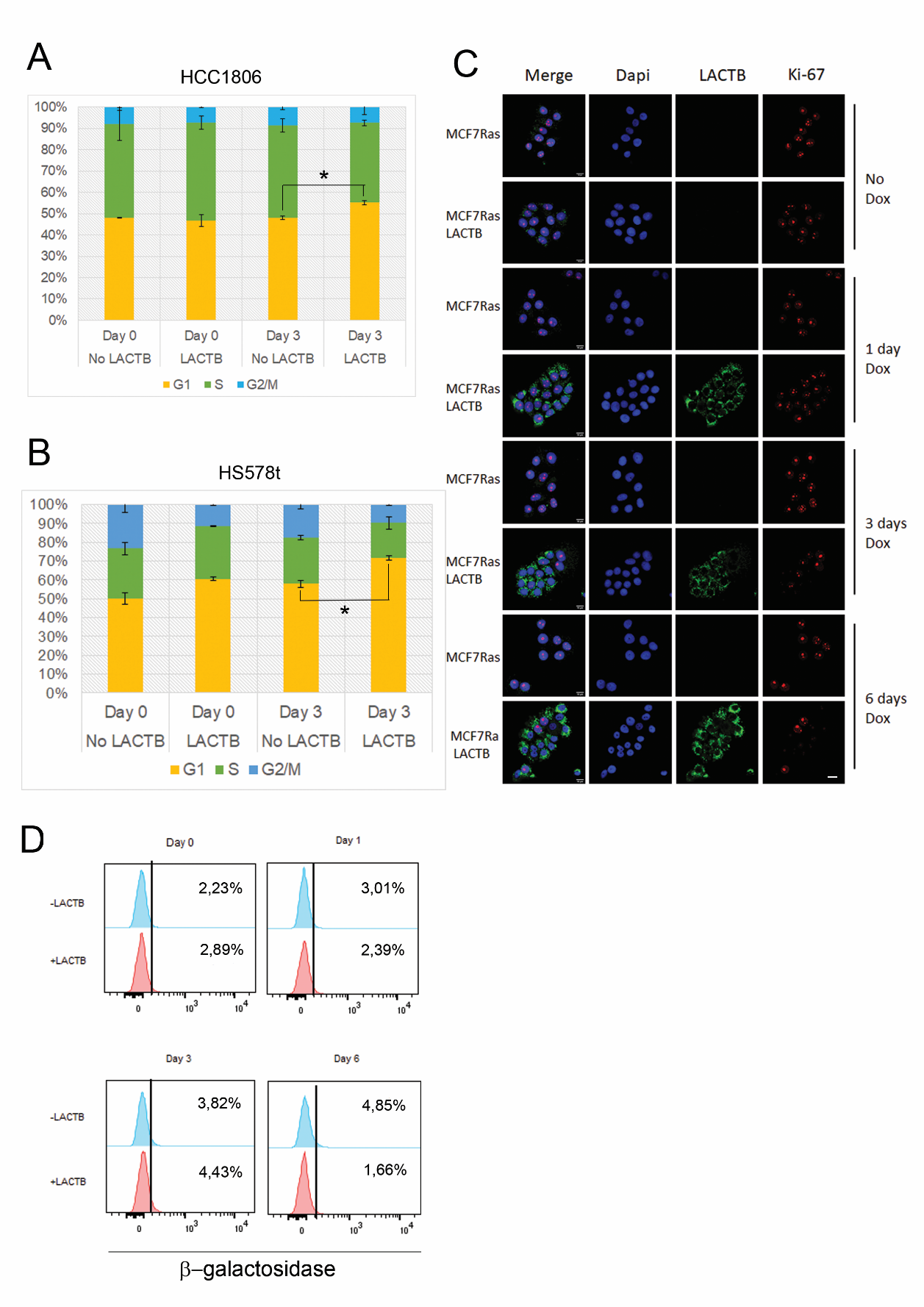


***Supplementary Figure 1: LACTB inhibits cell growth in other breast cancer cell lines.*** Cell cycle arrest analysis was performed by EdU in other breast cancer cells (HCC1806, A; HS578t, B). Graphs represent the percentage of cells in each phase of the cell cycle. (C) Immunofluorescence images of MCF7ras cells after LACTB overexpression. Proliferation marker Ki-67 is shown in red, LACTB in green and nucleus were stained with DAPI (blue). Scale bar: 10 μm. (D) Quantification of β-galactosidase activity by FACS analysis.
