# Supplementary material for: LACTB induces cancer cell death through the activation of the intrinsic caspase-independent pathway in breast cancer
Source: Apoptosis. 2022 Oct 25;28(1-2):186–98. doi: 10.1007/s10495-022-01775-4 (PMC9950249; doi:10.1007/s10495-022-01775-4)
Supplement: Supplementary file 14 — Supplementary Material 14 [file 10495_2022_1775_MOESM14_ESM.docx]

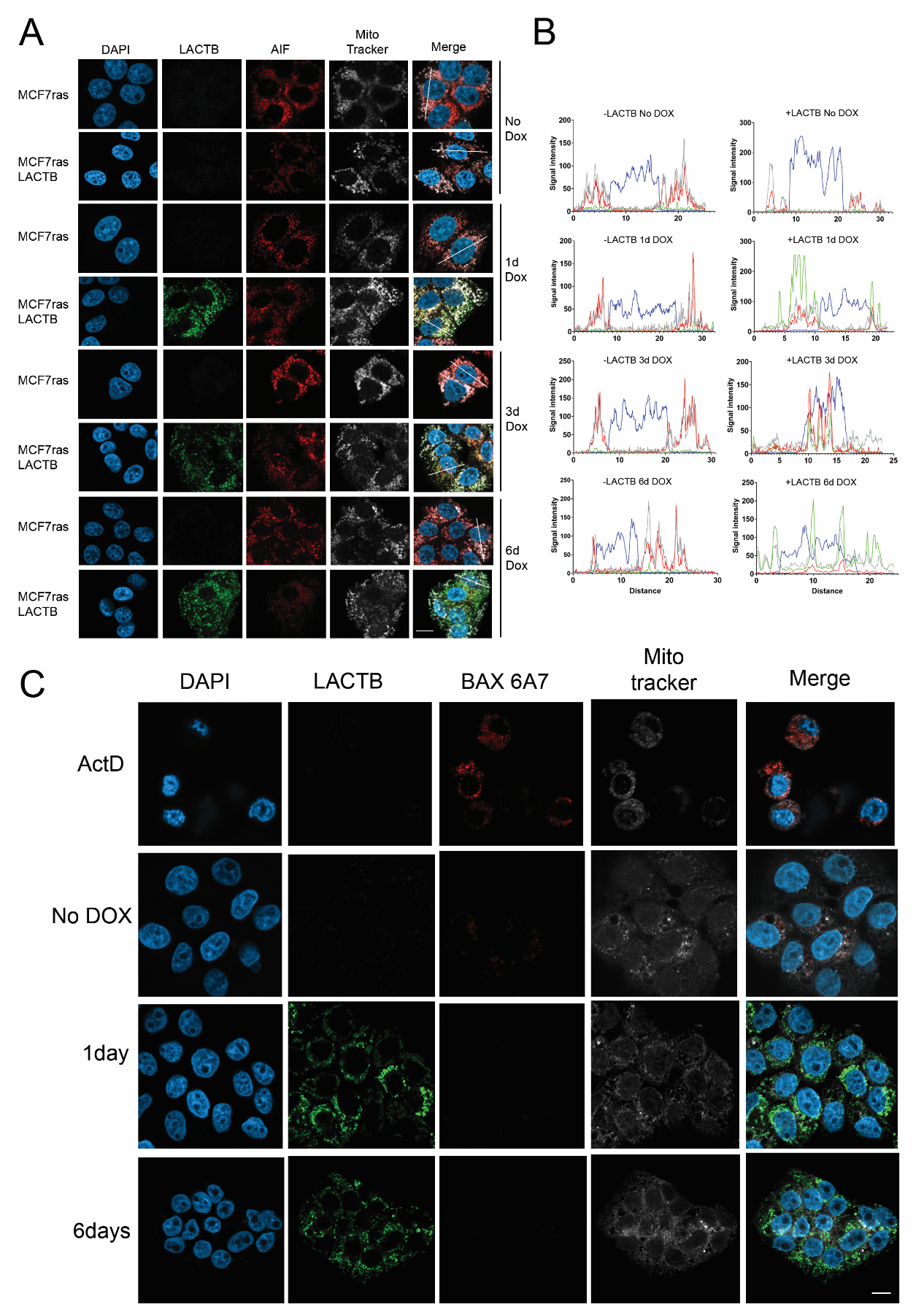


***Supplementary Figure 3: AIF is translocated from the mitochondria to the nucleus in a BAX-independent way.*** (A) AIF localization by confocal microscopy. Images were taken in a Zeiss confocal microscope and middle section of the cells for each channel is shown. (B) Profile signal intensity from (A). Profile analysis was done with ZEN 3.2 software. (C) Immunofluorescence in MCF7ras to detect activated BAX. LACTB was induced for the indicated time points. As a positive control of the pore formation by BAX, cells were treated with 15 nM of actinomycin D for 24 hours. A middle section of the cell is shown. Scale bar 10 μm.
