# Supplementary material for: LACTB induces cancer cell death through the activation of the intrinsic caspase-independent pathway in breast cancer
Source: Apoptosis. 2022 Oct 25;28(1-2):186–98. doi: 10.1007/s10495-022-01775-4 (PMC9950249; doi:10.1007/s10495-022-01775-4)
Supplement: Supplementary file 15 — Supplementary Material 15 [file 10495_2022_1775_MOESM15_ESM.docx]

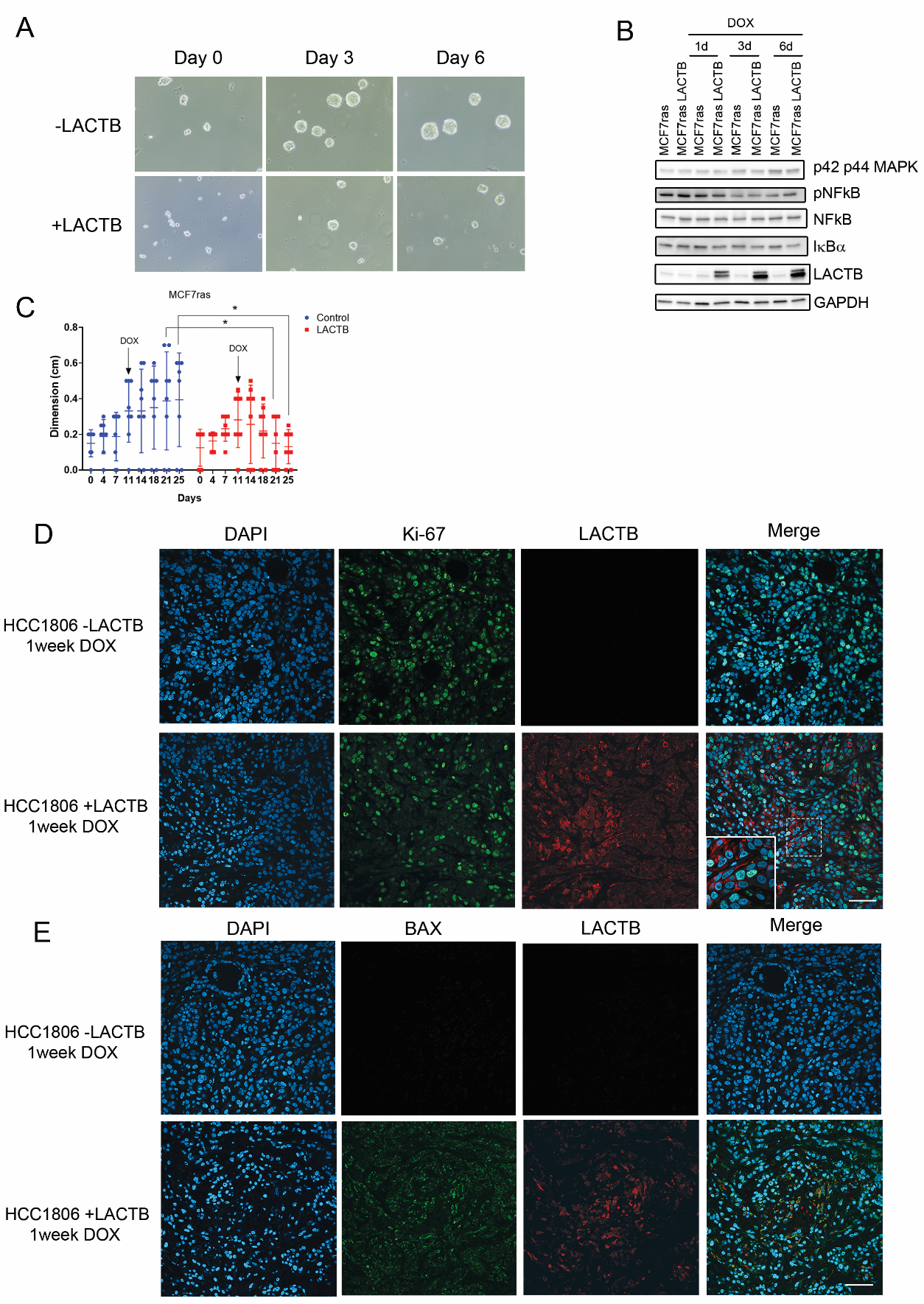


***Supplementary Figure 5: LACTB exerts an antiproliferative and proapoptotic behavior in 3D and in vivo conditions.*** (A) Microscopy images of MCF7ras spheres formed after 3 and 6 days. (B) Analysis by western blot of the spheres formed by MCF7ras for some proteins involved in prosurival pathways. (C) Tumour growth after LACTB induction in MCF7ras cells. *P<0,05. (D and E) Immunofluorescence of tumour tissue slices from HCC1806 for proliferation marker Ki-67 and proapoptotic marker Bax. Scale bar: 50 μm.
