# Supplementary material for: LACTB induces cancer cell death through the activation of the intrinsic caspase-independent pathway in breast cancer
Source: Apoptosis. 2022 Oct 25;28(1-2):186–98. doi: 10.1007/s10495-022-01775-4 (PMC9950249; doi:10.1007/s10495-022-01775-4)
Supplement: Supplementary file 19 — Supplementary Material 19 [file 10495_2022_1775_MOESM19_ESM.pdf]

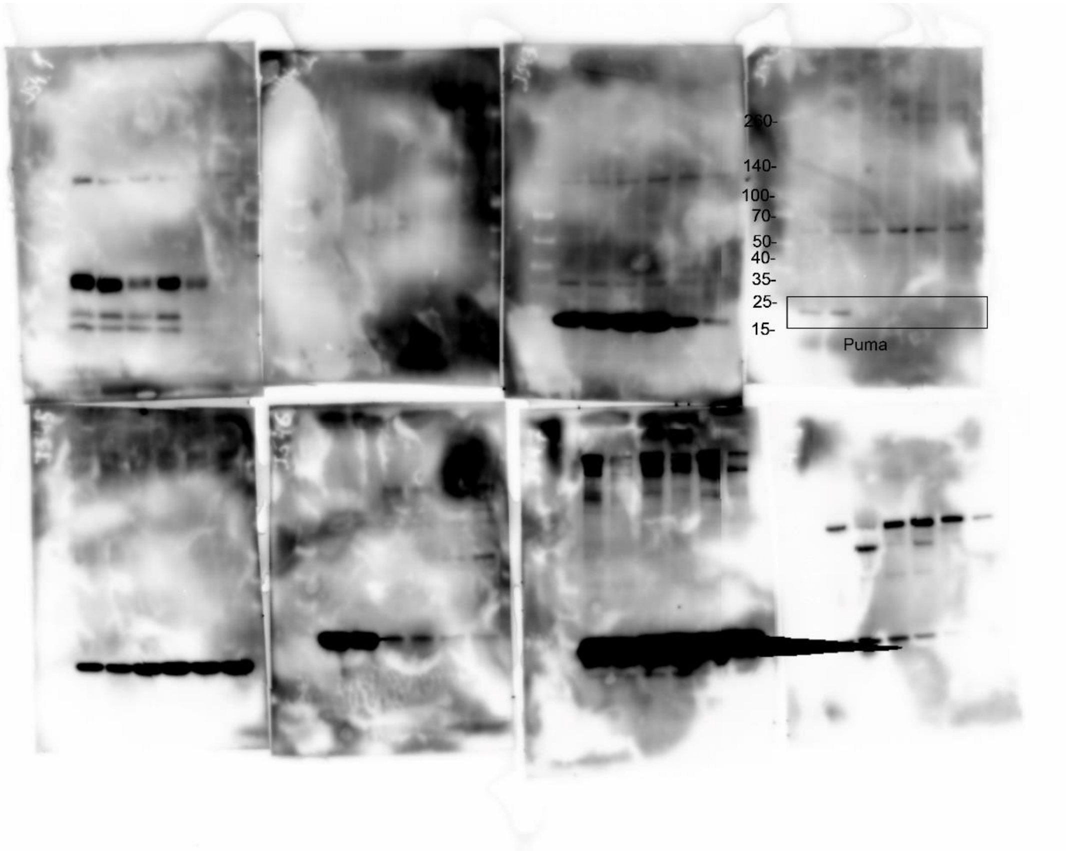
260-
140-
100-
70-
50-
40-
35-
25-
15-
Puma

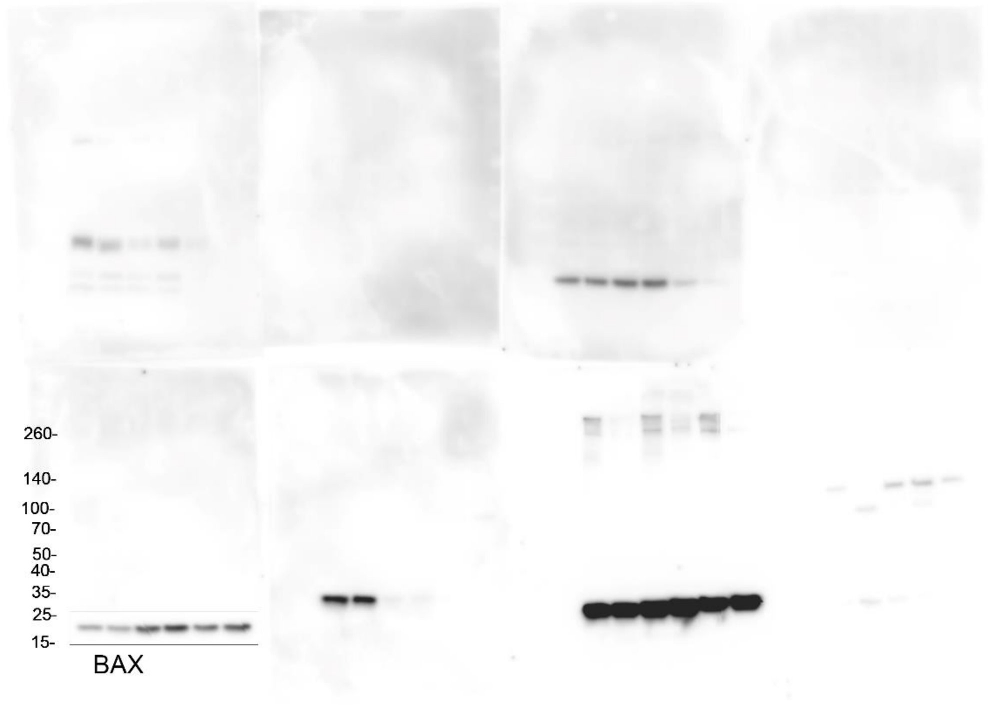
260-
140-
100-
70-
50-
40-
35-
25-
15-
BAX

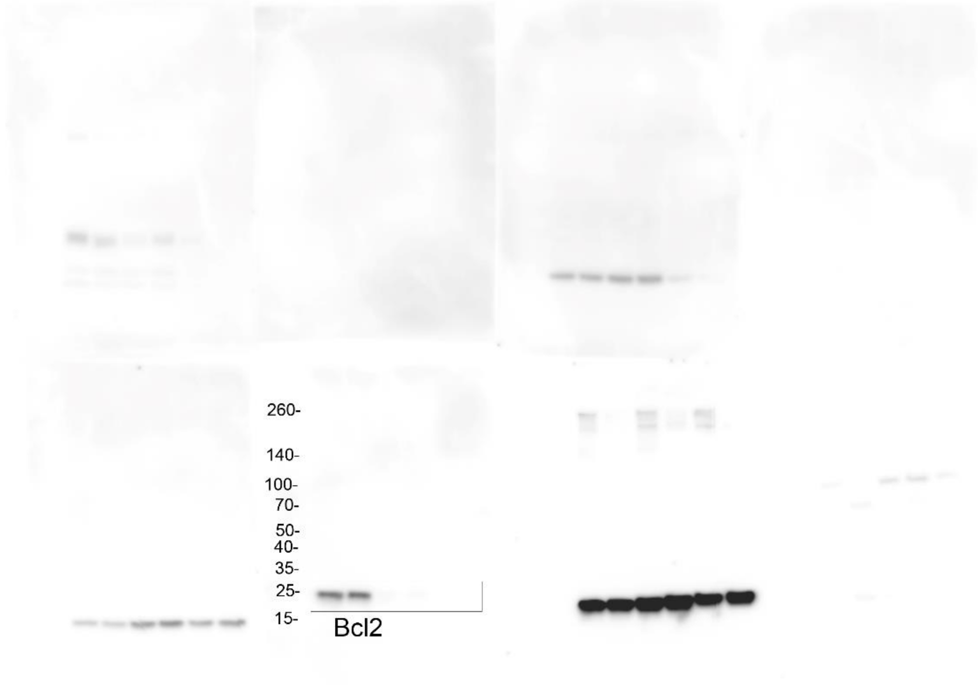
260-
140-
100-
70-
50-
40-
35-
25-
15-
Bcl2

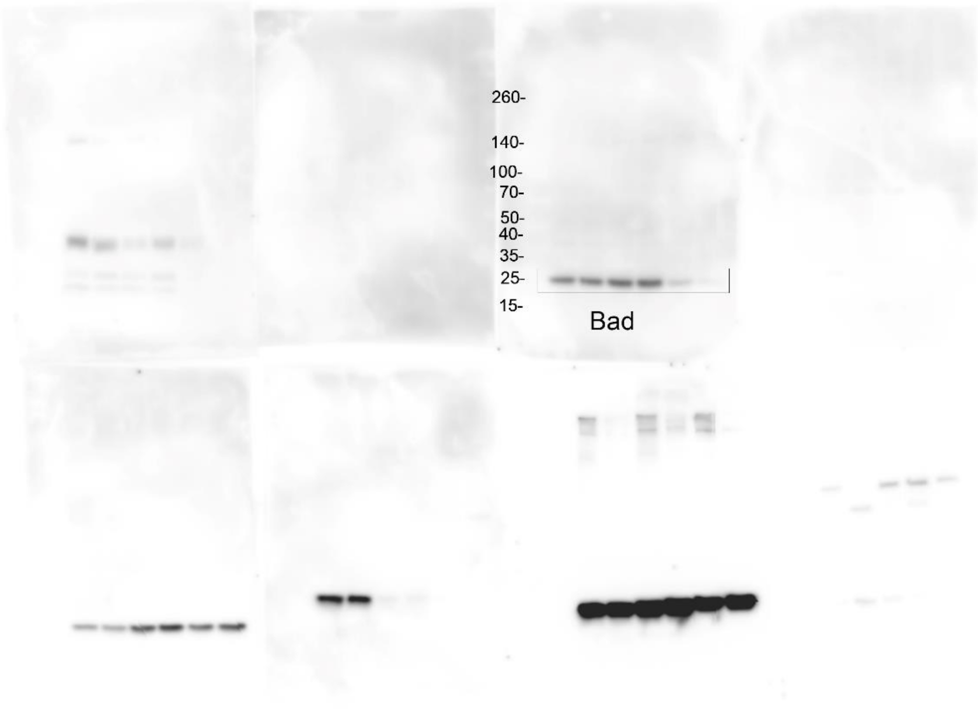
260-
140-
100-
70-
50-
40-
35-
25-
15-
Bad

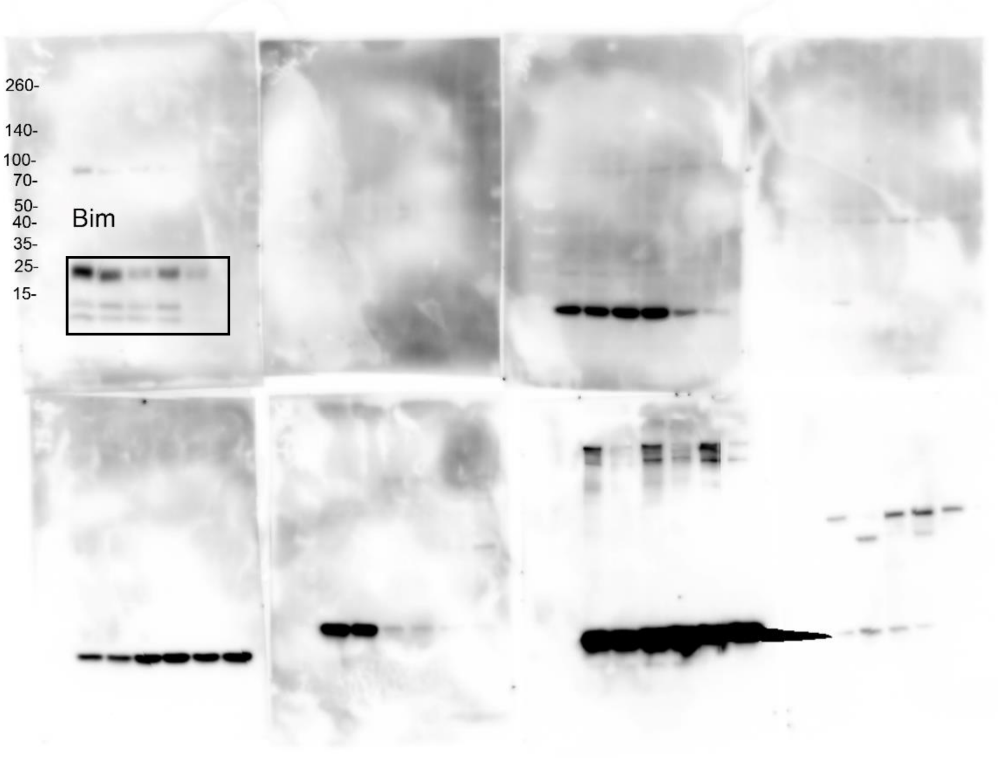
260-
140-
100-
70-
50-
40-
35-
25-
15-
Bim

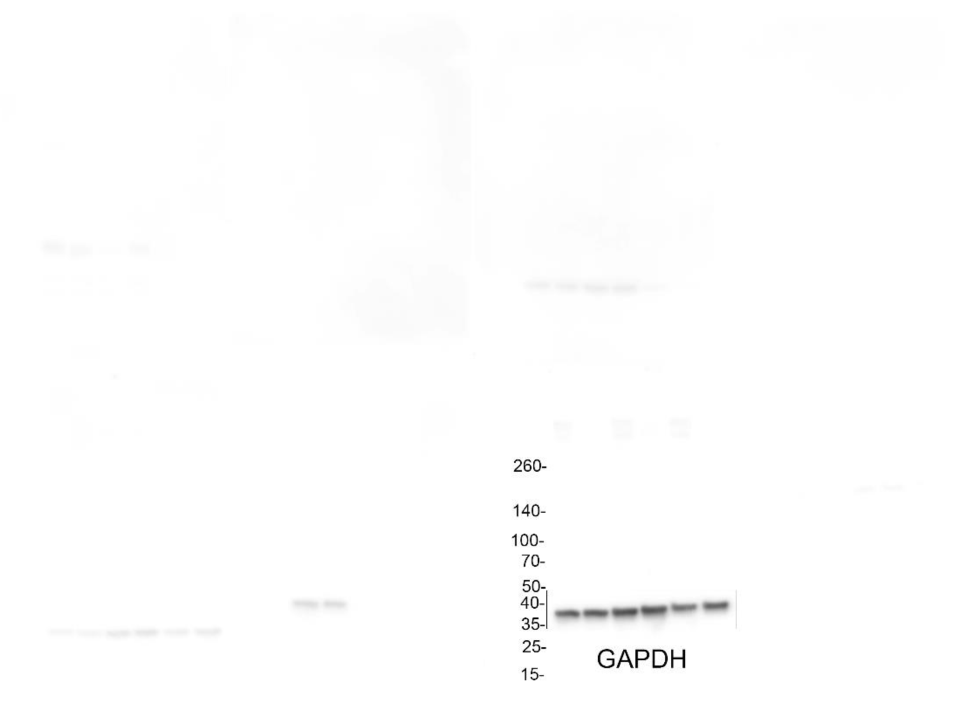
260-
140-
100-
70-
50-
40-
35-
25-
15-
GAPDH

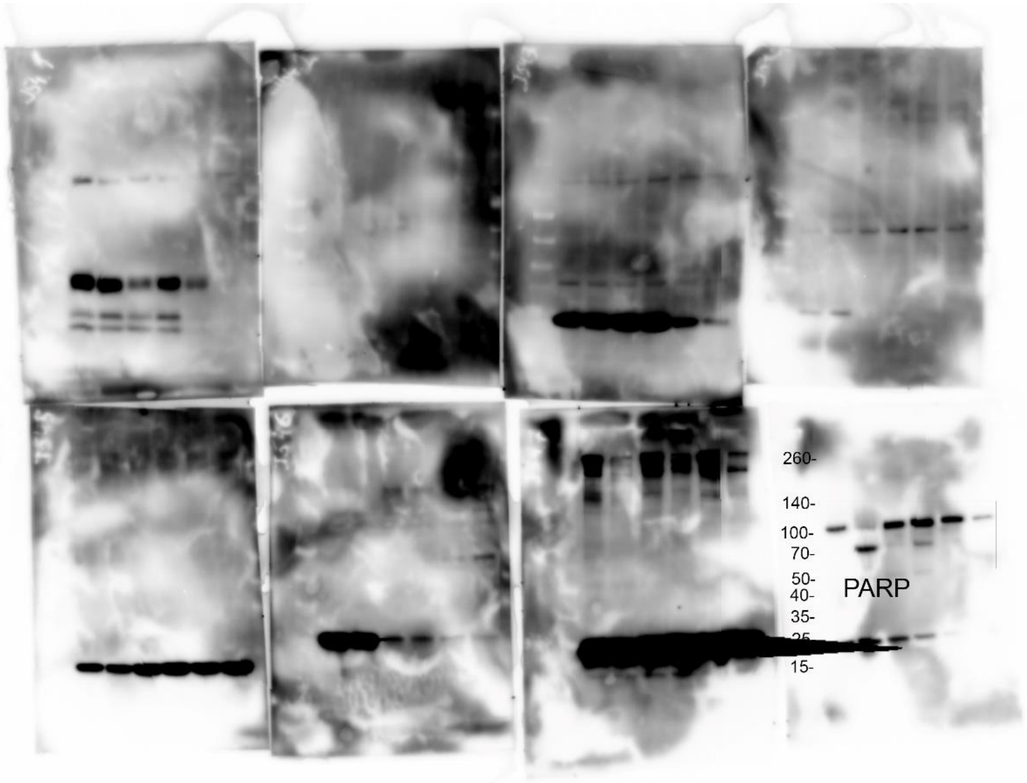
260-
140-
100-
70-
50-
40-
35-
25-
15-
PARP

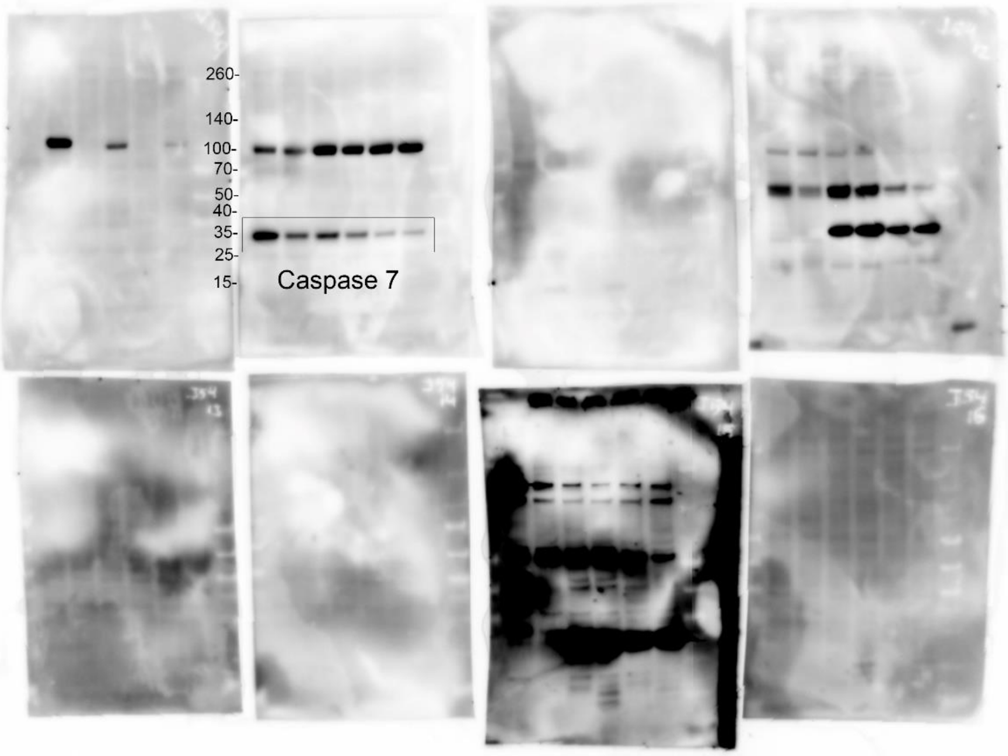
260-
140-
100-
70-
50-
40-
35-
25-
15-
Caspase 7

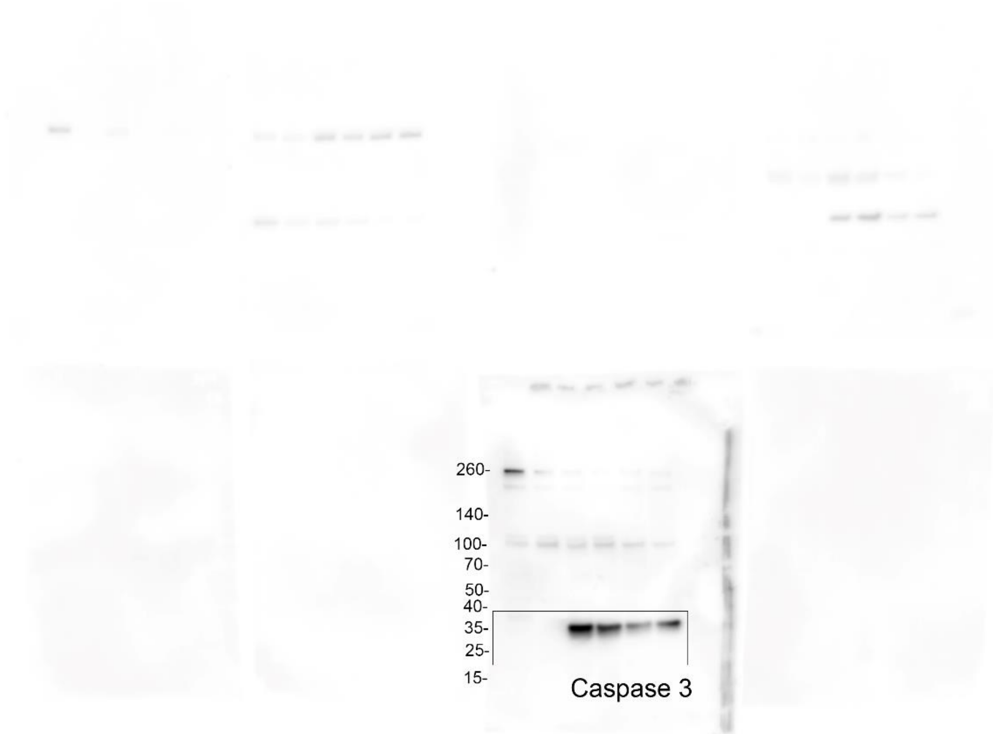
260-
140-
100-
70-
50-
40-
35-
25-
15-
Caspase 3

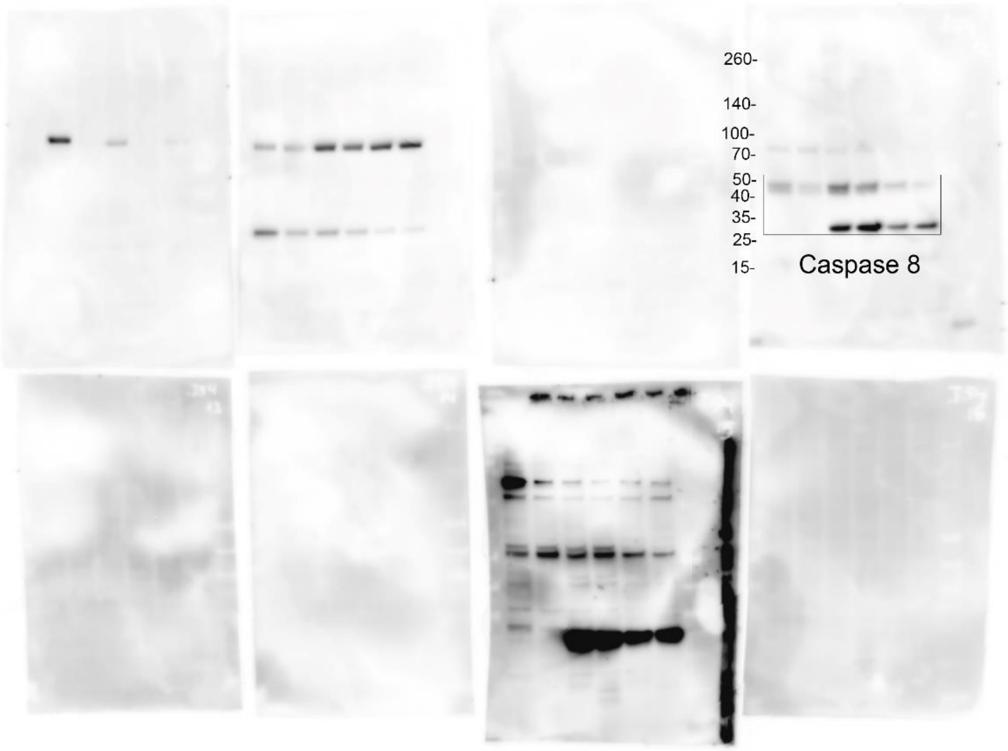
260-
140-
100-
70-
50-
40-
35-
25-
15-
Caspase 8

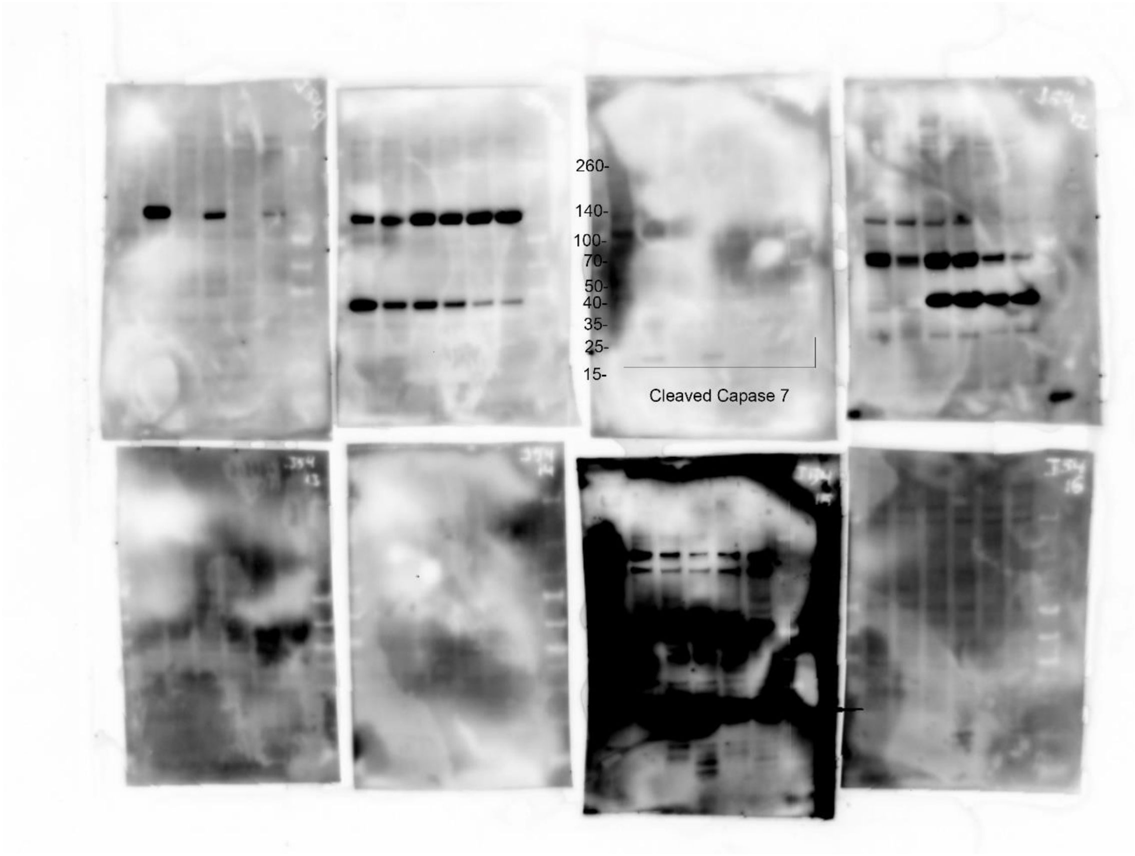
260-
140-
100-
70-
50-
40-
35-
25-
15-
Cleaved Capase 7

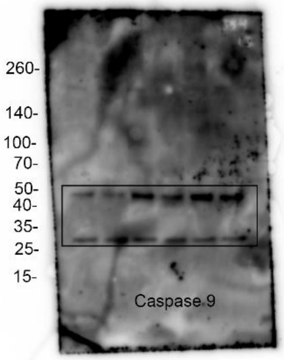

260-
140-
100-
70-
50-
40-
35-
25-
15-
Caspase 9
